# Cross-coupling of CO and an isocyanide mediated by a tetrameric magnesium hydride cluster†

**DOI:** 10.1039/d4sc02638a

**Published:** 2024-06-24

**Authors:** Wenbang Yang, Andrew J. P. White, Mark R. Crimmin

**Affiliations:** a Department of Chemistry, Molecular Sciences Research Hub 82 Wood Lane, Shepherds Bush London W12 0BZ UK m.crimmin@imperial.ac.uk

## Abstract

Sequential addition of CNXyl (Xyl = 2,6-dimethylphenyl) and CO to a tetrametallic magnesium hydride cluster results in stepwise reduction and cross-coupling of these substrates. Cross-coupling results in the formation of an ethene amidolate ligand [OC^1^(H^1^)=C^2^(H^2^)NAr]^2−^ a previously unknown entity which contains a 1,2-difunctionalised carbon chain reminiscent of those found in aminoalcohols and amino acids. To the best of our knowledge, this is the first example of such reactivity with metal hydride precursors. DFT calculations support a mechanism that parallels that established for coupling of CO to form ethenediolate ligands, with the key carbon–carbon bond step occurring by nucleophilic attack of a putative azamethylene intermediate on CO. The cluster plays a key role in templating the synthesis, providing kinetic control over each of the steps. The ethene amidolate ligand can be transferred to other metals (Al) and semi-metals (B) through onwards metathesis reactions.

## Abstract 2

Sequential addition of CNXyl (Xyl = 2,6-dimethylphenyl) and CO to a tetrametallic magnesium hydride cluster results in stepwise reduction and cross-coupling of these substrates.

## Graphical abstract

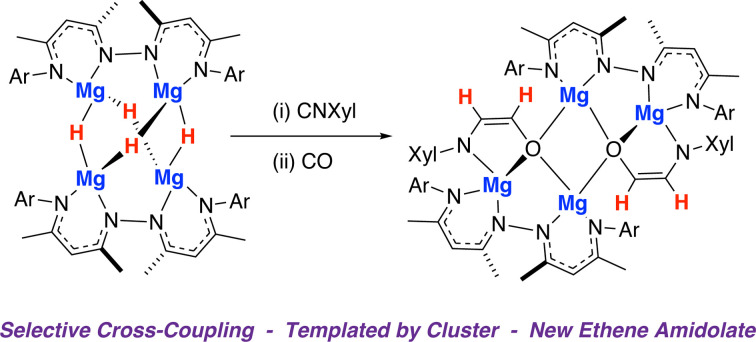

## Introduction

**Fig. 1:**
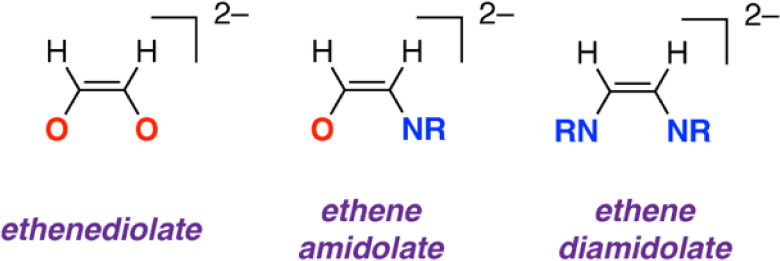
Ethenediolate, ethene amidolate, and diamidolate structures based on two carbon units.

Carbon monoxide (CO) is an attractive C_1_ building block for the construction of more complex organic molecules. In the past few years there has been increased interest in reactions that combine two (or more) molecules of CO to construct carbon chains.^1–5^ Controlling selectivity in these reactions is important; both the chain length and the ratio of C : H : O are key factors. Focusing on C_2_ products, it has been shown that two equivalents of CO can be combined at reducing metal centres to form ethynediolates,^6–12^ ethenediolates,^13–19^ ethanediolates,^20^ ketenes,^21^ an acetaldehyde enolate,^22^ and ethene.^23^ Isocyanides (CNR, R = alkyl, aryl) are isoelectronic with CO. Related reactions that combine two or more equivalents of isocyanide to form homocoupled products are widely reported (Fig. 1).^24–38^ When considering these studies, an obvious question arises as to whether it might be possible to combine CO and CNR to form cross-coupled products. For the C_2_ series this would potentially lead to the formation of products containing a 1,2-difunctionalised carbon chain with vicinal oxygen and nitrogen containing functional groups. Such substitution patterns are found in amino acids, β-aminoketones, and 1,2-aminoalcohols and are common across natural products, medicinally relevant compounds, and privileged ligand structures.

Precedent for the cross-coupling of CO with CNR is limited to a handful of examples. Stepwise reaction of CO and CNR with a zirconium(iv) dialkyl complex to form a substituted ethene amidolate was reported as early as 1985.^39^ This type of reactivity has been observed at group 4 metal dialkyl complexes supported by alkoxide,^40^ tropocoronand,^41^ and *N*-heterocyclic carbene ligands.^42^ Related titanocyclobutane complexes,^43^ and seven coordinate niobium and tantalum complexes have also been reported to react with CO and CNR to form substituted ethene amidolates.^44,45^ In contrast, a thorium alkyl complex was shown to react with CO and CNR to form a ketenimine species.^46,47^ Binuclear silylene compounds are known to cross-couple and deoxygenated CO and CNR to form functionalised ketenimine products.^48^ To the best of our knowledge, despite nearly 40 years of interest in these types of reactions, cross-coupling reactions of CO and CNR at main group metals are unknown. All examples of cross-coupling reported to date employ low-oxidation state compounds or metal alkyl complexes. There are no examples of reactions that employ metal hydrides, meaning the parent ethene amidolate ligand [OCH=CHNR]^2−^ (R = alkyl, aryl) is an unknown chemical entity.

In this paper, we report the stepwise cross-coupling of CO and CNXyl (Xyl = 2,6-dimethylphenyl) using a tetrametallic magnesium hydride complex. This reaction results in the generation of the simplest known ethene amidolate ligand. A dimeric magnesium hydride complex was also investigated in CO and CNXyl cross-coupling, but did not lead to the isolation of ethene amidolate product, suggesting that the tetrameric metal cluster plays an important role in templating reactivity. DFT calculations are used to probe the electronic structure of the coordinated ethene amidolate fragment and rationalise the most likely mechanism of its formation. Finally, we show that the ethene amidolate ligand is labile and can be transferred to alternative metals and semi-metals (*e.g.* Al, B) providing access to a unique 1,2-difunctionalised C_2_ ligand derived from CO.

## Results and discussion

### Cross-coupling of CO and CNXyl

The tetrametallic magnesium hydride cluster 1a was originally reported by Harder and co-workers.^49^ Related species have recently been documented from Kretschmer's group.^50^ Previously we showed that 1a reacts with two equiv. CO by a deoxygenative pathway leading to the formation of a coordinated acetaldehyde enolate.^22^

**Scheme 1:**
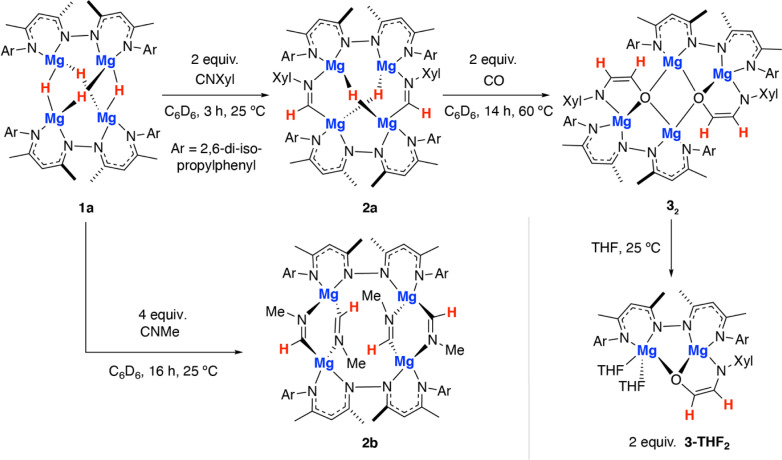
Reactions of a tetrametallic magnesium hydride cluster 1a with isocyanides and CO.

**Fig. 2:**
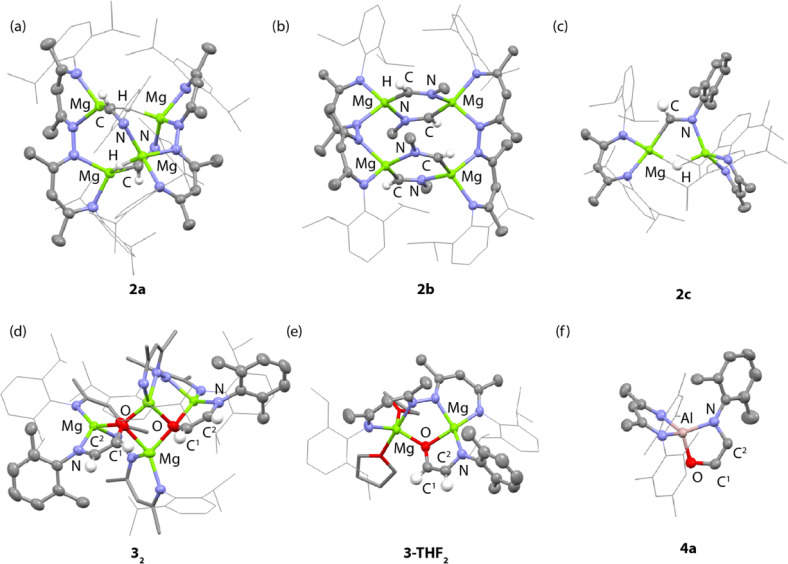
X-ray structures of (a) 2a, (b) 2b, (c) 2c, (d) 3_2_, (e) 3-THF_2_ and (f) 4a. Aryl groups on the β-diketiminate ligands are drawn as wireframe. Hydrogen atoms with the exception of hydride ligands have been omitted for clarity.

Reaction of 1a with 2-6-dimethylphenylisocyanide for 3 h at 25 °C in benzene led to selective formation of the bis(imine) complex 2a derived from a 2 : 1 reaction stoichiometry (Scheme 1). Insertion of the isocyanide occurs exclusively at only one of the two possible types of hydride sites in 1a. 2a was characterised by diagnostic resonances in the ^1^H NMR spectrum (C_6_D_6_) at *δ* = 8.83 (s) and 3.38 (s) ppm each integrating to 2H and assigned to the CH=NAr imine proton and unreacted hydride ligands respectively. The former resonance shows a cross-peak in the HSQC data to a ^13^C resonance at *δ* = 157.7 ppm assigned to the CH=NAr imine carbon atom. In the solid-state, 2a retains a tetrametallic structure with the dinucleating ligands templating a close arrangement of four magnesium atom in an approximate tetrahedral arrangement (Fig. 2a). The newly formed imine ligands adopt a κ^2^-C, N coordination mode, bridging two of the magnesium sites of the tetrahedra, the remaining two metals are bridged by the unreacted hydrides. The Mg–C bond lengths of the metalated imine are 2.1778(17) and 2.1830(17) Å, while the Mg–N bond lengths are 2.1185(15) and 2.1428(14) Å.

In contrast, addition of 4 equiv. of methylisocyanide to 1 led to the formation of 2b, derived from exhaustive insertion of the unsaturated substrate into all four hydride sites (Scheme 1, Fig. 2b). It remains likely that variation of the sterics of the substituent of the isocyanide directly influences the outcome.

Onwards reaction of 2a with CO for 14 h at 60 °C in benzene led to 3_2_ (Scheme 1, Fig. 2d). 3_2_ could also be prepared in a single-pot reaction from 1a by stepwise addition of CNXyl then CO. 3_2_ contains two ethene amidolate ligands derived from cross-coupling of an isocyanide and CO. Each new C_2_ fragment in 3_2_ originates from a 2 : 1 : 1 reaction of hydride : CO : CNXyl. This is the first example of this reactivity at a metal hydride complex and as such the first time a simple unsubstituted ethene amidolate ligand, [OC^1^(H^1^)=C^2^(H^2^)NAr]^2−^, has been observed to form from CO. In C_6_D_6_ solution, 3_2_ was characterised by diagnostic, non-equivalent, protons of the ethene amidolate moiety H^1^ and H^2^ which resonate at *δ* = 5.03 (d, ^3^*J*_H–H_ = 2.2 Hz) and 6.29 (d, ^3^*J*_H–H_ = 2.2 Hz) ppm. These correlate with ^13^C environments C^1^ and C^2^ at *δ* = 102.9 and 152.2 ppm respectively. Reaction of 2a with ^13^CO allowed synthesis of ^13^C-3_2_. ^13^C-3_2_ is monotopically labelled at the C^1^ position and there is no evidence for inclusion of the isotope at the C^2^ position of the ligand or elsewhere. The ^1^*J*_C–H_ and ^2^*J*_C–H_ coupling constants take values of 139.2 and 10.8 Hz respectively. In the solid-state, 3_2_ again retains a tetrametallic structure albeit with loss of the tetrahedral arrangement of the magnesium sites. The structure of 3_2_ is perhaps best conceptualised as a dimer of two dimagnesium fragments related through an inversion symmetry operation. Each dimagnesium fragment is coordinated by the dinucleating bis(β-diketiminate) ligand and the ethene amidolate which coordinates one magnesium atom κ^2^-O,N and the other in a κ^1^-O fashion. The two dimagnesium fragments dimerise to form an open tetrametallic cluster with the four magnesium sites orientated in a butterfly-like configuration. Dimerisation occurs *via* bridging oxygen atoms of the ethene amidolate, such that these end up adopting a μ^3^-bridging mode. Consistent with the small coupling constant between the H^1^ and H^2^ positions, the ethene amidolate adopts a *cis*-configuration. The ethene amidolate is formally a dianionic ligand, bond lengths to Mg are unremarkable. The C^1^–O, C^2^–N, and C^1^=C^2^ bond lengths take values of 1.421(3), 1.357(3) and 1.375(3) Å respectively.

Dissolving crystalline samples of 3_2_ in THF followed by analysis of the resultant sample by ^1^H NMR spectroscopy, either in C_6_D_6_ or THF, led to significant changes of the resonances assigned to the ethene amidolate ligand, with H^1^ and H^2^ now resonating at *δ* = 5.42 (d, ^3^*J*_H–H_ = 3.4 Hz) and 5.32 (d, ^3^*J*_H–H_ = 3.4 Hz) ppm respectively. Recrystallisation revealed the formation of 3-THF_2_, a product of fragmentation of the tetrametallic structure into a single dimagnesium motif (Scheme 1, Fig. 2e). In the solid state, THF preferentially solvates only one of the magnesium centres, leading to an asymmetric structure containing both 4- and 5-coordinate metals. The C^1^–O, C^2^–N, and C^1^=C^2^ bond lengths of the ethene amidolate are near identical to those observed in 3_2_. The reaction is non-reversible and attempts to remove THF from 3-THF_2_ under high vacuum (1 × 10^−4^ mbar) did not lead to reformation of 3_2_.

DFT calculations were conducted. Geometries were optimised with the B3PW91-D3 functional using a hybrid basis-set comprised of 6-31G** (C,H,N,O) and SDDAll (Mg). Solvation was taken into account in the optimisation using the polarizable continuum model (benzene). Single point corrections to energies were conducted with triple-zeta basis set 6-311+G** applied to all energies. These calculations suggest that the fragmentation of 3_2_ to 2 equiv. 3 is disfavoured in the absence of a coordinating solvent (Δ*G*_298 K_° = +46.2 kcal mol^−1^; Δ*H*° = +66.0 kcal mol^−1^). Deaggregation becomes more favourable in the presence of THF with conversion of 3_2_ + 4 THF → 2 3-THF_2_ being only modestly endergonic (Δ*G*_298 K_° = +11.5 kcal mol^−1^; Δ*H*° = −19.2 kcal mol^−1^). In the presence of an excess THF (solvent) the equilibrium is likely displaced to the products, allowing the isolation of 3-THF_2_ (see ESI, Fig. S19†). DOSY NMR studies suggest that the nuclearity of 3_2_ and 3-THF_2_ observed in the solid-state is potentially retained in solution. In C_6_D_6_ at 298 K the diffusion coefficient of 3_2_ was measured as 7.8 × 10^−10^ m^2^ s^−1^ while 3-THF_2_ in THF-d_8_ solution diffused at a substantial faster rate of 1.1 × 10^−9^ m^2^ s^−1^ (see ESI, Fig. S9 and S10†). The data correspond to estimated hydrodynamic radii of 9.3 Å for 3_2_ and 7.2 Å for 3-THF_2_ consistent with 3_2_ retaining its tetrametallic structure in solution.

### Electronic structure of the ethene amidolate ligand

**Fig. 3:**
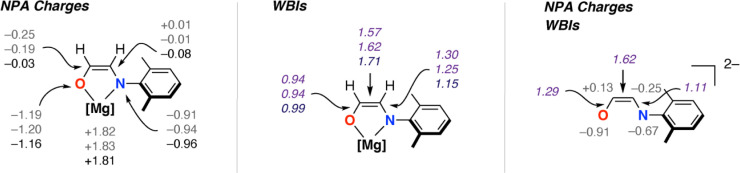
Calculated NPA charges and Wiberg Bond Indices for 3_2_ and 3-THF_2_ (data are listed with values from 3_2_ top and middle and 3-THF_2_ bottom) alongside comparison to data calculated for the parent dianion. NBO calculations conducted in G09 using NBO 6.0. B3PW91-D3/6-311+G**/PCM (benzene).

The new ethene amidolate motif [OC^1^(H^1^)=C^2^(H^2^)NAr]^2−^ fills a gap between ethenediolate [OC(H)=C(H)O]^2−^ and ethenediamidolate [ArNC(H)=C(H)NAr]^2−^ ligands (Fig. 1). NBO calculations (see ESI† Section 4.3) were undertaken on 3_2_ and 3-THF_2_ to probe the electronic structure of this species and nature of its binding to magnesium (Fig. 3). For comparison, the parent [OC^1^(H^1^)=C^2^(H^2^)NAr]^2−^ was calculated as a non-coordinated dianion. The ethene amidolate adopts a conjugated structure. For 3_2_ the Wiberg Bond Indices (WBIs) take values of C^1^=C^2^ (1.57, 1.62), C^1^–O (0.94, 0.94) and C^2^–N (1.25, 1.30). While in the broadest sense, these data can be interpreted in terms of an alternating array of single and double bonds for the ethene amidolate structure, the C–N WBIs are slightly greater than expected for a single bond suggesting a degree of delocalisation of the N lone-pair into the conjugated system. Binding to magnesium is primarily ionic with NPA charges on Mg (+1.82, +1.83) being highly positive and those on O (−1.19, −1.20) and N (−0.91, −0.94) uniformly negative. There also appears to be some differences in the degree of this delocalisation across the different amidoenolates in the calculated structures 3_2_ and 3-THF_2_ suggesting the ligand can respond and augment its polarisation dependent on the local environment. The molecular orbitals of [OC^1^(H^1^)=C^2^(H^2^)NAr]^2−^ are consistent with its potential to act as both a σ- and π-donor ligand, with the HOMO, HOMO-1 and HOMO-2 showing character of both oxygen and nitrogen-based lone-pairs in the plane and orthogonal to the site of metal coordination (see ESI, Fig. S20–S22†).

### Mechanism

**Fig. 4:**
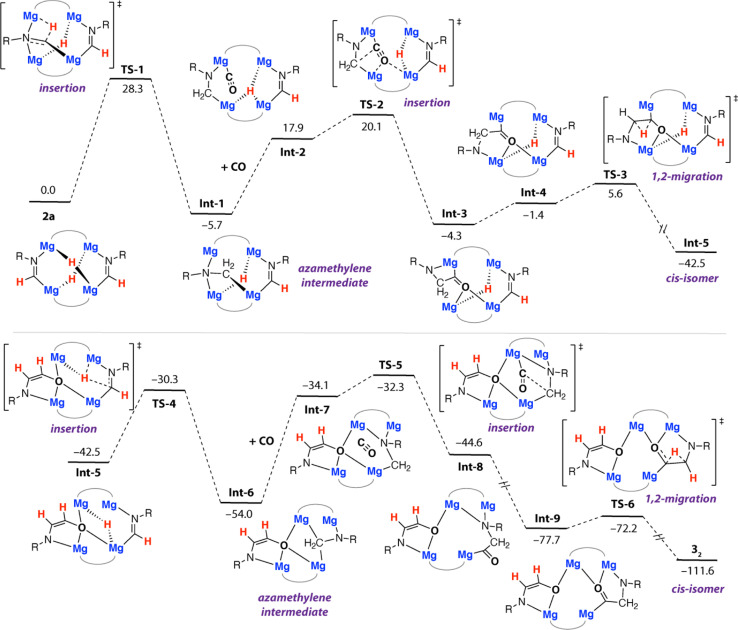
Calculated pathway for the formation of 3_2_ from 2a. R = Xyl. B3PW91-D3/6-311+G**/PCM (benzene)//B3PW91-D3/6-31G** (C,H,N,O)/SDDAll (Mg)/PCM (benzene). Gibbs energies, values in kcal mol^−1^.

Further calculations were undertaken to shed light on the mechanism that leads to the formation of 3_2_. For simplicity, these calculations were initiated from 2a as the reactant. We have previously calculated insertion of CO into two of the Mg–H bonds of 1a and proposed structures similar to 2a in the homologation and deoxygenation of CO using this tetrametallic cluster.^22^ A plausible pathway for ethene amidolate formation was found (Fig. 4). This pathway parallels the most common pathway described for ethenediolate formation in reactions of metal hydrides with CO.^51,52^

The pathway is initiated by an intramolecular hydromagnesiation (or hydride transfer) to one of the metallated imine ligands of 2a. This step occurs by TS-1 (Δ*G*^‡^_298 K_ = 28.3 kcal mol^−1^) and leads directly to an azamethylene intermediate Int-1. The structure of Int-1 parallels established oxymethylene intermediates in CO homologation. CO coordination to Int-1 forms Int-2, with subsequent insertion into a Mg–C bond of the azamethylene motif occurring *via*TS-2 (Δ*G*^‡^_298 K_ = 25.8 kcal mol^−1^). TS-2 leads directly to Int-3 and is the key carbon–carbon bond forming step that creates the 1,2-difunctionalised carbon chain. TS-2 appears late on the potential energy surface, C^1^⋯C^2^ bond forming is advanced and occurs with nucleophilic attack of the azamethylene carbon C^2^ on C^1^ of the coordinated CO ligand. 1,2-Hydride migration can then occur from Int-3. Int-3 can isomerise to Int-4 through rotation about the C^1^–C^2^ bond, this step necessarily occurs with decoordination of the nitrogen atom from one Mg site and recoordination to another. Despite our best efforts we were unable to identify a transition state for this process, based on scans of the potential energy surface we suggest the rearrangement is extremely facile. 1,2-Hydride migration from Int-4 can occur by the low energy TS-3 and leads directly to the *cis*-isomer of the ethene amidolate ligand, Int-5. The sequence of hydromagnesiation, CO insertion, and 1,2-hydride migration then repeats ultimately leading to the experimentally determined product 3_2_. Analysis of the complete pathway for formation of 3_2_ reveals that the formation of the 1st and 2nd ethene amidolate ligands occurs with similar barriers, consistent with the lack of observable intermediates in the conversion of 2a to 3_2_. The global barrier is associated with the 1st hydride transfer step from 2a and is Δ*G*^‡^_298 K_ = 28.3 kcal mol^−1^ which is in reasonable agreement with the experimental conditions for the reaction (14 h, 60 °C).

**Scheme 2:**
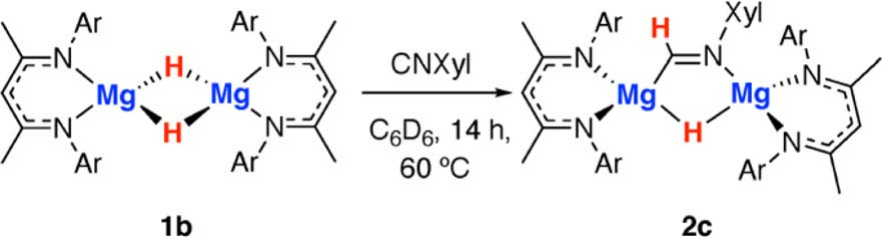
Reaction of a magnesium hydride dimer 1b with an isocyanide to form 2c.

Throughout the calculated pathway the tetrametallic cluster templates bond making and breaking events and adapts its geometry to ultimately accommodating the ligands in the most thermodynamic favourable binding sites. Curious as to whether the tetrametallic structure was essential for the observed reactivity, we revisited the reactions of dimeric magnesium hydrides with isocyanides. Previously it has been shown that 1b reacts with cyclohexyl isocyanide and *tert*-butyl isocyanide to form double insertion products.^13,53^ In our hands, reaction of 1b with 2-6-dimethylphenylisocyanide in C_6_D_6_ for 14 h at 60 °C allowed the isolation of the mono-insertion product 2c (Scheme 2, Fig. 2c). Like the tetrametallic analogue 2a, 2c still contains a single unreacted hydride site and hence based on our postulated mechanism, could potentially react with CO to generate an ethene amidolate ligand. Addition of CO to 2c and monitoring the reaction for 14 h at 25 °C provided no evidence for carbon–carbon bond formation. Over this time, 2c was fully consumed and a complex mixture was formed, suggesting that if an ethene amidolate ligand is generated it is only transient and reacts further under the conditions of the experiment.

### Transfer of the ethene amidolate ligand

**Scheme 3:**
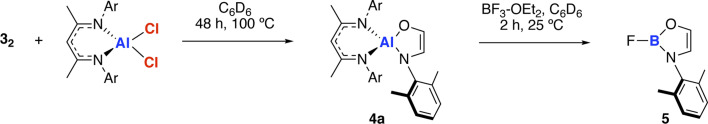
Transfer of the ethene amidolate ligand from 3_2_ to aluminium forming 4a and boron forming 5.

The amidoenolate ligand of 3_2_ could be transferred to alternative metals and semi-metal through metathesis reactions (Scheme 3, Fig. 2f). These reactions generated products in which the amidoenolate is coordinated to aluminium (4a) and boron (5). Transfer of the amidoenolate is driven by the highly ionic interaction with magnesium and thermodynamically favourable formation of the corresponding magnesium chloride byproduct, which can be separated by fractional crystallisation. NMR spectroscopic data along with single crystal X-ray diffraction studies suggest that the amidoenolate remains bound as a κ^2^-O,N ligand in both 4a and 5.

## Conclusions

In summary, stepwise reaction of CNXyl and CO with a tetrametallic magnesium hydride cluster results in reduction and cross coupling of the substrates to form a unique ethene amidolate ligand. This new C_2_ fragment contains a 1,2-difunctionalised motif. Prior work has established that this type of reactivity is possible with early transition metal alkyl complexes or low-valent silicon complexes, but to the best of our knowledge this is the first example involving a metal hydride precursor. A metalated imine complex (2a) was identified as an intermediate in cross-coupling. Data suggest the formation of this species at the magnesium cluster is under strict kinetic control as variation of either the isocyanide or magnesium complex leads to alternative products that are non-productive in cross-coupling. DFT calculations support a mechanism that evolves from the metalated imine to an azamethylene intermediate by a second hydride transfer. Carbon–carbon bond formation can occur by nucleophilic attack of the azamethylene on CO, with subsequent 1,2-hydrogen migration establishing the ethene amidolate structure. This pathway closely parallels that established for ethenediolate formation from CO and metal hydride complexes. Onwards reaction of the cross-coupled product (3_2_) has established that the ethene amidolate ligands can be transferred to alternative fragments by metathesis reactions. The discovery that metal hydride complexes are competent in cross-coupling of isocyanides and CO could inspire new approaches toward the synthesis of amino alcohols and amino acids from inexpensive and readily available C_1_ building block.

## Data availability

Synthetic procedures, kinetic experiments, NMR spectra of all compounds, crystal structures of 2a, 2b, 2c, 3_2_, 3-THF_2_, and 4a, crystallographic data, and computational methods (PDF). Cartesian coordinates of the DFT-optimized structures (XYZ) are available as part of the ESI. CCDC 2289252, 2308757–2308759, 2313439 and 2328581 contain the supplementary crystallographic data for this paper.†

## Author contributions

WY conducted all experimental and computational work. AJPW collected and refined single crystal data. All authors were involved in writing the manuscript.

## Conflicts of interest

The authors declare no conflicts of interest.

## Supplementary Material

SC-015-D4SC02638A-s001

SC-015-D4SC02638A-s002

SC-015-D4SC02638A-s003
