# Supplementary material for: Cross-coupling of CO and an isocyanide mediated by a tetrameric magnesium hydride cluster
Source: Chem Sci. 2024 Jun 24;15(30):11807–13. doi: 10.1039/d4sc02638a (PMC11290425; doi:10.1039/d4sc02638a)
Supplement: SC-015-D4SC02638A-s002 [file SC-015-D4SC02638A-s002.pdf]

Electronic Supplementary Material (ESI) for Chemical Science.
This journal is © The Royal Society of Chemistry 2024

Supporting Information

Cross-Coupling of CO and an Isocyanide Mediated by a Tetrameric Magnesium Hydride Cluster

Wenbang Yang, Andrew J. P. White, and Mark R. Crimmin*

m.crimmin@imperial.ac.uk

Department of Chemistry, Molecular Sciences Research Hub, 82 Wood Lane, Shepherds Bush, London, W12 0BZ, UK.
m.crimmin@imperial.ac.uk

Table of Contents

1) General Experimental **S3**

2) Experimental Methods **S4**

2.1) Preparation of Compounds S4

2.2) Reaction of 2c with CO S17

2.3) Thermolysis of 2a S18

2.4) Reaction of 2a with 2-isocyano-1,3-dimethylbenzene S20

2.5) Preparation of ^{13}C Labelled Samples S21

2.6) DOSY Spectrum of 3_2 and 3-THF$_2$ S23

3) Single Crystal X-ray Diffraction Data **S25**

4) DFT Studies **S33**

4.1) Computational methods S33

4.2) Calculated Reaction Pathway S34

4.3) NBO Analysis of Some Reaction Intermediates S36

4.4) QTAIM Analysis S43

5) NMR Spectroscopy **S45**

5.1) NMR Spectra of Isolated complexes S45

5.2) NMR Spectra for Determining the NMR Yields of 4b S51

6) IR Spectroscopy **S53**

6) XYZ Coordinates **S55**

7) References **S105**

1) General experimental

Standard Schlenk line and glovebox techniques were used for all manipulations under an inert atmosphere of dinitrogen or argon unless otherwise stated. NMR scale reactions were performed in J. Young NMR tubes. A MBraun Labmaster glovebox was employed, operating at <0.1 ppm O_2 and <0.1 ppm H_2O.

Instruments: ^{1}H, ^{13}C NMR spectra were recorded on BRUKER 400 MHz or 500 MHz machines, and referenced against $SiMe_4$ (^{1}H, ^{13}C). All peaks are referenced against residual solvent and values are quoted in ppm. Data were processed using the MestReNova software. Where needed, chemical shifts were assigned with the assistance of 2D NMR (COSY, HSQC, HMBC, DEPTQ) spectra. The coupling constants (J) are reported in Hertz (Hz). The following abbreviations are used to define multiplicities: s (singlet), d (doublet), t (triplet), q (quadruplet), hept. (heptet), dd (doublet of doublets), ddd (doublet of doublets of doublets), dt (doublet of triplets), td (triplet of doublets), m (multiplet).

Single crystal X-ray data was obtained on Agilent Diffraction Xcalibur PX Ultra A and Xcalibur 3 E diffractometers, and the structures were refined using the SHELXTL, SHELX-97, and SHELX-2013 program systems.

Infrared spectra were obtained on a Cary630 spectrometer (placed with in an MBraun glovebox) from crystalline solids or benzene thin-films on an ATR cell.

Elemental analyses were performed by Elemental Labs (https://www.elementallab.co.uk/).

Chemicals: Solvents were dried over activated alumina from a solvent purification system (SPS) based upon the Grubbs design and de-gassed before use. Glassware was dried for >6 h prior to use at 120 °C. Benzene-d_6 was de-gassed and stored over 3 Å molecular sieves before use. All reagents were acquired from Sigma Aldrich (Merck), Fluorochem, or VWR and used without further purification unless specified. CO was purchased from BOC Ltd and used as received. $[NN\text{-}(MgH)_2]_2$ (NN = $\{NC(Me)CH(Me)CN\text{-}2,6\text{-}i\text{-}Pr_2C_6H_3\}_2$[S1], $^{Mes}BDIAlCl_2$ (^{Mes}BDI = $\{MesNC(Me)\}_2CH$) [S2], ($^{Dipp}BDIMgH)_2$ (^{Dipp}BDI = $\{DippNC(Me)\}_2CH$, Dipp = 2,6-di-isopropylphenyl) [S3] and isocyanomethane[S13] were prepared by literature procedures.

2) Experimental Methods

2.1 Preparation of Compounds

Preparation of 2a

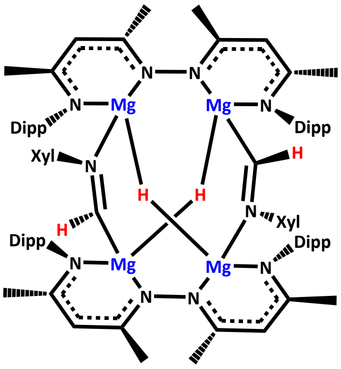

In a glovebox, $[NN\text{-}(MgH)_2]_2$ (40.0 mg, 0.035 mmol, 1 eq) and 2-isocyano-1,3-dimethylbenzene (9.3 mg, 0.07 mmol, 2 eq) were dissolved in C_6D_6 (0.6 mL) and transferred to a J. Young NMR tube. The reaction mixture was kept at 25 °C for 3h. A ^{1}H NMR spectrum was taken at this time point and showed the full conversion of $[NN\text{-}(MgH)_2]_2$ to **2a**. The J. Young NMR tube was returned to the glovebox, the reaction mixture was removed under vacuum and the crude dissolved in 1 mL Et_2O/n-pentane (1:1 v: v) mixture. The solution was filtered into a 4 mL vial and then placed in the glovebox freezer (–35 °C) for 2 days. Bright yellow crystals (**2a**) were successfully obtained. The filtrated crystals were washed with cold n-pentane (3 x 1mL) and then dried in vacuo. **Yield: 27 mg,** 0.021 mmol, 60%.

^{1}H NMR (C_6D_6, 298 K, 400 MHz) δ: 8.83 (s, 2H, C***H***=NXyl), 7.22 – 7.16 (m, 4H, ArC***H***), 7.09 (t, $^3J_{H-H}$ = 7.7 Hz, 2H, p-ArC***H***), 6.96 (t, $^3J_{H-H}$ = 7.7 Hz, 2H, p-ArC***H***), 6.82 (d, $^3J_{H-H}$ = 7.5 Hz, 2H, m-ArC***H***), 6.79 – 6.69 (m, 6H, ArC***H***), 6.52 (d, $^3J_{H-H}$ = 7.4 Hz, 2H, m-ArC***H***), 4.68 (s, 2H, C***H***{C(CH$_3$)}$_2$), 4.53 (s, 2H, C***H***{C(CH$_3$)}$_2$), 3.52 (hept, $^3J_{H-H}$ = 6.7 Hz, 2H, C***H***(CH$_3$)$_2$), 3.51 (hept, $^3J_{H-H}$ = 7.0 Hz, 2H, C***H***(CH$_3$)$_2$), 3.38 (s, 2H, Mg-***H***-Mg), 3.33 (hept, $^3J_{H-H}$ = 6.8 Hz, 2H, C***H***(CH$_3$)$_2$), 2.82 (hept, $^3J_{H-H}$ = 6.8 Hz, 2H, C***H***(CH$_3$)$_2$), 1.88 (s, 6H, 2x NC(C***H$_3$***)), 1.87 (s, 6H, 2x NC(C***H$_3$***)), 1.78 (s, 6H, 2x NC(C***H$_3$***)), 1.77 (s, 6H, Xyl-C***H$_3$***), 1.68 (s, 6H, 2x NC(C***H$_3$***)), 1.55 (d, $^3J_{H-H}$ = 6.7 Hz, 6H, (CH(C***H$_3$***)$_2$), 1.46 (d, $^3J_{H-H}$ = 6.7 Hz, 6H, (CH(C***H$_3$***)$_2$), 1.45 (d, $^3J_{H-H}$ = 6.9 Hz, 6H, (CH(C***H$_3$***)$_2$), 1.41 (d, $^3J_{H-H}$ = 6.9 Hz, 6H, (CH(C***H$_3$***)$_2$), 1.34 (s, 6H, Xyl-C***H$_3$***), 1.04 (d, $^3J_{H-H}$ = 6.9 Hz, 6H, (CH(C***H$_3$***)$_2$), 0.97 (d, $^3J_{H-H}$ = 6.7 Hz, 6H, (CH(C***H$_3$***)$_2$), 0.75 (d, $^3J_{H-H}$ = 6.8 Hz, 6H, (CH(C***H$_3$***)$_2$), 0.42 (d, $^3J_{H-H}$ = 6.9 Hz, 6H, (CH(C***H$_3$***)$_2$).

^{13}C NMR (C_6D_6, 298 K, 101 MHz) δ: 167.4 (2x CH{***C***(CH$_3$)}$_2$), 166.5 (2x CH{***C***(CH$_3$)}$_2$), 165.8 (2x CH{***C***(CH$_3$)}$_2$), 165.2 (2x CH{***C***(CH$_3$)}$_2$), 157.7 (***C***H=NXyl), 146.7 (Ar-***C***), 143.0 (Ar-***C***), 142.8 (Ar-***C***), 142.5 (Ar-***C***), 142.2 (Ar-***C***), 129.4 (Ar-***C***H), 129.0 (Ar-***C***H), 127.5 (Ar-***C***H), 125.2 (Ar-***C***H), 124.7 (Ar-***C***H), 124.4 (Ar-***C***H), 124.2 (Ar-***C***H), 123.7 (Ar-***C***H), 123.3 (Ar-***C***H), 92.1 (2x ***C***H{C(CH$_3$)}$_2$), 92.0 (2x ***C***H{C(CH$_3$)}$_2$), 29.4 (2x ***C***H(CH$_3$)$_2$), 28.0 (2x ***C***H(CH$_3$)$_2$), 27.8 (2x ***C***H(CH$_3$)$_2$), 27.6 (2x ***C***H(CH$_3$)$_2$),

27.1 (2x CH($\boldsymbol{C}H_3)_2$), 26.4 (2x CH($\boldsymbol{C}H_3)_2$), 25.7 (2x CH($\boldsymbol{C}H_3)_2$), 24.8 (2x CH($\boldsymbol{C}H_3)_2$), 24.6 (2x CH($\boldsymbol{C}H_3)_2$), 24.6 (2x CH($\boldsymbol{C}H_3)_2$), 24.5 (2x CH($\boldsymbol{C}H_3)_2$), 23.8 (2x NC($\boldsymbol{C}H_3$)), 23.7 (2x CH($\boldsymbol{C}H_3)_2$), 22.1 (2x NC($\boldsymbol{C}H_3$)), 22.0 (2x NC($\boldsymbol{C}H_3$)), 19.9 (2x NC($\boldsymbol{C}H_3$)), 19.0 (2x Xyl-$\boldsymbol{C}H_3$), 18.7 (2x Xyl-$\boldsymbol{C}H_3$). Some Ar***C*** resonances are overlapping and cannot be observed.

IR (ATR, cm^{-1}): 2993(w), 2959(s), 2923(m), 2884(m), 2866(m), 2659(w), 1810(m), 1531(m), 1509(s), 1477(m), 1456(m), 1435(s), 1376(s), 1357(s), 1311(s), 1276(m), 1251(m), 1234(m).

Anal. Calc. ($C_{80}H_{106}Mg_4N_{10}$): C, 74.36; H, 8.56; N, 10.08. Found: C, 73.15; H, 8.43; N, 10.87.

Preparation of 2b

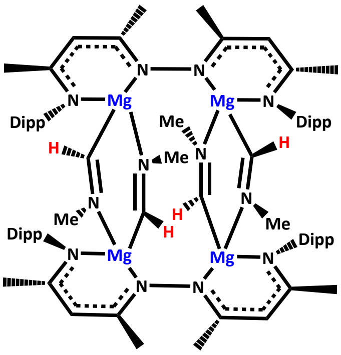

In a glovebox, $[NN-(MgH)_2]_2$ (20.0 mg, 0.018 mmol, 1 eq) and isocyanomethane (2.9 mg, 0.071 mmol, 4 eq) were dissolved in C_6D_6 (0.6 mL) and transferred to a J. Young NMR tube. The reaction mixture was kept at 25 °C for 16 hours. A ^{1}H NMR spectrum was taken at this time point and showed the full conversion of $[NN-(MgH)_2]_2$ to **2b**. Yellow crystals (**2b**) were successfully obtained from C_6D_6 after crystalizing for 3 days at 25 °C. The filtrated crystals were washed with cold n-pentane (3 x 1mL) and then dried in vacuo. **Yield: 12 mg,** 0.009 mmol, 52%.

^{1}H NMR (C_6D_6, 298 K, 400 MHz) δ: 9.46 (s, 2H, C***H***N-CH$_3$), 9.45 (s, 2H, C***H***N-CH$_3$), 7.11 – 7.05 (overlapping signals, 8H, Ar***H***), 6.97 (d, $^3J_{H-H}$ = 7.6 Hz, 4H, Ar***H***), 4.74 (s, 4H, C***H***{C(CH$_3$)}$_2$), 3.52 (hept, $^3J_{H-H}$ = 6.9 Hz, 4H, C***H***(CH$_3$)$_2$), 3.04 (s, 6H, (NC(***C***H$_3$))), 3.03 (s, 6H, (NC(***C***H$_3$))), 2.82 (hept, $^3J_{H-H}$ = 6.9 Hz, 4H, C***H***(CH$_3$)$_2$), 2.18 (s, 12H, 4x NC(C***H$_3$***)), 1.62 (s, 12H, 4x NC(C***H$_3$***)), 1.35 (d, $^3J_{H-H}$ = 7.0 Hz, 12H, (CH(C***H$_3$***)$_2$), 1.20 (d, $^3J_{H-H}$ = 6.9 Hz, 12H, (CH(C***H$_3$***)$_2$), 1.13 (d, $^3J_{H-H}$ = 6.8 Hz, 12H, (CH(C***H$_3$***)$_2$), 0.17 (d, $^3J_{H-H}$ = 6.8 Hz, 12H, (CH(C***H$_3$***)$_2$).

^{13}C NMR (C_6D_6, 298 K, 101 MHz) δ: 238.5 (4x ***C***HN-CH$_3$), 166.5 (4x CH{***C***(CH$_3$)}$_2$), 166.4 (4x CH{***C***(CH$_3$)}$_2$), 146.0 (Ar-***C***), 142.8 (Ar-***C***), 142.0 (Ar-***C***), 124.9 (Ar-***C***H), 124.2 (Ar-***C***H), 123.6 (Ar-***C***H), 92.0 (4x ***C***H{C(CH$_3$)}$_2$), 57.2 (4x N=C-***C***H$_3$), 28.0 (4x ***C***H(CH$_3$)$_2$), 27.1 (4x ***C***H(CH$_3$)$_2$), 24.8 (4x CH(***C***H$_3$)$_2$), 24.4 (4x CH(***C***H$_3$)$_2$), 24.3 (4x CH(***C***H$_3$)$_2$), 24.1 (4x CH(***C***H$_3$)$_2$), 23.8 (4x NC(***C***H$_3$)), 21.4 (4x NC(***C***H$_3$)). Some Ar***C*** resonances are overlapping and cannot be observed.

Anal. Calc. ($C_{76}H_{110}Mg_4N_{12}$): C, 70.82; H, 8.60; N, 13.04. Found: C, 73.26; H, 8.69; N, 11.01. Due to the highly sensitive nature of this compound CHN results do not match the expected values.

(Note: Handling and Hazards of Isocyanomethane: Class A or B gas filters should be used as respiratory protection, depending on the product. Safety gloves made from butyl or fluorinated rubber should be worn (BGR 190) / (GUV-R 190). Isocyanomethane should store over fresh 3 Å molecular sieves at -35 °C under N_2, away from light. Isocyanomethane can be quenched with bleach. Isocyanomethane has no indication of carcinogenicity to humans (not

listed by IARC). Cyanide poisoning is identified by rapid, deep breathing and shortness of breath, general weakness, giddiness, headaches, vertigo, confusion, convulsions/seizures and eventually loss of consciousness.[S14] Antidotes to cyanide poisoning include hydroxocobalamin and sodium nitrite, which release the cyanide from the cytochrome system, and rhodanese, which is an enzyme occurring naturally in mammals that combines serum cyanide with thiosulfate, producing comparatively harmless thiocyanate. Oxygen therapy can also be administered. [S14]

Preparation of 3_2

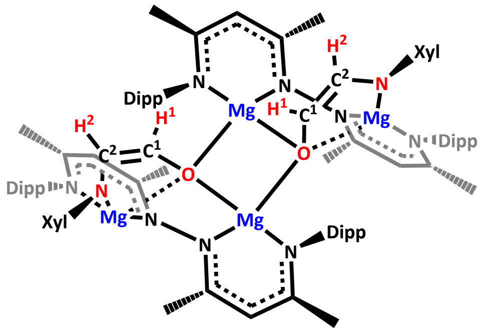

Method One: In a glovebox, [NN-$(MgH)_2$]$_2$ (20.0 mg, 0.018 mmol, 1 eq) and 2-isocyano-1,3-dimethylbenzene (4.7 mg, 0.035 mmol, 2 eq) were dissolved in C_6D_6 (0.6 mL) and transferred to a J. Young NMR tube. The mixture was allowed to react for 1 hour at 25 °C. The reaction mixture was frozen at –78 °C and the NMR tube was evacuated, CO gas (~1.5 bar, ~ 0.135 mmol) was introduced and the reaction mixture was heated at 60 °C for 14 hours. A ^{1}H NMR spectrum was taken at this time point and showed the full conversion of [NN-$(MgH)_2$]$_2$ to **3_2**. The J. Young NMR tube was returned to the glovebox, the solvent was removed under vacuum and the crude product dissolved in 1 mL of a Et_2O/n-pentane (1:1 v: v) mixture. The solution was filtered into a 4 mL vial and then placed in the glovebox freezer (–35 °C) for about 2 days. Bright yellow crystals (**3_2**) were successfully obtained. The filtrated crystals were washed with cold n-pentane (3 x 1mL) and then dried in vacuo. **Yield: 8 mg,** 0.006 mmol, 31%.

Method Two: In a glovebox, **2a** (20.0 mg, 0.014 mmol, 1 eq) was dissolved in C_6D_6 (0.6 mL) and transferred to a J. Young NMR tube. The NMR tube was frozen and evacuated, CO gas (~1.5 bar, ~ 0.135 mmol) was introduced into the NMR tube and the reaction mixture was heated at 60 °C for 14 hours. A ^{1}H NMR spectrum was taken at this time point and showed the full conversion of **2a** to **3_2**. The J. Young NMR tube was returned to the glovebox, the solvent was removed under vacuum and the crude product dissolved in 1 mL a Et_2O/n-pentane (1:1 v: v) mixture. The solution was filtered into a 4 mL vial and then placed in the glovebox freezer (–35 °C) for about 2 days. Bright yellow crystals (**3_2**) were successfully obtained. The filtrated crystals were washed with cold n-pentane (3 x 1mL) and then dried in vacuo. **Yield: 7 mg,** 0.005 mmol, 35%.

^{1}H NMR (C_6D_6, 298 K, 400 MHz) δ: 7.20 – 6.88 (overlapping signals, 18H, Ar***H***), 6.29 (d, $^3J_{H\text{-}H}$ = 2.2 Hz, 2H, OC^{1}H^1=C^2***H***^{2}NXyl), 5.03 (d, $^3J_{H\text{-}H}$ = 2.2 Hz, 2H, OC^1***H***^{1}=C^{2}H^{2}NXyl), 4.74 (s, 2H, C***H***{C(CH$_3$)}$_2$), 4.54 (s, 2H, C***H***{C(CH$_3$)}$_2$), 3.39 (hept, $^3J_{H\text{-}H}$ = 6.8 Hz, 2H, C***H***(CH$_3$)$_2$), 3.35 (hept, $^3J_{H\text{-}H}$ = 6.6 Hz, 2H, C***H***(CH$_3$)$_2$), 3.07 (hept, $^3J_{H\text{-}H}$ = 6.6 Hz, 2H, C***H***(CH$_3$)$_2$), 2.85 (hept, $^3J_{H\text{-}H}$ = 6,8 Hz, 2H, C***H***(CH$_3$)$_2$), 2.24 (s, 6H, Xyl-C***H$_3$***), 2.09 (s, 6H, 2x NC(C***H$_3$***)), 1.81 (s, 6H, 2x NC(C***H$_3$***)), 1.66 (s, 6H, 2x NC(C***H$_3$***)), 1.60 (s, 6H, 2x NC(C***H$_3$***)), 1.44 (d, $^3J_{H\text{-}H}$ = 7.0 Hz, 6H, (CH(C***H$_3$***)$_2$), 1.43 (s, 6H, Xyl-C***H$_3$***), 1.23 (d, $^3J_{H\text{-}H}$ = 6.7 Hz, 6H, (CH(C***H$_3$***)$_2$), 1.22 (d, $^3J_{H\text{-}H}$ = 6.7 Hz, 6H, (CH(C***H$_3$***)$_2$), 1.21 (d, $^3J_{H\text{-}H}$

= 6.8 Hz, 6H, (CH(C***H₃***)₂), 1.04 (d, $^3J_{H\text{-}H}$ = 6.8 Hz, 6H, (CH(C***H₃***)₂), 0.92 (d, $^3J_{H\text{-}H}$ = 6.7 Hz, 6H, (CH(C***H₃***)₂), 0.46 (d, $^3J_{H\text{-}H}$ = 6.9 Hz, 6H, (CH(C***H₃***)₂), 0.40 (d, $^3J_{H\text{-}H}$ = 6.7 Hz, 6H, (CH(C***H₃***)₂)

^{13}C NMR (C_6D_6, 298 K, 101 MHz) δ: 168.6 (2x CH{***C***(CH₃)}₂), 167.9 (2x CH{***C***(CH₃)}₂), 167.3 (2x CH{***C***(CH₃)}₂), 166.9 (2x CH{***C***(CH₃)}₂), 152.5 (Ar-***C***), 152.2 (OC¹H¹=***C²***H²NXyl), 147.1 (Ar-***C***), 146.1 (Ar-***C***), 143.4 (Ar-***C***), 143.0 (Ar-***C***), 142.4 (Ar-***C***), 142.3 (Ar-***C***), 134.2 (Ar-***C***), 134.1 (Ar-***C***), 125.6 (Ar-***C***H), 125.5 (Ar-***C***H), 124.6 (Ar-***C***H), 124.4 (Ar-***C***H), 124.2 (Ar-***C***H), 123.6 (Ar-***C***H), 123.0 (Ar-***C***H), 102.9 (O***C¹***H¹=C²H²NXyl), 93.5 (2x ***C***H{C(CH₃)}₂), 92.4 (2x ***C***H{C(CH₃)}₂), 29.2 (2x ***C***H(CH₃)₂), 28.4 (2x ***C***H(CH₃)₂), 27.9 (2x ***C***H(CH₃)₂), 27.6 (2x ***C***H(CH₃)₂), 26.6 (2x CH(***C***H₃)₂), 25.5 (2x CH(***C***H₃)₂), 25.4 (2x CH(***C***H₃)₂), 25.3 (2x CH(***C***H₃)₂), 24.9 (2x CH(***C***H₃)₂), 24.4 (2x NC(***C***H₃)), 24.3 (2x NC(***C***H₃)), 24.2 (2x CH(***C***H₃)₂), 22.6 (2x CH(***C***H₃)₂), 22.0 (2x CH(***C***H₃)₂), 20.5 (2x NC(***C***H₃)), 19.8 (2x NC(***C***H₃)), 19.7 (2x Xyl-***C***H₃), 17.80 (2x Xyl-***C***H₃). Some Ar***C*** resonances are overlapping and cannot be observed.

IR (ATR, cm^{-1}): 2959(s), 2925(s), 2886(s), 2866(s), 1957(m), 1812(m), 1601(m), 1519(m), 1477(s), 1458(w), 1435(m), 1415(w), 1384(m), 1358(w), 1314(m), 1254(m), 1231(w).

Anal. Calc. ($C_{96}H_{138}Mg_4N_{10}O_4$): C, 72.36; H, 8.73; N, 8.79. Found: C, 72.39; H, 8.22; N, 8.43. The CHN results match the expected values for **3_2-(OEt_2)$_2$**.

^{1}H NMR data showing the time course of the reaction of **2a** with CO

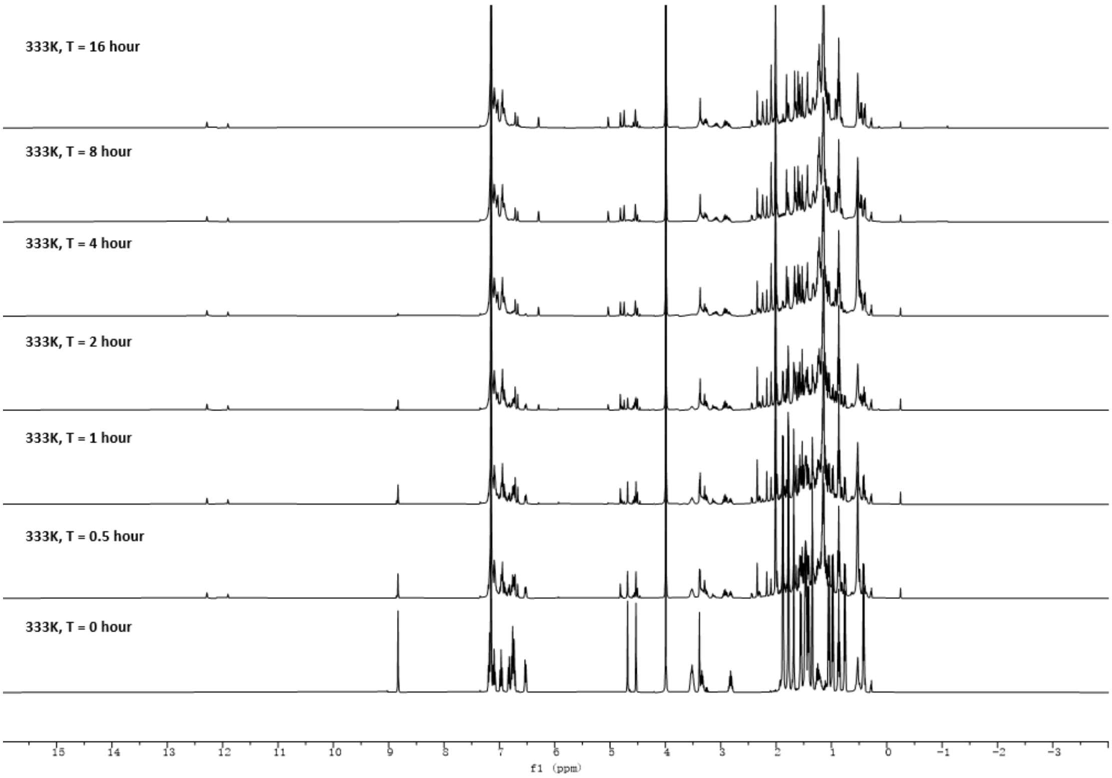

Figure S1. Reaction of **2a** with CO followed by ^{1}H NMR spectroscopy

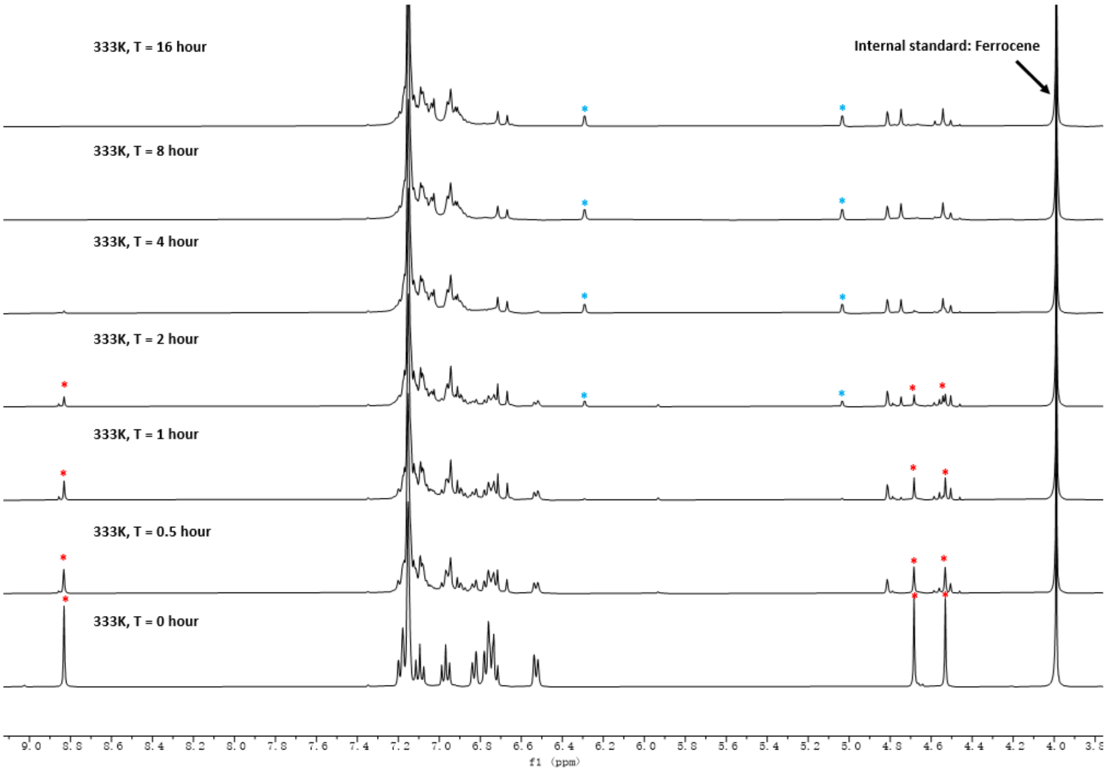

Figure S2. NMR spectra from kinetic experiments. (^{1}H NMR (C_6D_6, 400 MHz): ("*" is the peak of **3_2**, "*" is the peak of **2a**).

Preparation of 3_2-^{13}C

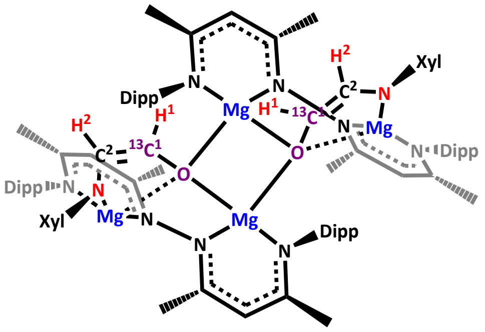

In a glovebox, **2a** (20.0 mg, 0.014 mmol, 1 eq) was dissolved in C_6D_6 (0.6 mL) and transferred to a J. Young NMR tube. The reaction mixture was frozen at –78 °C and the NMR tube was evacuated, ^{13}CO gas (~1.5 bar, about 0.135 mmol, 3.78 mg) was introduced into the NMR tube and the reaction mixture was heated at 60 °C for 14 hours. A ^{1}H NMR spectrum was taken at this time point and showed the full conversion of **2a** to **3_2-^{13}C**. ^{1}H NMR, ^{13}C NMR and some 2D (COSY, HSQC, HMBC, DEPTQ) spectra were taken at this time point and showed the full conversion of **2** to **3_2-^{13}C**.

^{1}H NMR (C_6D_6, 298 K, 400 MHz) δ: 7.20 – 6.88 (overlapping signals, 18H, Ar***H***), 6.29 (d, $^{3}J_{H-H}$ = 13.9, 2.2 Hz, 2H, O^{13}C^{1}H^{1}=C^{2}***H***^{2}NXyl), 5.03 (d, $^{3}J_{H-H}$ = 174.8, 2.2 Hz, 2H, O^{13}C^{1}***H***^{1}=C^{2}H^{2}NXyl), 4.74 (s, 2H, C***H***{C(CH$_3$)}$_2$), 4.54 (s, 2H, C***H***{C(CH$_3$)}$_2$), 3.39 (hept, $^{3}J_{H-H}$ = 6.8 Hz, 2H, C***H***(CH$_3$)$_2$), 3.35 (hept, $^{3}J_{H-H}$ = 6.6 Hz, 2H, C***H***(CH$_3$)$_2$), 3.07 (hept, $^{3}J_{H-H}$ = 6.6 Hz, 2H, C***H***(CH$_3$)$_2$), 2.85 (hept, $^{3}J_{H-H}$ = 6,8 Hz, 2H, C***H***(CH$_3$)$_2$), 2.24 (s, 6H, Xyl-C***H$_3$***), 2.09 (s, 6H, 2x NC(C***H$_3$***)), 1.81 (s, 6H, 2x NC(C***H$_3$***)), 1.66 (s, 6H, 2x NC(C***H$_3$***)), 1.60 (s, 6H, 2x NC(C***H$_3$***)), 1.44 (d, $^{3}J_{H-H}$ = 7.0 Hz, 6H, (CH(C***H$_3$***)$_2$), 1.43 (s, 6H, Xyl-C***H$_3$***), 1.23 (d, $^{3}J_{H-H}$ = 6.7 Hz, 6H, (CH(C***H$_3$***)$_2$), 1.22 (d, $^{3}J_{H-H}$ = 6.7 Hz, 6H, (CH(C***H$_3$***)$_2$), 1.21 (d, $^{3}J_{H-H}$ = 6.8 Hz, 6H, (CH(C***H$_3$***)$_2$), 1.04 (d, $^{3}J_{H-H}$ = 6.8 Hz, 6H, (CH(C***H$_3$***)$_2$), 0.92 (d, $^{3}J_{H-H}$ = 6.7 Hz, 6H, (CH(C***H$_3$***)$_2$), 0.46 (d, $^{3}J_{H-H}$ = 6.9 Hz, 6H, (CH(C***H$_3$***)$_2$), 0.40 (d, $^{3}J_{H-H}$ = 6.7 Hz, 6H, (CH(C***H$_3$***)$_2$)

^{13}C NMR (C_6D_6, 298 K, 101 MHz) δ: 168.6 (2x CH{***C***(CH$_3$)}$_2$), 167.9 (2x CH{***C***(CH$_3$)}$_2$), 167.3 (2x CH{***C***(CH$_3$)}$_2$), 166.9 (2x CH{***C***(CH$_3$)}$_2$), 152.5 (Ar-***C***), 152.2 (O^{13}C^{1}H^{1}=C^{2}H^{2}NXyl), 147.1 (Ar-***C***), 146.1 (Ar-***C***), 143.4 (Ar-***C***), 143.0 (Ar-***C***), 142.4 (Ar-***C***), 142.3 (Ar-***C***), 134.2 (Ar-***C***), 134.1 (Ar-***C***), 125.6 (Ar-***C***H), 125.5 (Ar-***C***H), 124.6 (Ar-***C***H), 124.4 (Ar-***C***H), 124.2 (Ar-***C***H), 123.6 (Ar-***C***H), 123.0 (Ar-***C***H), 102.9 (O***^{13}C^{1}***H^{1}=C^{2}H^{2}NXyl), 93.5 (2x ***C***H{C(CH$_3$)}$_2$), 92.4 (2x ***C***H{C(CH$_3$)}$_2$), 29.2 (2x ***C***H(CH$_3$)$_2$), 28.4 (2x ***C***H(CH$_3$)$_2$), 27.9 (2x ***C***H(CH$_3$)$_2$), 27.6 (2x ***C***H(CH$_3$)$_2$), 26.6 (2x CH(***C***H$_3$)$_2$), 25.5 (2x CH(***C***H$_3$)$_2$), 25.4 (2x CH(***C***H$_3$)$_2$), 25.3 (2x CH(***C***H$_3$)$_2$), 24.9 (2x CH(***C***H$_3$)$_2$), 24.4 (2x NC(***C***H$_3$)), 24.3 (2x NC(***C***H$_3$)), 24.2 (2x CH(***C***H$_3$)$_2$), 22.6 (2x CH(***C***H$_3$)$_2$), 22.0 (2x CH(***C***H$_3$)$_2$), 20.5 (2x NC(***C***H$_3$)), 19.8 (2x NC(***C***H$_3$)), 19.7 (2x Xyl-***C***H$_3$), 17.80 (2x Xyl-***C***H$_3$). Some Ar***C*** resonances are overlapping and cannot be observed.

Preparation of 3-THF$_2$

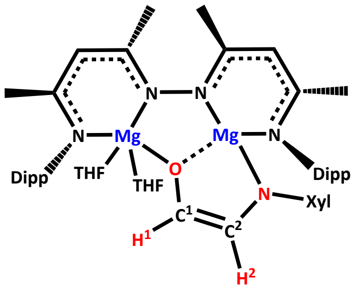

In a glovebox, **3$_2$** (40.0 mg, 0.028 mmol, 1 eq) was dissolved in d$_8$-THF (0.8 mL) and transferred to a J. Young NMR tube. The reaction mixture was stored at 25 °C for 1 hour. A ^{1}H NMR spectrum was taken at this time point and showed the full conversion of **3$_2$** to **3-THF$_2$**. The J. Young NMR tube was returned to the glovebox, the solvent was removed under vacuum and the crude dissolved in 1 mL of a THF/n-pentane (1:2 v: v) mixture. The solution was filtered into a 4 mL vial and then placed in the glovebox freezer (–35 °C) for about 3 days. Bright yellow crystals (**3-THF$_2$**) were successfully obtained. The filtrated crystals were washed with cold n-pentane (3 x 1mL) and then dried in vacuo. **Yield: 14 mg,** 0.017 mmol, 60%.

^{1}H NMR (d$_8$-THF, 298 K, 400 MHz) δ: 7.12 – 7.03 (m, 3H, Ar-***H***), 6.99 (d, $^3J_{H-H}$ = 7.5 Hz, 1H, Ar-***H***), 6.92 (m, 1H, Ar-***H***), 6.79 (d, $^3J_{H-H}$ = 7.5 Hz, 1H, Ar-***H***), 6.60 (d, $^3J_{H-H}$ = 7.3 Hz, 2H, NXyl-***H***), 6.28 (t, $^3J_{H-H}$ = 7.3 Hz, 1H, NXyl-***H***), 5.42 (d, $^3J_{H-H}$ = 3.4 Hz, 1H, OC^{1}H^1=C^2***H^2***NXyl), 5.32 (d, $^3J_{H-H}$ = 3.3 Hz, 1H, OC^1***H^1***=C^{2}H^{2}NXyl), 4.52 (s, 1H), 3.35 (hept, $^3J_{H-H}$ = 6.6 Hz, 1H, C***H***(CH$_3$)$_2$), 3.30 (hept, $^3J_{H-H}$ = 6.6 Hz, 1H, C***H***(CH$_3$)$_2$), 3.18 (hept, $^3J_{H-H}$ = 6.5 Hz, 1H, C***H***(CH$_3$)$_2$), 2.70 (hept, $^3J_{H-H}$ = 6.5 Hz, 1H, C***H***(CH$_3$)$_2$), 1.96 (s, 3H, Xyl-C***H$_3$***), 1.91 (s, 3H, Xyl-C***H$_3$***), 1.54 (d, $^3J_{H-H}$ = 7.0 Hz, 6H, (CH(C***H$_3$***)$_2$), 1.23 (d, $^3J_{H-H}$ = 6.7 Hz, 6H, NC(C***H$_3$***)), 1.20 (d, $^3J_{H-H}$ = 6.7 Hz, 6H, (CH(C***H$_3$***)$_2$), 1.11 (d, $^3J_{H-H}$ = 6.8 Hz, 6H, (CH(C***H$_3$***)$_2$), 1.02 (d, $^3J_{H-H}$ = 6.7 Hz, 6H, (CH(C***H$_3$***)$_2$), 0.96 (d, $^3J_{H-H}$ = 6.8 Hz, 6H, (CH(C***H$_3$***)$_2$).

^{13}C NMR (d$_8$-THF, 298 K, 101 MHz) δ: 165.6 (CH{***C***(CH$_3$)}$_2$), 165.2 (CH{***C***(CH$_3$)}$_2$), 164.9 (CH{***C***(CH$_3$)}$_2$), 164.6 (CH{***C***(CH$_3$)}$_2$), 155.9 (OC^{1}H^1=***C^2***H^{2}NXyl), 149.5 (Ar-***C***), 143.7 (Ar-***C***), 142.9 (Ar-***C***), 142.5 (Ar-***C***), 141.1 (Ar-***C***), 131.1 (Ar-***C***), 128.8 (Ar-***C***), 127.1 (Ar-***C***H), 124.2 (Ar-***C***H), 123.8 (Ar-***C***H), 123.4 (Ar-***C***H), 123.1 (Ar-***C***H), 123.0 (Ar-***C***H), 122.4 (Ar-***C***H), 118.5 (Ar-***C***H), 116.8 (O***C^1***H^1=C^{2}H^{2}NXyl), 90.6 (***C***H{C(CH$_3$)}$_2$), 89.9 (***C***H{C(CH$_3$)}$_2$), 28.1 (***C***H(CH$_3$)$_2$), 28.0 (***C***H(CH$_3$)$_2$), 27.9 (***C***H(CH$_3$)$_2$), 27.2 (***C***H(CH$_3$)$_2$), 23.3 (2x NC(***C***H$_3$)), 23.2 (2x NC(***C***H$_3$)), 22.2 (2x CH(***C***H$_3$)$_2$), 19.4 (2x CH(***C***H$_3$)$_2$), 19.2 (Xyl-***C***H$_3$). Some Ar***C*** resonances are overlapping and cannot be observed.

Preparation of 2c

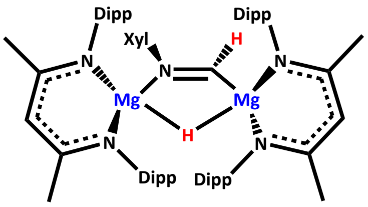

In a glovebox, (^{Dipp}BDIMgH)$_2$ (20.0 mg, 0.023 mmol, 1 eq) and 2-isocyano-1,3-dimethylbenzene (3.0 mg, 0.023 mmol, 1 eq) were dissolved in C_6D_6 (0.6 mL) and transferred to a J. Young NMR tube. The reaction mixture was heated at 60 °C for 14 hours. A ^{1}H NMR spectrum was taken at this time point and showed the full conversion of (^{Dipp}BDIMgH)$_2$ to **2c**. The J. Young NMR tube was returned to the glovebox, the solvent was removed under vacuum and the crude dissolved in 1 mL of a Et_2O/n-pentane (1:1 v: v) mixture. The solution was filtered into a 4 mL vial and then placed in the glovebox freezer (–35 °C) for about 2 days. Colourless crystals (**5**) were successfully obtained. The filtrated crystals were washed with cold n-pentane (3 x 1mL) and then dried in vacuo. **Yield: 9 mg,** 0.009 mmol, 40%.

^{1}H NMR (C_6D_6, 298 K, 400 MHz) δ: 9.07 (d, $^3J_{H-H}$ = 2.3 Hz, 1H, C***H***=NXyl), 7.16 – 7.11 (m, 4H, Ar***H***), 7.28 (t, $^3J_{H-H}$ = 6.8 Hz, 1H, Ar***H***), 7.23 (t, $^3J_{H-H}$ = 6.8 Hz, 1H, Ar***H***), 7.05 (t, $^3J_{H-H}$ = 7.4 Hz, 1H, Ar***H***), 6.99 (d, $^3J_{H-H}$ = 7.4 Hz, 1H, Ar***H***), 6.92 (dd, $^3J_{H-H}$ = 7.4 , 1.9 Hz, 1H, Ar***H***), 6.88 (dd, $^3J_{H-H}$ = 7.4 , 1.9 Hz, 1H, Ar***H***), 6.73 (t, $^3J_{H-H}$ = 7.4 Hz, 1H, Ar***H***), 6.70 (t, $^3J_{H-H}$ = 7.4 Hz, 1H, Ar***H***), 6.64 – 6.54 (m, 3H, Ar***H***), 4.86 (s, 1H, C***H***{C(CH$_3$)}$_2$), 4.80 (s, 1H, C***H***{C(CH$_3$)}$_2$), 3.81 (hept, $^3J_{H-H}$ = 6.8 Hz, 1H, C***H***(CH$_3$)$_2$), 3.69 (hept, $^3J_{H-H}$ = 6.8 Hz, 1H, C***H***(CH$_3$)$_2$), 3.67 (hept, $^3J_{H-H}$ = 6.8 Hz, 1H, C***H***(CH$_3$)$_2$), 3.54 (d, $^3J_{H-H}$ = 2.3 Hz, 1H, Mg-***H***-Mg), 3.39 (hept, $^3J_{H-H}$ = 6.9 Hz, 1H, C***H***(CH$_3$)$_2$), 3.13 (hept, $^3J_{H-H}$ = 6.8 Hz, 1H, C***H***(CH$_3$)$_2$), 3.00 (hept, $^3J_{H-H}$ = 6.7 Hz, 1H, C***H***(CH$_3$)$_2$), 2.91 (hept, $^3J_{H-H}$ = 6.9 Hz, 1H, C***H***(CH$_3$)$_2$), 2.85 (hept, $^3J_{H-H}$ = 6.9 Hz, 1H, C***H***(CH$_3$)$_2$), 1.67 (s, 6H, NC(C***H$_3$***)), 1.65 (d, $^3J_{H-H}$ = 6.8 Hz, 3H, CH(C***H$_3$***)(CH$_3$)), 1.56 (s, 3H, NC(C***H$_3$***)), 1.54 (d, $^3J_{H-H}$ = 6.8 Hz, 3H, CH(C***H$_3$***)(CH$_3$), 1.45 (s, 3H, NC(C***H$_3$***)), 1.42 (d, $^3J_{H-H}$ = 6.9 Hz, 3H, CH(C***H$_3$***)(CH$_3$)) 1.40 (d, $^3J_{H-H}$ = 6.9 Hz, 3H, CH(C***H$_3$***)(CH$_3$)), 1.27 (d, $^3J_{H-H}$ = 6.7 Hz, 3H, CH(C***H$_3$***)(CH$_3$)), 1.27 (d, $^3J_{H-H}$ = 6.7 Hz, 6H, CH(C***H$_3$***)(CH$_3$)), 1.23 (d, $^3J_{H-H}$ = 6.7 Hz, 6H, CH(C***H$_3$***)(CH$_3$)), 1.18 (s, 3H, NXyl-C***H$_3$***), 1.15 (d, $^3J_{H-H}$ = 6.9 Hz, 3H, CH(C***H$_3$***)(CH$_3$)), 1.06 (d, $^3J_{H-H}$ = 6.8 Hz, 3H, CH(C***H$_3$***)(CH$_3$)), 1.05 (d, $^3J_{H-H}$ = 6.8 Hz, 3H, CH(C***H$_3$***)(CH$_3$)), 0.97 (d, $^3J_{H-H}$ = 6.8 Hz, 3H, CH(C***H$_3$***)(CH$_3$)), 0.87 (s, 3H, NXyl-C***H$_3$***), 0.64 (d, $^3J_{H-H}$ = 6.7 Hz, 3H, CH(C***H$_3$***)(CH$_3$)), 0.37 (d, $^3J_{H-H}$ = 6.8 Hz, 3H, CH(C***H$_3$***)(CH$_3$)), 0.34 (d, $^3J_{H-H}$ = 6.8 Hz, 3H, CH(C***H$_3$***)(CH$_3$)).

^{13}C NMR (C_6D_6, 298 K, 101 MHz) δ: 169.0 (CH{***C***(CH$_3$)}$_2$), 168.9 (CH{***C***(CH$_3$)}$_2$), 168.6 (CH{***C***(CH$_3$)}$_2$), 168.1 (CH{***C***(CH$_3$)}$_2$), 157.1 (***C***H=NXyl), 147.0 (Ar-***C***), 146.8 (Ar-***C***), 146.7 (Ar-***C***), 144.1 (Ar-***C***), 143.6 (Ar-***C***), 143.2 (Ar-***C***), 142.9 (Ar-***C***), 142.6 (Ar-***C***), 142.3 (Ar-***C***), 141.9 (Ar-***C***), 141.1 (Ar-***C***), 129.2 (Ar-***C***H), 128.6 (Ar-***C***H), 127.0 (Ar-***C***H), 125.6 (Ar-***C***H), 125.5 (Ar-***C***H), 125.2 (Ar-***C***H), 124.9 (Ar-***C***H), 124.5 (Ar-***C***H), 124.4 (Ar-***C***H), 124.2 (Ar-***C***H), 124.1 (Ar-***C***H), 123.6 (Ar-***C***H), 123.5 (Ar-***C***H), 123.4 (Ar-***C***H), 123.2 (Ar-***C***H), 96.0 (***C***H{C(CH$_3$)}$_2$), 95.0 (***C***H{C(CH$_3$)}$_2$), 30.6

(**C**H(CH_3)(CH_3)), 29.5 (CH(**C**H_3)(CH_3)), 29.1 (**C**H(CH_3)(CH_3)), 28.2 (**C**H(CH_3)(CH_3)), 28.1 (**C**H(CH_3)(CH_3)), 27.8 (**C**H(CH_3)(CH_3)), 27.5 (CH(**C**H_3)(CH_3)), 27.1 (**C**H(CH_3)(CH_3)), 27.0 (**C**H(CH_3)(CH_3)), 26.7 (**C**H(CH_3)(CH_3)), 25.6 (CH(**C**H_3)(CH_3)), 25.2 (CH(**C**H_3)(CH_3)), 25.1 (CH(**C**H_3)(CH_3)), 25.1 (CH(**C**H_3)(CH_3)), 25.0 (CH(**C**H_3)(CH_3)), 24.9 (CH(**C**H_3)(CH_3)), 24.8 (NC(**C**H_3)), 24.7 (2x NC(**C**H_3)), 24.5 (NC(**C**H_3)), 24.4 (CH(**C**H_3)(CH_3)), 24.3 (CH(**C**H_3)(CH_3)), 24.2 (CH(**C**H_3)(CH_3)), 22.9 (CH(**C**H_3)(CH_3)), 22.8 (CH(**C**H_3)(CH_3)), 22.7 (CH(**C**H_3)(CH_3)), 22.5 (CH(**C**H_3)(CH_3)), 22.2 (CH(**C**H_3)(CH_3)), 21.2 (NXyl-**C**H_3), 17.0 (NXyl-**C**H_3). Some Ar**C** resonances are overlapping and cannot be observed.

Anal. Calc. ($C_{67}H_{93}Mg_2N_5$): C, 79.12; H, 9.22; N, 6.89. Found: C, 79.71; H, 9.26; N, 7.32.

Preparation of 4a

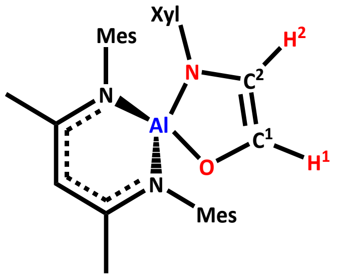

In a glovebox, $\mathbf{3_2}$ (40.0 mg, 0.028 mmol, 1 eq) and ^{Mes}BDIAlCl$_2$ (23.9 mg, 0.056 mmol, 2 eq) were dissolved in C_6D_6 (1.0 mL) and transferred to a J. Young NMR tube. The reaction mixture was heated to 100 °C for 48 hours. A ^{1}H NMR spectrum was taken at this time point and showed the full conversion of $\mathbf{3_2}$ to **4a**. The J. Young NMR tube was returned to the glovebox, the reaction mixture was decanted into a vial, the solvent was removed under vacuum and the crude mixture dissolved in 1 mL of a Et_2O/n-pentane (1:1 v: v) mixture. The solution was filtered into a 4 mL vial and then placed in the glovebox freezer (–35 °C) for 3 days. Bright red crystals (**4a**) were successfully obtained. The filtrated crystals were washed with cold n-pentane (3 x 1mL) and then dried in vacuo. **Yield: 8 mg,** 0.008 mmol, 60%.

^{1}H NMR (C_6D_6, 298 K, 400 MHz) δ: 6.96 (dd, $^3J_{H-H}$ = 7.4, 0.7 Hz, 2H, NXyl-***H***), 6.89 (dd, $^3J_{H-H}$ = 6.5, 1.4Hz, 1H, NXyl-***H***), 6.74 (d, $^3J_{H-H}$ = 7.0 Hz, 2H, NMes-***H***), 6.64 (d, $^3J_{H-H}$ = 7.0 Hz, 2H, NMes-***H***), 6.41 (d, $^3J_{H-H}$ = 2.7 Hz, 1H, OC^{1}H^1=C^2***H^2***NXyl), 5.69 (d, $^3J_{H-H}$ = 2.7 Hz, 1H, OC^1***H^1***=C^{2}H^{2}NXyl), 5.00 (s, 1H, C***H***{C(CH$_3$)}$_2$), 2.46 (s, 6H, NMes-C***H$_3$***), 2.10 (s, 6H, NMes-C***H$_3$***), 1.88 (s, 6H, NXyl-C***H$_3$***), 1.81 (s, 6H, NMes-C***H$_3$***), 1.43 (s, 6H, (NC(C***H$_3$***))).

^{13}C NMR (C_6D_6, 298 K, 101 MHz) δ: 171.2 (N***C***(CH$_3$)), 148.7 (Ar-***C***), 140.4 (Ar-***C***), 136.0 (Ar-***C***), 134.2 (Ar-***C***), 134.2 (Ar-***C***), 130.1 (Ar-***C***H), 129.6 (Ar-***C***H), 128.4 (OC^{1}H^1=***C^2***H^{2}NXyl), 123.9 (O***C^1***H^1=C^{2}H^{2}NXyl), 121.8 (Ar-***C***H), 99.1 (***C***H{C(CH$_3$)}$_2$), 22.6 (NC(***C***H$_3$)), 20.8 (NMes-***C***H$_3$), 19.4 (NXyl-***C***H$_3$), 18.9 (NMes-***C***H$_3$), 18.3 (NMes-***C***H$_3$). Some Ar***C***H resonances are overlapping and cannot be observed.

IR (ATR, cm^{-1}): 2953(s), 2916(s), 2856(s), 2732(s), 1608(w), 1584(w), 1530(s), 1478(w), 1466(m), 1438(w), 1371(s), 1342(w), 1297(w), 1273(s), 1239(s).

Anal. Calc. ($C_{31}H_{38}AlN_3O$): C, 75.98; H, 7.73; N, 8.05. Found: C, 69.09; H, 7.37; N, 7.03. Due to the highly sensitive nature of this compound CHN results do not match the expected values.

Preparation of 4b

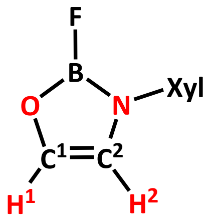

In a glovebox, **4a** (40.0 mg, 0.066 mmol, 1 eq) and BF_3-Et_2O (9.8 mg, 0.069 mmol, 1.05 eq) were dissolved in C_6D_6 (1.0 mL) and transferred to a J. Young NMR tube with ferrocene as the internal standard. The reaction mixture was stored at 25 °C for 2 hours. Quantitative ^{1}H NMR spectrum spectroscopy showed the full conversion of **4a** to **4b**. **NMR Yield:** 63%.

^{1}H NMR (C_6D_6, 298 K, 400 MHz) δ: 6.91 (dd, $^3J_{H\text{-}H}$ = 8.4, 6.5 Hz, 1H, NXyl-***H***), 6.84 (d, $^3J_{H\text{-}H}$ = 7.2 Hz, 2H, NXyl-***H***), 6.32 (bs, 1H, OC^1H^1=$C^2\boldsymbol{H^2}$NXyl), 5.67 (bs, 1H, $OC^1\boldsymbol{H^1}$=C^2H^2NXyl), 1.94 (s, 6H, NXyl-C$\boldsymbol{H_3}$).

^{13}C NMR (C_6D_6, 298 K, 101 MHz) δ: 136.2 (Ar-***C***), 129.8 (Ar-***C***H), 129.6 (Ar-***C***H), 128.5 (OC^1H^1=$\boldsymbol{C^2}H^2$NXyl), 128.3 (Ar-***C***), 121.0 ($O\boldsymbol{C^1}H^1$=C^2H^2NXyl), 20.5 (NXyl-***C***H_3).

^{11}B NMR (C_6D_6, 298 K, 128.0 MHz) δ 21.41 (s)

^{19}F NMR (C_6D_6, 298 K, 376.5 MHz) δ -156.50 (d, $J_{B\text{-}F}$ = 28.8 Hz).

2.2 Reaction of 2c with CO

In a glovebox, **2c** (9.0 mg, 0.009 mmol, 1 eq) was dissolved in C_6D_6 (0.6 mL) and transferred to a J. Young NMR tube. The NMR tube was frozen at –78 °C and evacuated, CO gas (~1.5 bar, about 0.135 mmol, 3.78 mg) was introduced into the NMR tube and the reaction mixture was kept at 25 °C for 14 hours. A ^1H NMR spectrum was taken at this time point and showed no reaction. The reaction was then heated to 60 °C for 30 hours. A ^1H NMR spectrum was taken at this time point and showed all **2c** was consumed, forming a complex mixture of products with no evidence for formation of an ethene amidolate.

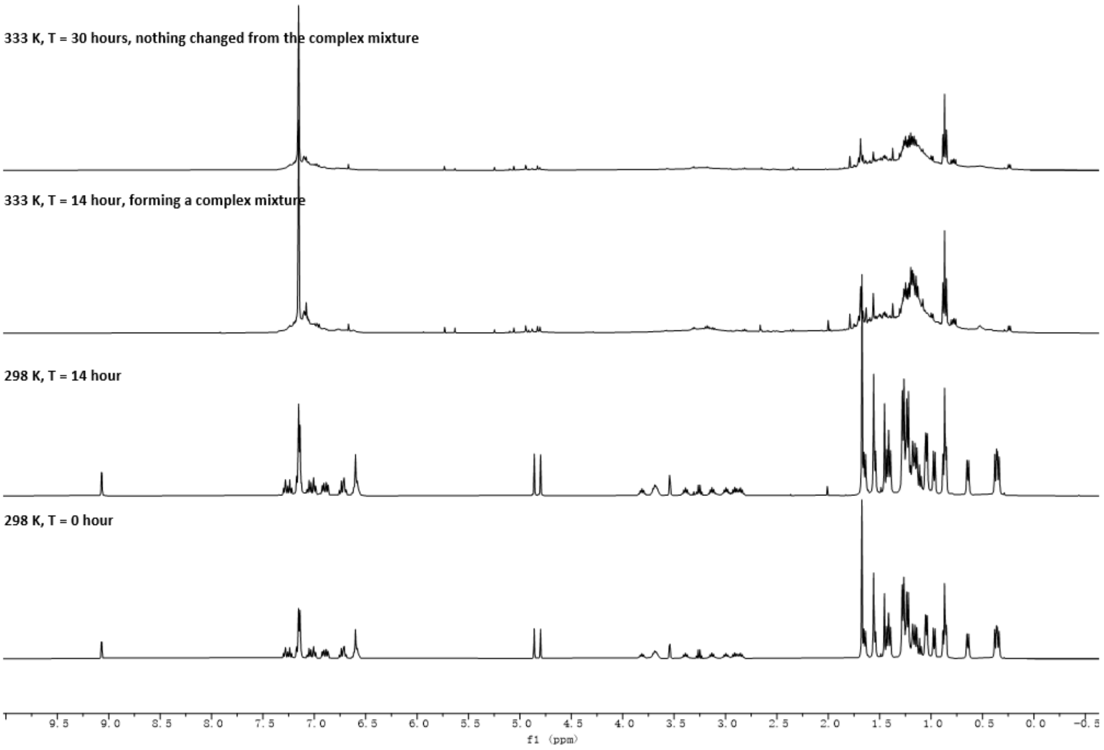

Figure S3. Reaction of **2c** with CO followed by ^1H NMR spectroscopy

2.3 Thermolysis of 2a in absence of CO

In a glovebox, **2a** (5.0 mg, 0.004 mmol) was dissolved in C_6D_6 (0.5 mL) and transferred to a J. Young NMR tube. The reaction mixture was heated to 60 °C for 3 hours and then up to 80 °C for 15 hours. The reaction was monitored by ^{1}H NMR spectroscopy. **2a** was slowly consumed, forming a complex mixture of products. No clear evidence can prove the formation process of azamethylene intermediate.

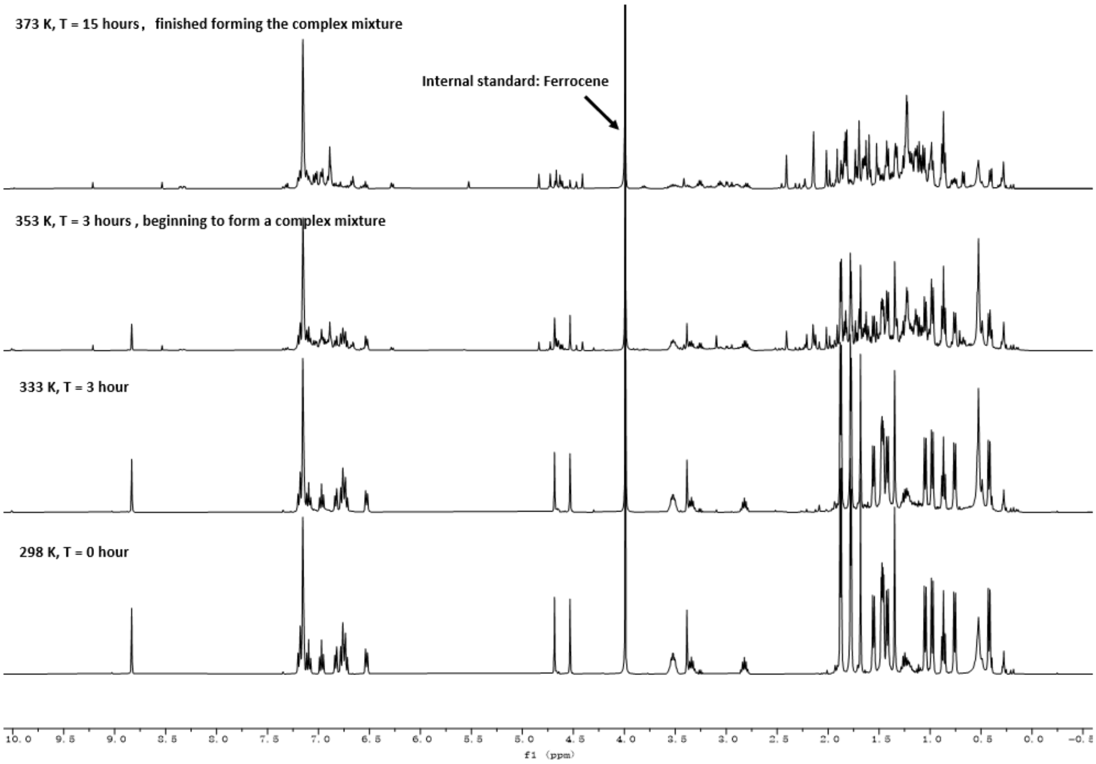

Figure S4. Thermolysis of **2a** followed by ^{1}H NMR spectroscopy

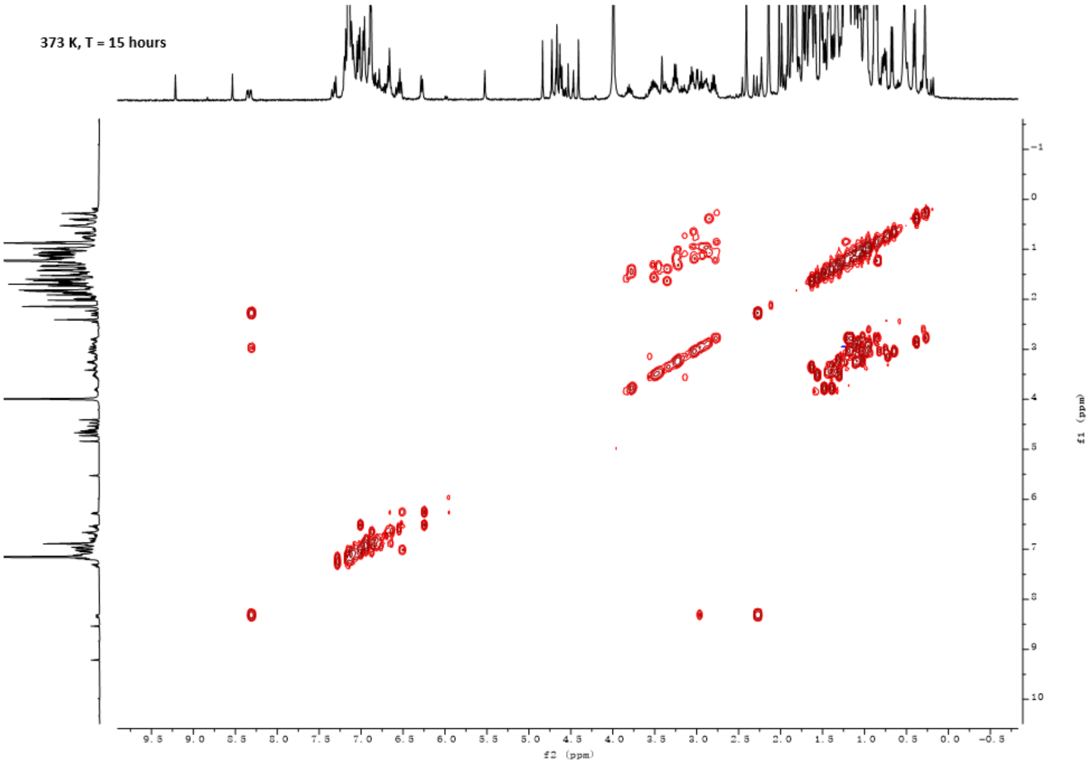

Figure S5. COSY NMR of **2a** after heating to 80 °C for 15 hours

2.4 Attempted Reaction of 2a with 2-isocyano-1,3-dimethylbenzene

In a glovebox, **2a** (10.0 mg, 0.008 mmol, 1 eq) and 2-isocyano-1,3-dimethylbenzene (2.0 mg, 0.016 mmol, 2 eq) were dissolved in C_6D_6 (0.6 mL) and transferred to a J. Young NMR tube. The reaction mixture was heated to 60 °C for 24 hours and then up to 100 °C for 12 hours. A ^{1}H NMR spectrum was taken at this time point and showed that all **2a** was consumed, forming a complex mixture of products with no evidence for formation of the ethene diamidolate.

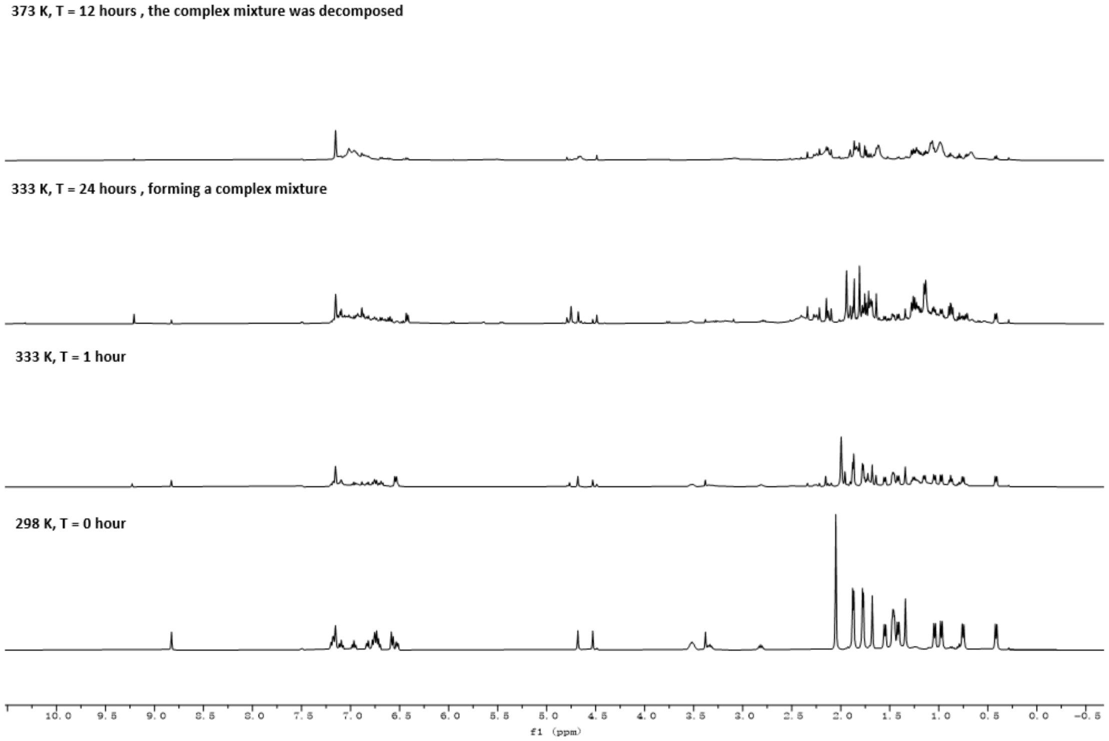

Figure S6. ^{1}H NMR of reaction of **2a** with 2-isocyano-1,3-dimethylbenzene

2.5 Preparation of ^{13}C Labelled Samples

In order to unequivocally assign chemical shifts for the carbon environments, a ^{13}C labelled sample of **3_2-^{13}C** was synthesized.

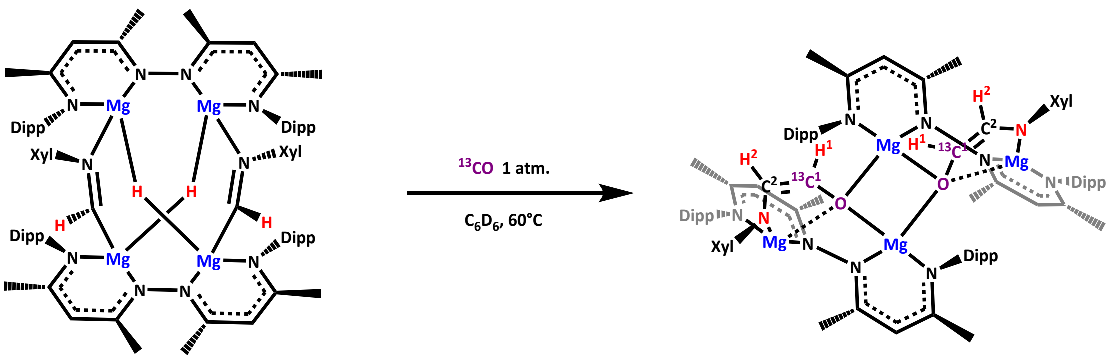

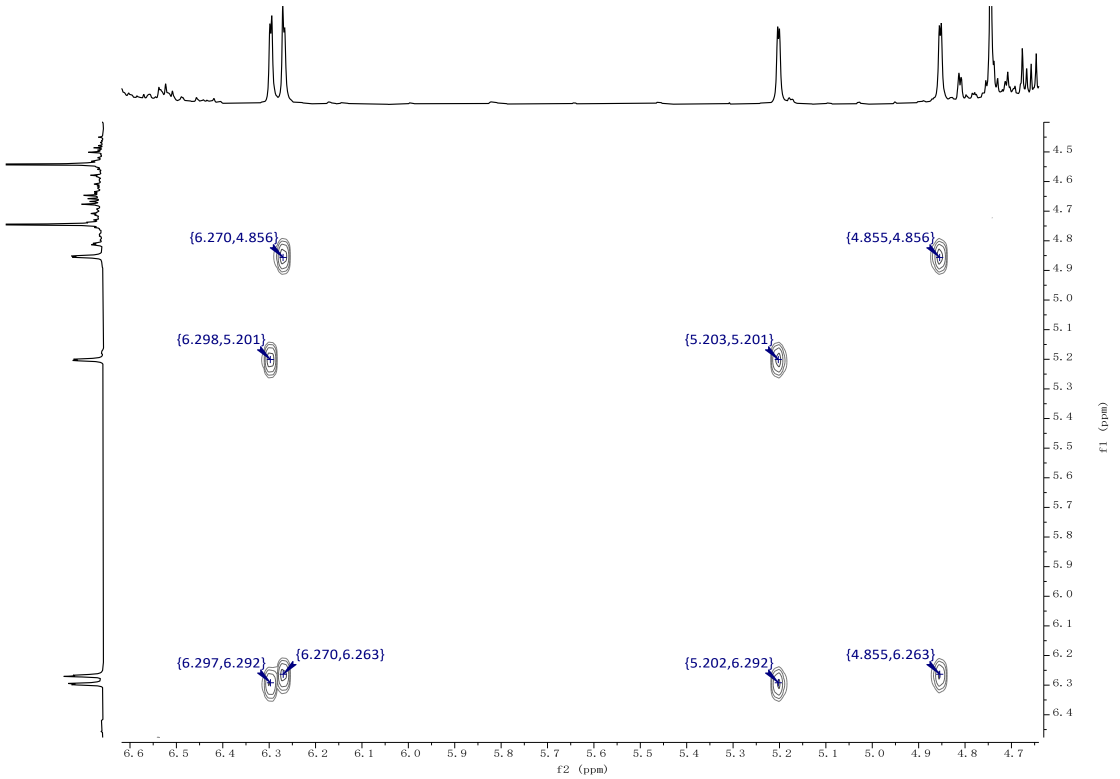

Figure S7. ^{1}H-^{1}H COSY spectrum of **3-^{13}C** with key ^{1}H resonances associated with $^{13}C^{1}$ and C^{2}.

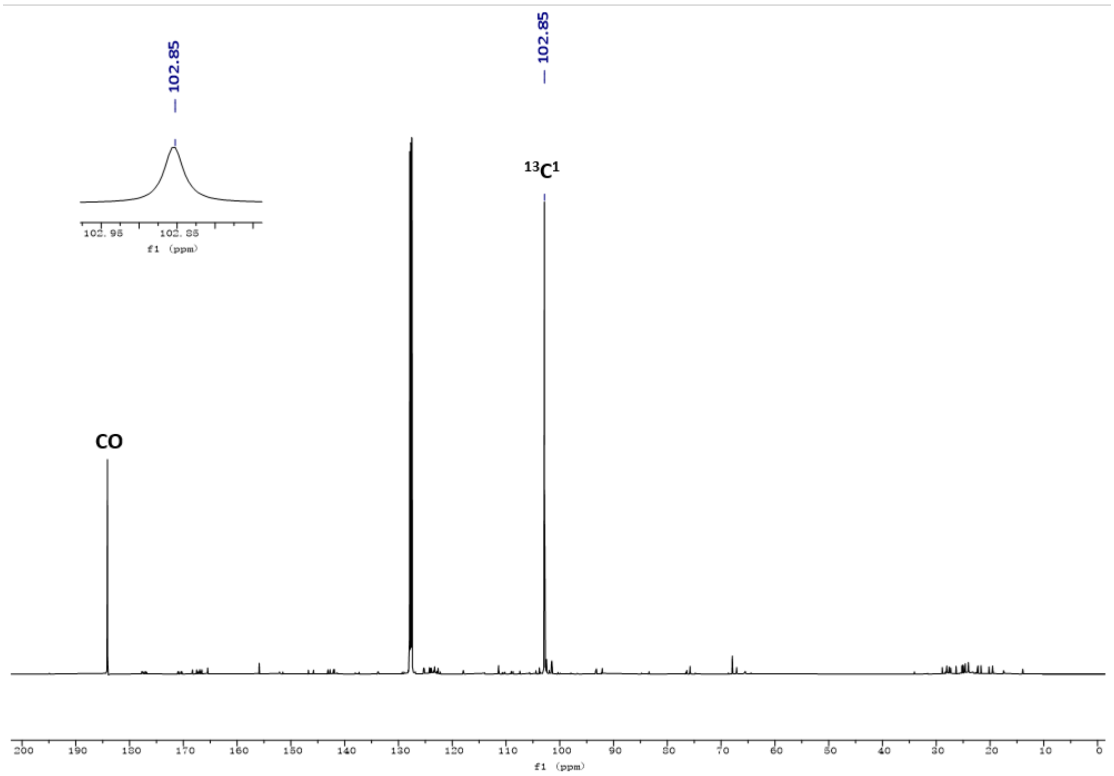

Figure S8. $^{13}C\{^1H\}$ spectrum of **3-^{13}C** with key ^{13}C resonances of $^{13}C^1$.

2.6 DOSY Spectrum of *3_2* and *3-THF$_2$*

Instruments: DOSY spectrums were recorded on BRUKER 500 MHz machines, $Si(SiMe_3)_4$ was used as internal standard (^{1}H).[S4] All peaks are referenced against residual solvent and values are quoted in ppm. Data were processed using the using Dynamics Centre. The hydrodynamic radius was calculated by using Stokes-Einstein Equation.[S4]

DFT calculation also simulated the experimental results (The electronic energies of the optimised geometries and their volumes were calculated using the ωB97X-D functional with 6-311+G** basis sets for all atoms with solvent corrections (PCM, benzene, ε = 2.2706).)

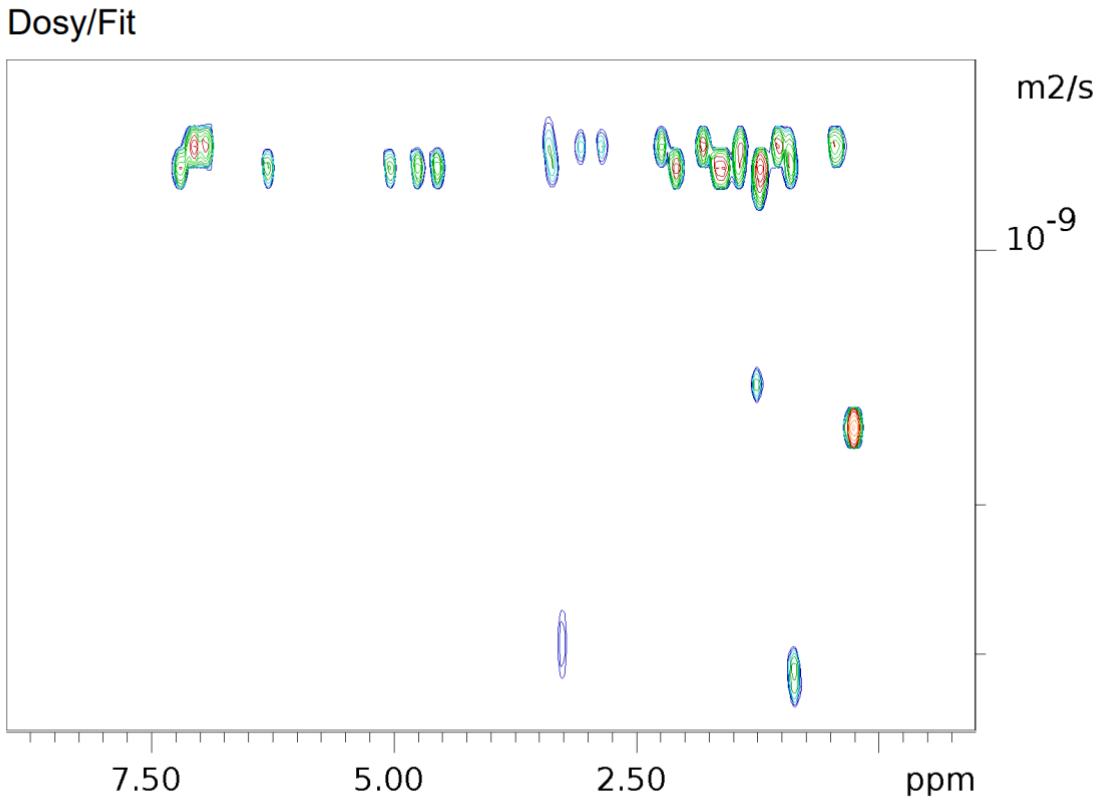

Figure S9. DOSY spectrum of **3_2** in C_6D_6.

Compound diffusion coefficient is: 7.77×10^{-10} m^2/s
Compound HD radius is: 9.3 Å
Compound HD radius (DFT calculation result) is: 8.8 Å

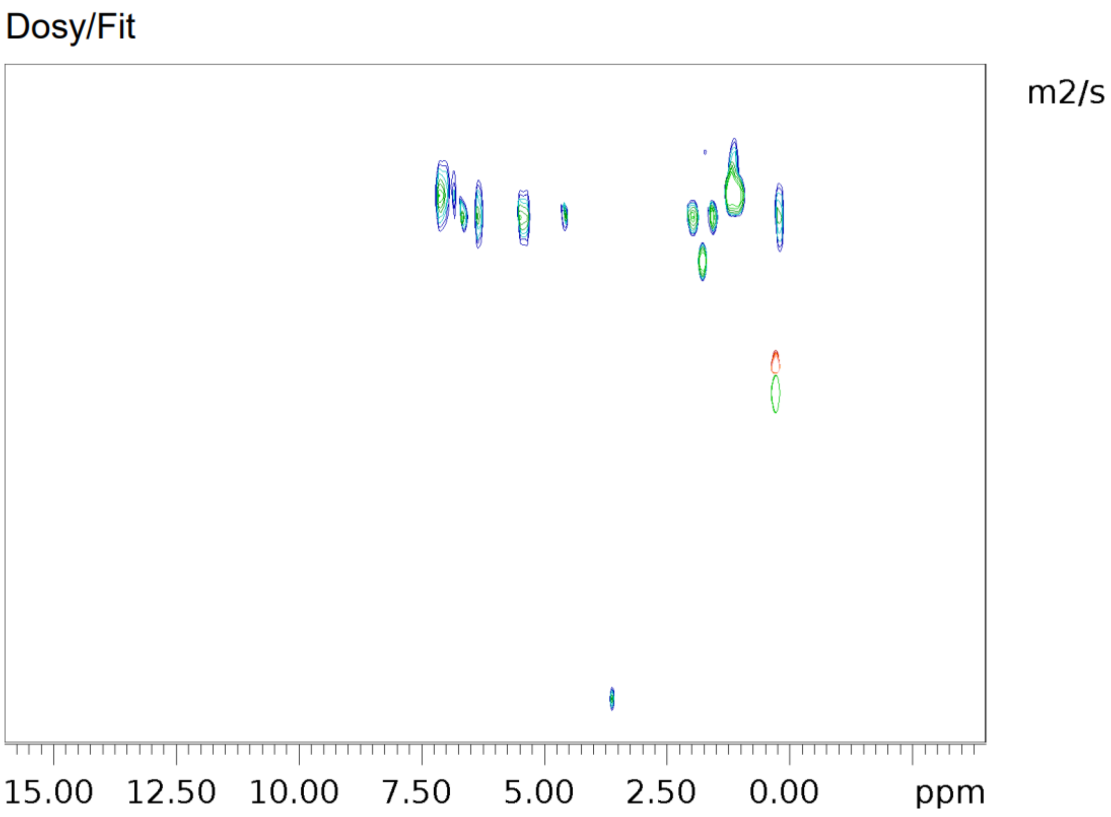

Figure S10. DOSY spectrum of **3-THF$_2$** in d$_8$-THF.

Compound diffusion coefficient is: 1.13 × 10^{-9} m^2/s
Compound HD radius is: 7.2 Å
Compound HD radius (DFT calculation result) is: 7.6 Å

3) Single Crystal X-ray Diffraction Data

data	2a	2b	2c	3_2
**formula**	$C_{86}H_{118}Mg_4N_{10}$	$C_{76}H_{112}Mg_4N_{12}$	$C_{67}H_{93}Mg_2N_5$	$C_{88}H_{118}Mg_4N_{10}O_2$
**solvent**	$1.5(C_4H_{10}O)$	C_6H_6	$C_4H_{10}O$	$4(C_4H_{10}O)$
**formula weight**	1500.32	1369.12	1091.20	1741.63
**colour, habit**	yellow blocks	yellow tablets	colourless tablets	yellow blocks
**temperature / K**	173	173	173	173
**crystal system**	monoclinic	orthorhombi	triclinic	monoclinic
**space group**	$P2_1/c$ (no. 14)	*Pbcn* (no. 60)	P–1 (no. 2)	$C2/c$ (no. 15)
***a* / Å**	20.0772(2)	15.69440(18)	12.2454(3)	23.1551(2)
***b* / Å**	15.05708(15)	30.4384(3)	14.1302(4)	13.13534(12)
***c* / Å**	30.2204(3)	17.51319(16)	20.6959(5)	33.9649(4)
**α / deg**	90	90	78.593(2)	90
**β / deg**	93.6657(9)	90	85.703(2)	93.0767(9)
**γ / deg**	90	90	73.328(3)	90
**V / Å^3**	9117.04(15)	8366.26(14)	3362.09(17)	10315.55(18)
Z	4	4 [c]	2	4 [c]
**D_c / g cm^{-3}**	1.093	1.087	1.078	1.121
**radiation used**	Cu-Kα	Cu-Kα	Cu-Kα	Cu-Kα
**μ / mm^{-1}**	0.745	0.763	0.645	0.754
**no. of unique reflns**				
**measured (R_{int})**	17484	8221	13374	10391
**obs, $\|F_o\| > 4\sigma(\|F_o\|)$**	13517	6818	9573	8324
**completeness (%)** [a]	98.5	99.7	100	100
**no. of variables**	1055	504	740	588
**R_1(obs), wR_2(all)** [b]	0.0454,	0.0371,	0.0523,	0.0597,
**CCDC code**	2289252	2308758	2308759	2308757

[a] Completeness to 0.84 Å resolution. [b] $R_1 = \Sigma||F_o| - |F_c||/\Sigma|F_o|$; $wR_2 = \{\Sigma[w(F_o^2 - F_c^2)^2] / \Sigma[w(F_o^2)^2]\}^{1/2}$; $w^{-1} = \sigma^2(F_o^2) + (aP)^2 + bP$. [c] The complex has crystallographic C_2 symmetry.

Table S1. Crystal Data, Data Collection and Refinement Parameters for the structures of **2a**, **2b**, **2c** and $\mathbf{3_2}$.

data	3-THF$_2$	4a
formula	$C_{52}H_{75}Mg_2N_5O_3$	$C_{33}H_{40}AlN_3O$
solvent	—	$0.5(C_5H_{12})$
formula weight	866.79	557.73
colour, habit	yellow blocks	yellow platy shards
temperature / K	173	173
crystal system	triclinic	monoclinic
space group	*P*–1 (no. 2)	$P2_1/c$ (no. 14)
a / Å	9.2215(3)	17.9825(6)
b / Å	11.1028(4)	12.3081(4)
c / Å	27.4961(7)	14.6715(4)
α / deg	80.246(3)	90
β / deg	84.648(3)	95.560(3)
γ / deg	67.366(3)	90
V / Å^3	2559.58(16)	3231.97(17)
Z	2	4
D_c / g cm^{-3}	1.125	1.146
radiation used	Cu-Kα	Cu-Kα
μ / mm^{-1}	0.760	0.775
no. of unique reflns		
measured (R_{int})	10104	6212 (0.0413)
obs, $\|F_o\| > 4\sigma(\|F_o\|)$	7322	4201
completeness (%) [a]	99.7	98.8
no. of variables	590	356
R_1(obs), wR_2(all) [b]	0.0513,	0.0526,
CCDC code	2313439	2328581

Table S2. Crystal Data, Data Collection and Refinement Parameters for the structures of **3-THF$_2$** and **4a**.

Table S1 provides a summary of the crystallographic data for the structures of **2a**, **2b**, **2c**, **3_2**, **3-THF$_2$** and **4a**. Data were collected using an Agilent Xcalibur PX Ultra A diffractometer, and the structures were solved and refined using the OLEX2,[S5] SHELXTL[S6] and SHELX-2013[S7] program systems. CCDC 2289252, 2308758, 2308759, 2308757, 2313439 and 2328581 respectively.

The X-ray crystal structure of 2a

The N81-bound 2,6-dimethylphenyl group and the O100-based included diethylether solvent molecule in the structure of **2a** were both found to be disordered, and in each case two orientations were identified, of *ca*. 82:18 and 70:30% occupancy respectively. The geometries of each pair of orientations were optimised, the thermal parameters of adjacent atoms were restrained to be similar, and only the non-hydrogen atoms of the major occupancy orientations were refined anisotropically (those of the minor occupancy orientations were refined isotropically). The O110-based included diethylether solvent molecule was found to be disordered across a centre of symmetry, and two unique orientations were identified of *ca*. 27 and 23% occupancy (with two further orientations of the same occupancies being generated by operation of the inversion centre). The geometries of the two unique orientations were optimised, the thermal parameters of adjacent atoms were restrained to be similar, and all of the atoms were refined isotropically. The two bridging hydride atoms were both located from Δ*F* maps and refined freely.

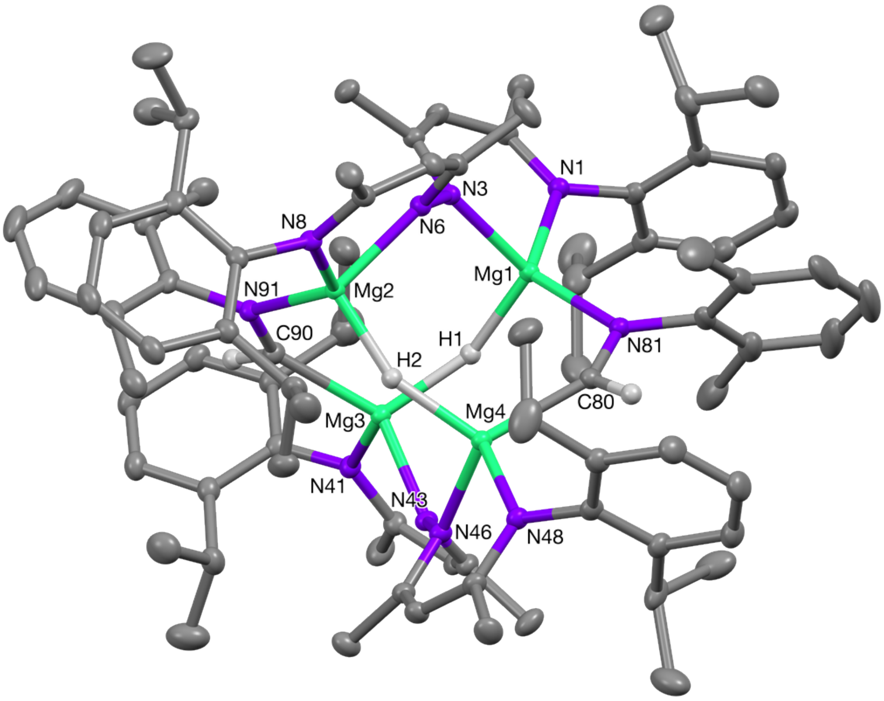

Figure S11. The crystal structure of **2a** (20% probability ellipsoids). Hydrogen atoms with the exception of hydride ligands have been omitted for clarity.

The X-ray crystal structure of 2b

The structure of **2b** was found to sit across a C_2 axis that passes through the middle of both of the two $C_2N_2Mg_2$ rings and bisects the Mg1···Mg1A and Mg2···Mg2A vectors. The C17-, C20- and C29-based *iso*-propyl groups were all found to be disordered, and in each case two orientations were identified of *ca*. 69:31, 78:22 and 70:30% occupancy. The geometries of each pair of orientations were optimised, the thermal parameters of adjacent atoms were restrained to be similar, and only the non-hydrogen atoms of the major occupancy orientations were refined anisotropically (those of the minor occupancy orientations were refined isotropically). The C61-based included benzene solvent molecule was found to be disordered across a C_2 axis, and this was modelled by using one complete, 50% occupancy orientation, with a further orientation being generated by operation of the inversion centre. The geometry of the unique orientation was idealised, the thermal parameters of adjacent atoms were restrained to be similar, and all the non-hydrogen atoms were refined anisotropically.

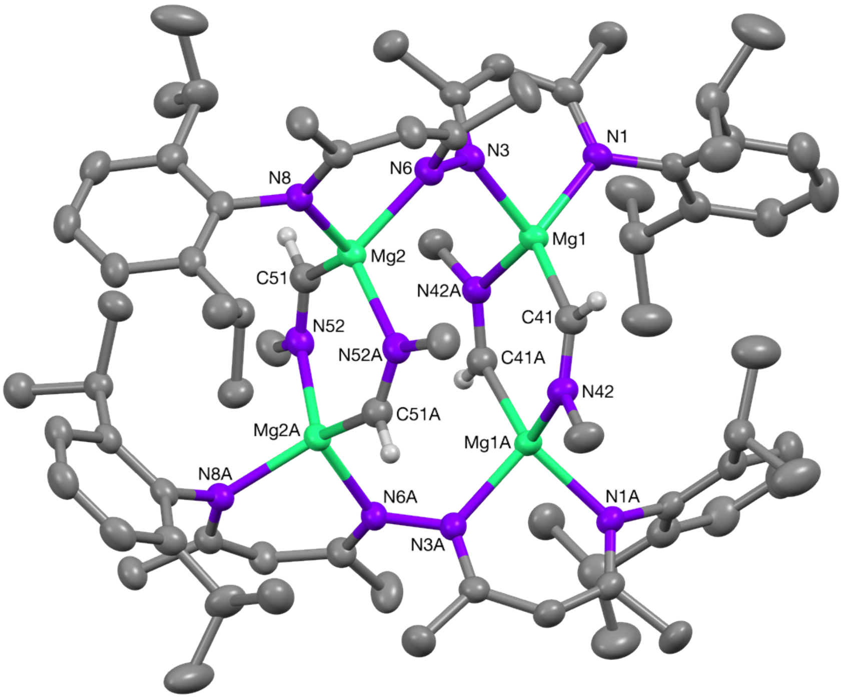

Figure S12. The crystal structure of the C_2-symmetric complex **2b** (50% probability ellipsoids). Hydrogen atoms with the exception of hydride ligands have been omitted for clarity.

The X-ray crystal structure of 2c

The bridging hydride atom in the structure of **2c** was both located from a Δ*F* map and refined freely.

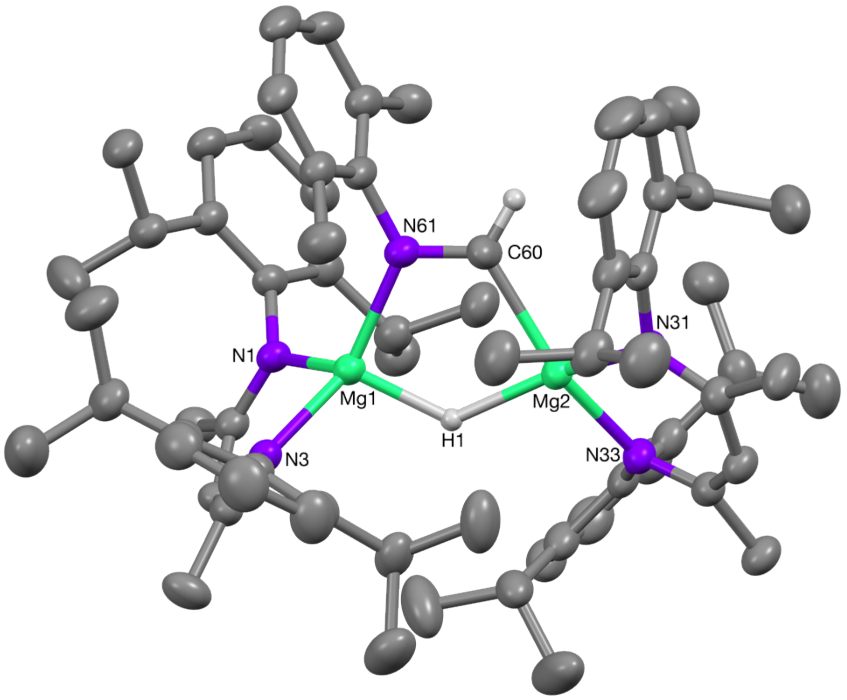

Figure S13. The crystal structure of **2c** (50% probability ellipsoids). Hydrogen atoms with the exception of hydride ligands have been omitted for clarity.

The X-ray crystal structure of $\mathbf{3_2}$

The structure of $\mathbf{3_2}$ was found to sit across a C_2 axis that passes through the middle of the Mg_2O_2 ring and bisects the Mg2···Mg2A vector. The C20-based *iso*-propyl group was found to be disordered, and two orientations were identified of *ca*. 83 and 17% occupancy. Their geometries were optimised, the thermal parameters of adjacent atoms were restrained to be similar, and only the non-hydrogen atoms of the major occupancy orientation were refined anisotropically (those of the minor occupancy orientation were refined isotropically).

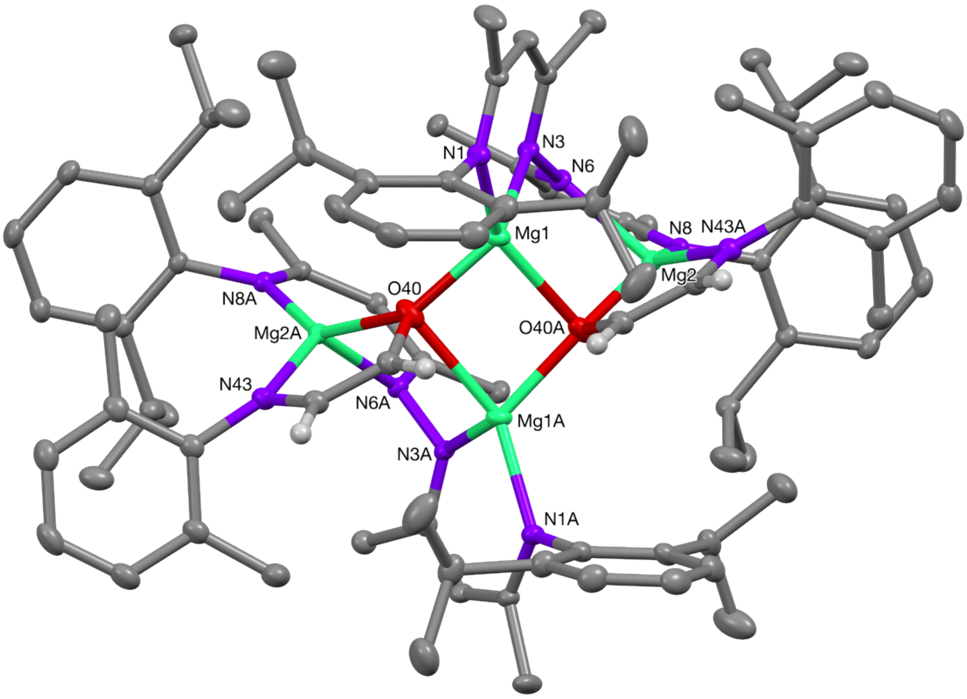

Figure S14. The crystal structure of the C_2-symmetric complex $\mathbf{3_2}$ (20% probability ellipsoids). Hydrogen atoms with the exception of hydride ligands have been omitted for clarity.

The X-ray crystal structure of 3-THF$_2$

The O60-based coordinated thf solvent molecule in the structure of **3-THF$_2$** was found to be disordered, and two orientations for the C_4H_8 linkage were identified of *ca*. 71 and 29% occupancy. Their geometries were optimised, the thermal parameters of adjacent atoms were restrained to be similar, and only the non-hydrogen atoms of the major occupancy orientation were refined anisotropically (those of the minor occupancy orientation were refined isotropically).

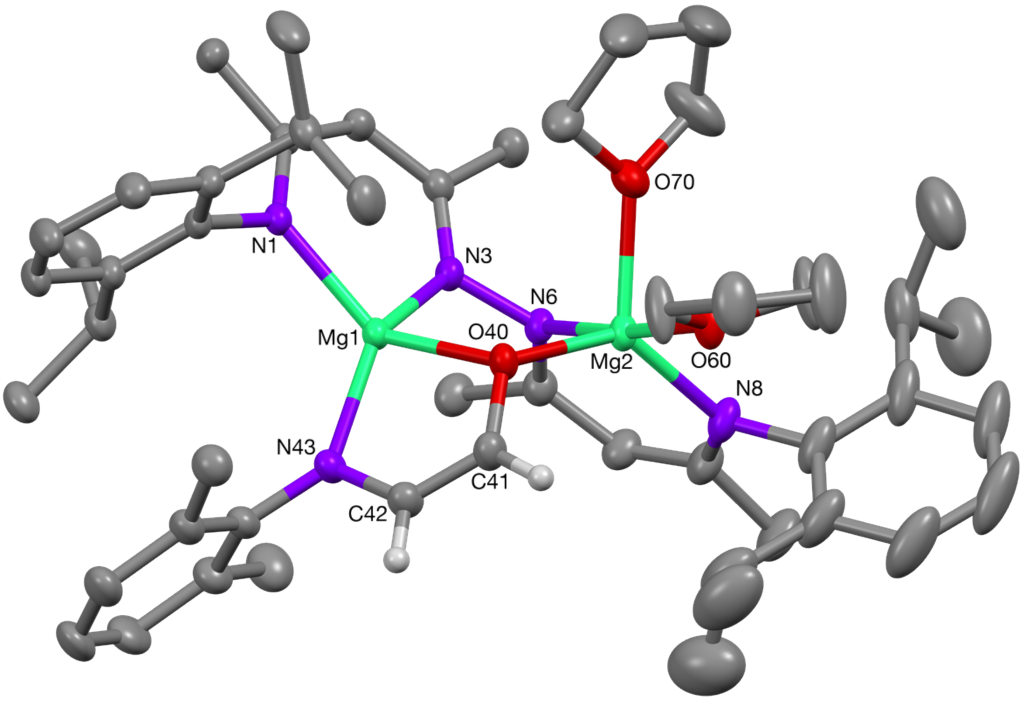

Figure S15. The crystal structure of **3-THF$_2$** (30% probability ellipsoids). Hydrogen atoms with the exception of hydride ligands have been omitted for clarity.

The X-ray crystal structure of 4a

The included solvent in the structure of **4a** was found to be highly disordered, and the best approach to handling this diffuse electron density was found to be the SQUEEZE routine of PLATON.[S8] This suggested a total of 79 electrons per unit cell, equivalent to 19.8 electrons per complex. With the refinements from before the use of SQUEEZE giving no clear indication as to the identity of the solvent, unsurprisingly fitting equally well to both pentane (C_5H_{12}, 42e) and diethyl ether ($C_4H_{10}O$, 42e), the most recently used solvent (pentane) was assumed. 0.5 pentane molecules correspond to 21 electrons, so this was used as the solvent present. As a result, the atom list for the asymmetric unit is low by $0.5(C_5H_{12}) = C_{2.5}H_6$ (and that for the unit cell low by $C_{10}H_{24}$) compared to what is actually presumed to be present.

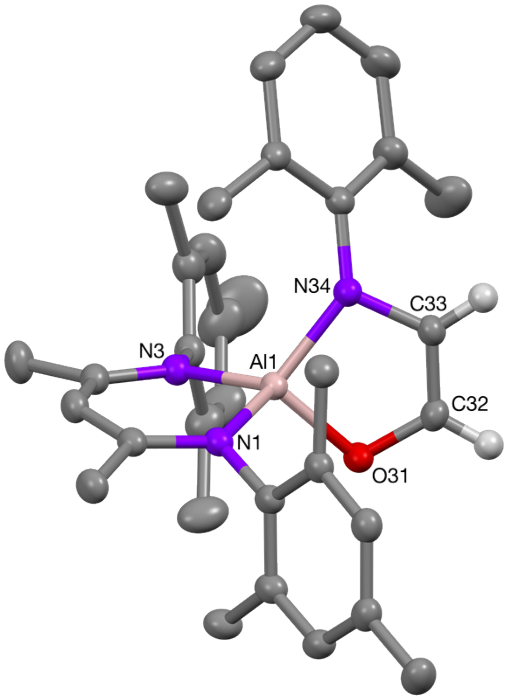

Figure S16. The crystal structure of **4a** (30% probability ellipsoids). Hydrogen atoms with the exception of hydride ligands have been omitted for clarity.

4) DFT Studies

4.1. Computational methods

DFT calculations were performed using Gaussian 09 (Revision D.01) using an ultrafine integration grid (int=ultrafine).[S9] Geometry optimisations and frequency calculations were performed using the B3PW91 density functional including solvent (PCM, benzene, ε = 2.2706) and dispersion corrections (D3) with SDDAll (Mg), 6-31G** (C, H) and 6-31+G* (N, O) basis set. Frequency analyses for all stationary points were performed using the enhanced criteria to confirm the nature of the structures as either minima (no imaginary frequency) or transition states (only one imaginary frequency). The electronic energies of the optimised geometries were calculated using the B3PW91 functional including solvent and dispersion corrections (D3) with 6-311+G** basis sets for all atoms with solvent corrections (PCM, benzene, ε = 2.2706). The Gibbs free energy correction from the frequency calculation was added to this electronic energy to generate Gibbs free energy values for the calculated stationary points.

Intrinsic reaction coordinate (IRC) calculations were used to connect transition states and minima located on the potential energy surface allowing a full energy profile (calculated at 298.15 K, 1 atm.) of the reaction to be constructed.

NBO analysis was performed at the B3PW91/6-311+G** level with dispersion corrections (D3) and solvent corrections (PCM, benzene, ε = 2.2706) for all atoms by using NBO 6.0.[S10]

QTAIM was performed at the B3PW91/6-311+G** level with dispersion corrections (D3) and solvent corrections (PCM, benzene, ε = 2.2706) for all atoms by using Gaussian 09. QTAIM analysis was conducted using the AIMAll software. [S11-S12]

4.2. Calculated Reaction Pathway

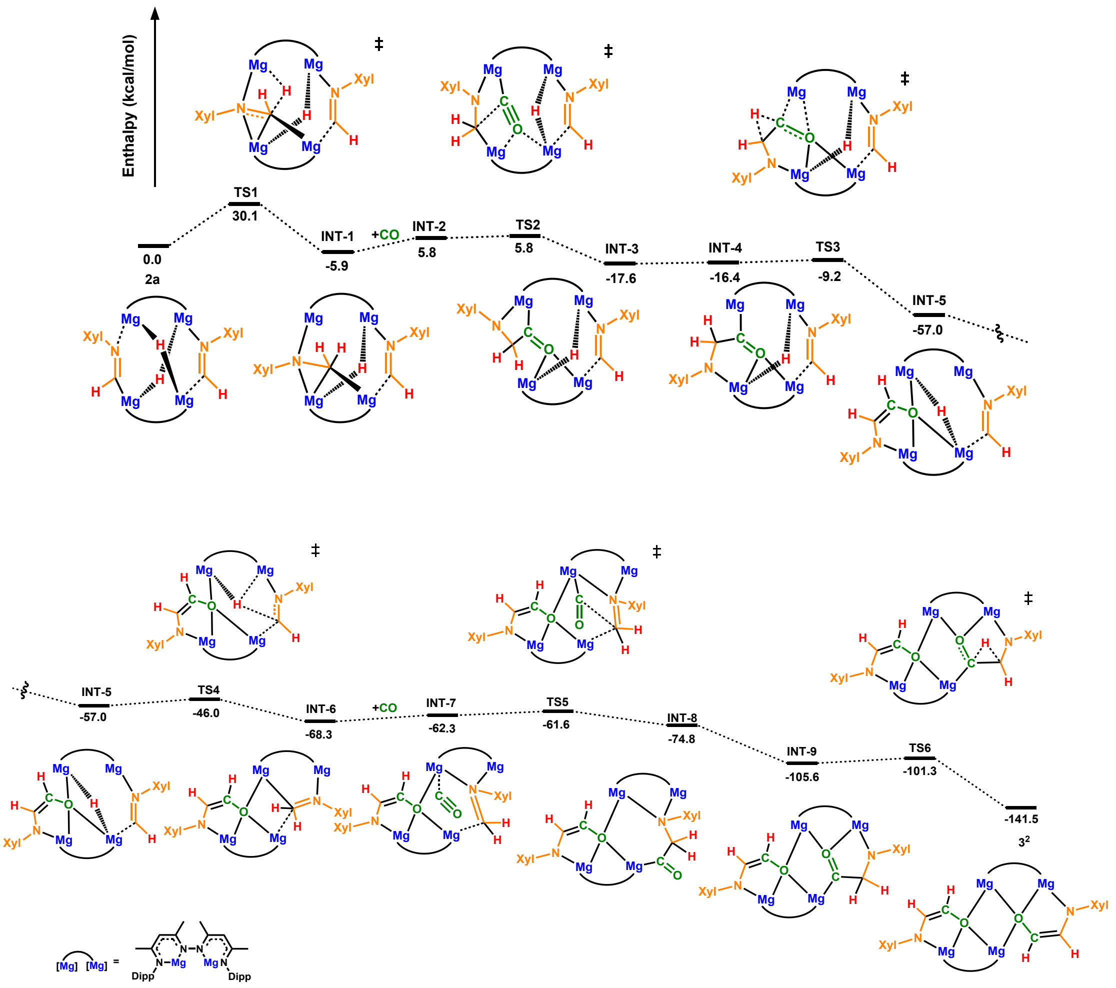

Figure S17. Proposed pathway for the coupling of CO and Isocyanide based on DFT calculation G09 (Enthalpy). Level of theory: B3PW91-D3 / 6-311+G**/ PCM (benzene) // B3PW91-D3 / 6-31G** (C,H,N,O) / SDDAll (Mg) / PCM (benzene). Enthalpy kcal mol^{-1}.

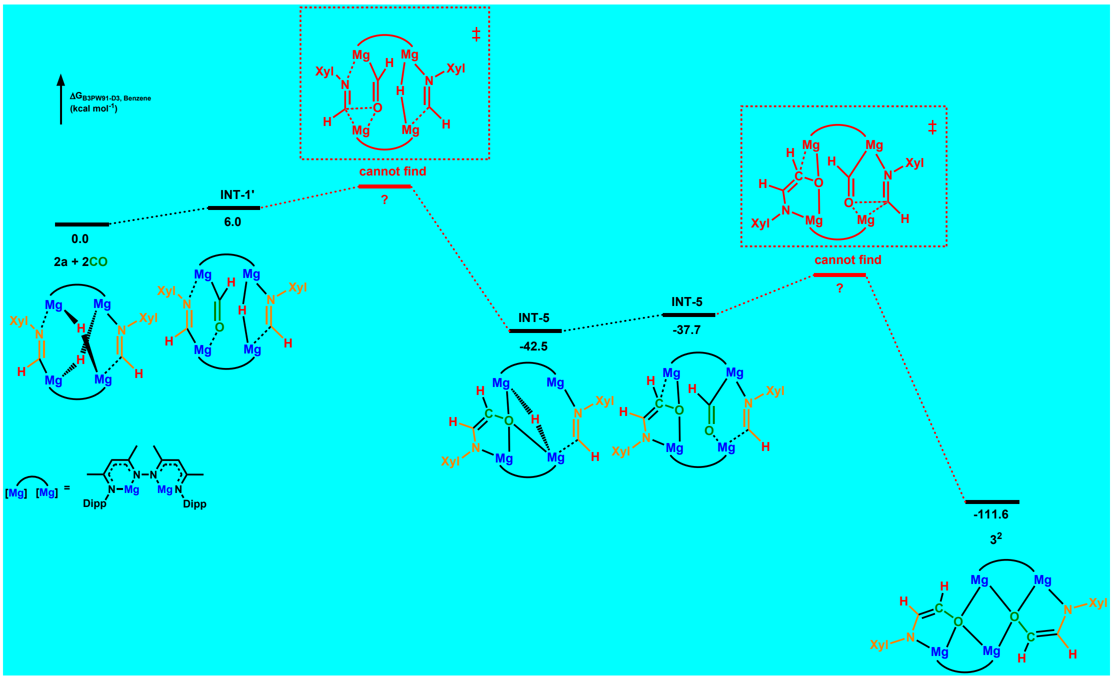

Figure S18. Proposed pathway for the coupling of CO and Isocyanide based on DFT calculation G09 (Enthalpy). Level of theory: B3PW91-D3 / 6-311+G**/ PCM (benzene) // B3PW91-D3 / 6-31G** (C,H,N,O) / SDDAll (Mg) / PCM (benzene). Gibbs Energies kcal mol-1.

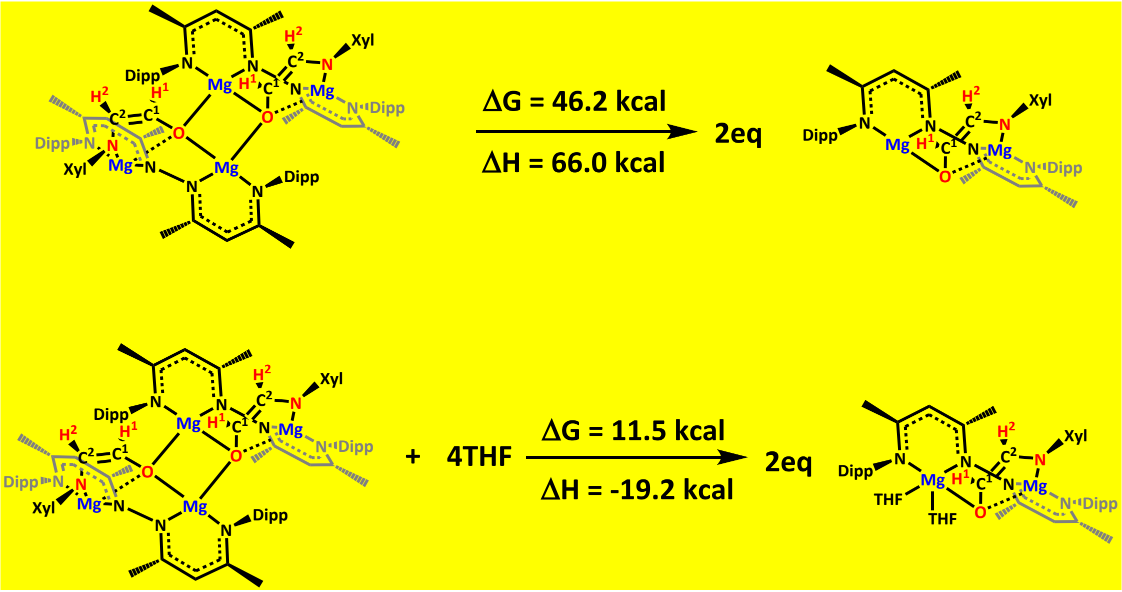

Figure S19. Thermodynamics of monomer formation from $\mathbf{3_2}$ in the absence and presence of THF. Level of theory: B3PW91-D3 / 6-311+G**/ PCM (benzene) // B3PW91-D3 / 6-31G** (C,H,N,O) / SDDAll (Mg) / PCM (benzene). Gibbs Energies kcal mol-1.

4.3. NBO Analysis of 3_2, 3-THF$_2$, and 4.

NBO analysis was performed at the B3PW91/6-311+G** level with dispersion corrections (D3) and solvent corrections (PCM, benzene, ε = 2.2706) for all atoms by using NBO 6.0.[S10]

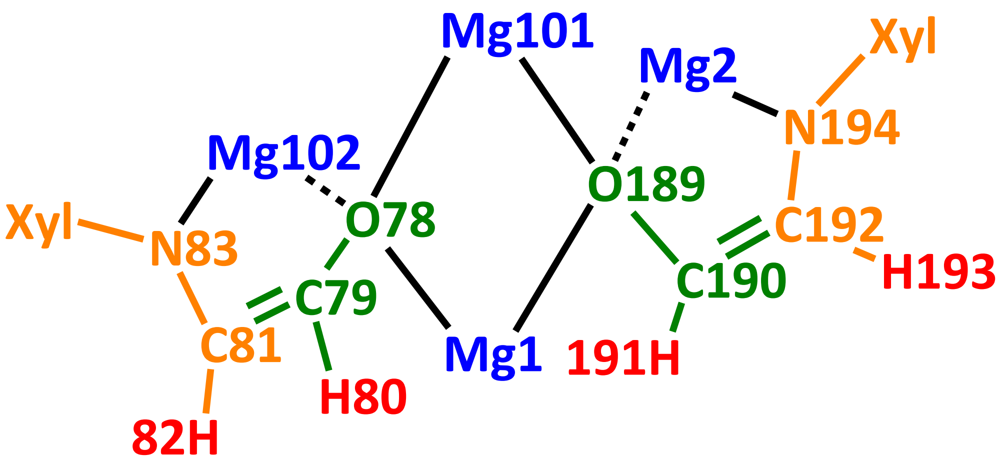

3_2

Table S3. NPA change data for **3_2**. (The β-diketiminate ligand has been omitted for clarity)
Level of theory: B3PW91-D3 / 6-311+G**/ PCM (benzene).

Atom	No	Charge
Mg1	1	1.83
Mg2	2	1.82
Mg101	101	1.84
Mg102	102	1.82
H80	80	0.20
H82	82	0.20
H191	191	0.21
H193	193	0.20
C79	79	-0.19
C81	81	-0.01
C190	190	-0.25
C192	192	0.01
N83	83	-0.94
N194	194	-0.91
O78	78	-1.20
O189	189	-1.19

Table S4. WBI data for $\mathbf{3_2}$. Level of theory: B3PW91-D3 / 6-311+G**/ PCM (benzene).

**Wiberg bond index matrix**	
Mg1 - O78	0.036
Mg1 - O189	0.022
Mg101 - O78	0.023
Mg101 - O189	0.033
Mg2 - O189	0.032
Mg2 - N194	0.061
Mg102 - O78	0.032
Mg102 - N83	0.063
C79 - O78	0.937
C79 - C81	1.624
C79 - H80	0.902
C81 - N83	1.249
C81 - H82	0.902
C190 - O189	0.939
C190 - C192	1.569
C190 - H191	0.897
C192 - N194	1.296
C192 - H193	0.902

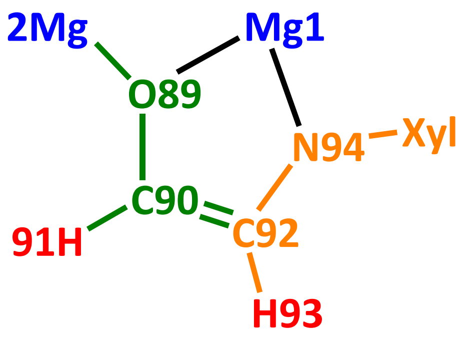

Table S5. NPA change data for **3-THF$_2$**. (The β-diketiminate ligand has been omitted for clarity) Level of theory: B3PW91-D3 / 6-311+G**/ PCM (benzene).

Atom	No	Charge
Mg1	1	1.81
Mg2	2	1.82
C90	90	-0.03
C92	92	-0.08
N94	94	-0.96
O89	89	-1.16
H91	91	0.17
H93	93	0.19

Table S6. WBI data for **3-THF$_2$**. (The β-diketiminate ligand has been omitted for clarity) Level of theory: B3PW91-D3 / 6-311+G**/ PCM (benzene).

Wiberg bond index matrix	
Mg1 - O89	0.038
Mg2 - O89	0.037
Mg1 - N94	0.079
C90 - O89	0.985
C90 - C92	1.706
C92 - N94	1.150
C90 - H91	0.917
C92 - C93	0.912

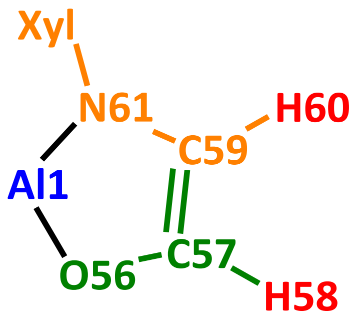

Table S7. NPA change data for **4**. (The β-diketiminate ligand has been omitted for clarity) Level of theory: B3PW91-D3 / 6-311+G**/ PCM (benzene).

Atom	No	Charge
Al1	1	2.15
C57	57	0.07
C59	59	-0.12
O56	56	-0.98
N61	61	-1.00
H58	58	0.18
H60	60	0.21

Table S8. WBI data for **4**. (The β-diketiminate ligand has been omitted for clarity) Level of theory: B3PW91-D3 / 6-311+G**/ PCM (benzene).

Wiberg bond index matrix	
Al1 - O56	0.327
Al1 - N61	0.335
C57 - O56	1.024
C57 - C59	1.763
C59 - N61	1.044
C59 - H60	0.909
C57 - H58	0.915

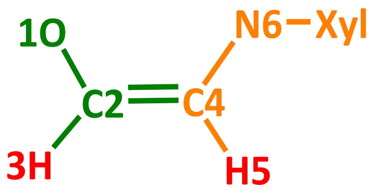

Table S9. NPA change data for **cis-enamidolate dianion**. Level of theory: B3PW91-D3 / 6-311+G**/ PCM (benzene).

Atom	No	Charge
O1	1	-0.91
C2	2	0.13
C4	4	-0.25
N6	6	-0.67
H3	3	0.05
H5	5	0.13

Table S10. WBI data for **cis-enamidolate dianion**. Level of theory: B3PW91-D3 / 6-311+G**/ PCM (benzene).

Wiberg bond index matrix	
O1 - C2	1.285
C2 - C4	1.616
C4 - N6	1.111
C2 - H3	0.905
C4 - H5	0.922

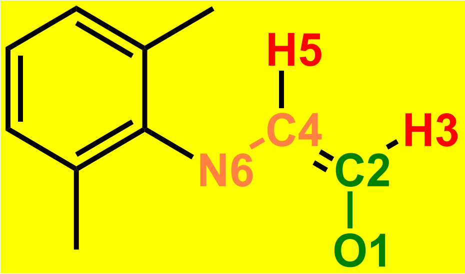

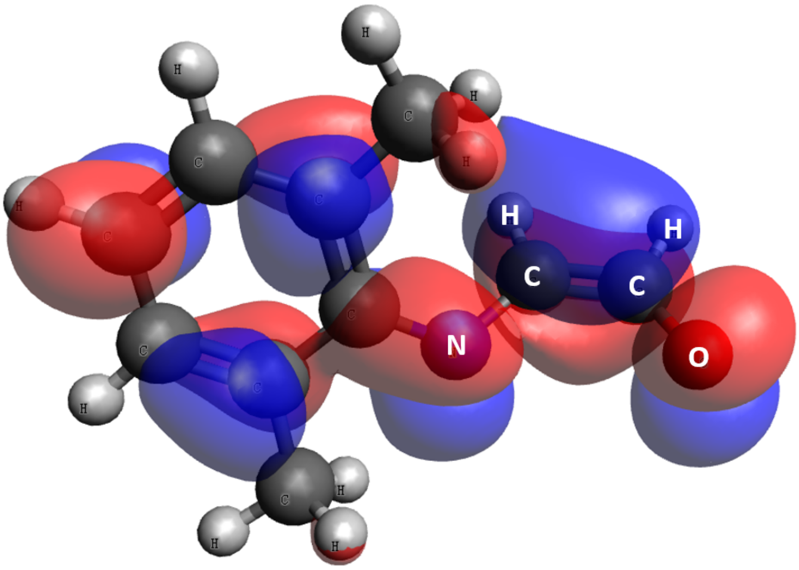

HOMO
π-donor

Figure S20. HOMO of cis-enamidolate dianion (isovalue = 0.02)

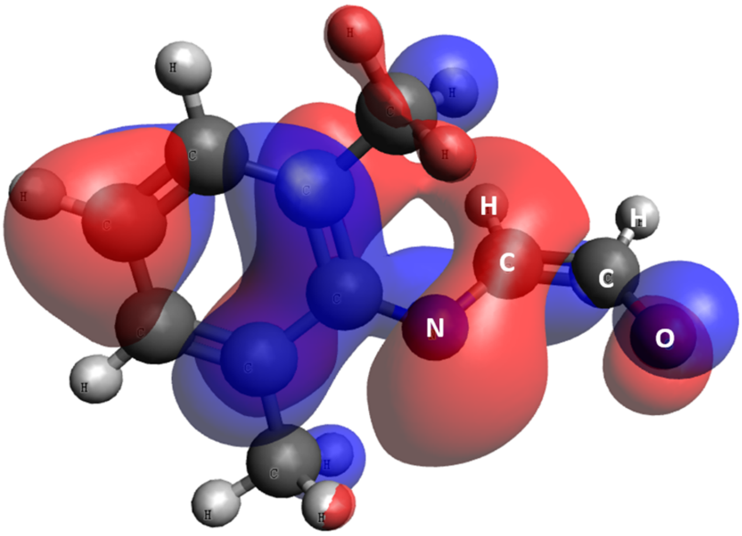

HOMO-1
N-LP σ-donor

Figure S21. HOMO-1 of cis-enamidolate dianion (isovalue = 0.02)

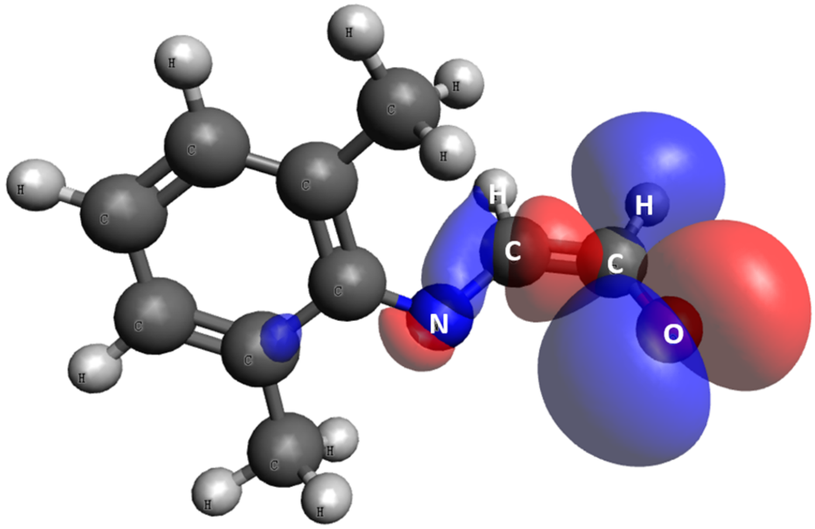

HOMO-2
O-LP σ-donor

Figure S22. HOMO-2 of cis-enamidolate dianion (isovalue = 0.02)

4.4. QTAIM Analysis

QTAIM was performed at the B3PW91/6-311+G** level with dispersion corrections (D3) and solvent corrections (PCM, benzene, ε = 2.2706) for all atoms by using Gaussian 09. QTAIM analysis was conducted using the AIMAll software.[S11-S12] The AIM analysis returns bond paths and bond critical points in key atoms in the core structure. In particular, for the ethene amidolate ligand bond paths are located between N and Mg, O and Mg, O and C, C and C, and C and N creating a ring structure. A ring critical point is located in the centre of this structure. ρ(r) values are consistent with ionic bonding between Mg and N and Mg and O (Table S11). AIM also shows a very weak electrostatic interaction between the two O atoms of the central Mg_2O_2 core of the molecule.

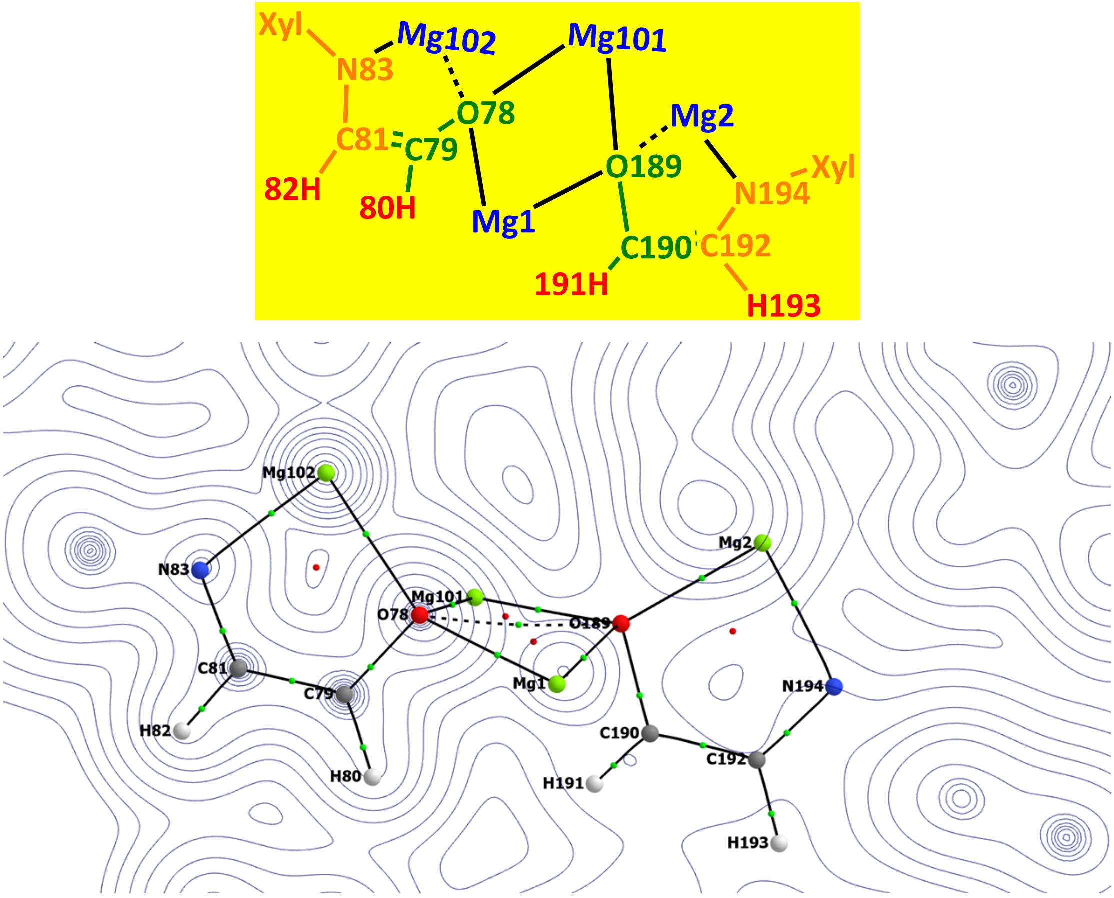

Figure S23. QTAIM molecular graphs for $\mathbf{3_2}$. (QTAIM methodology level: B3PW91-D3 / 6-311+G**/ PCM (benzene)). The β-diketiminate ligand has been omitted for clarity. Coodinates of 3 points in plane. Point One: Mg102; Point Two: O78; Point Three: C79. Bond critical points shown in green, ring critical points shown in red. Electron density value below 10^{-5} not be shown. Bond paths terminating at a critical point with an electron density which below 0.025 will be shown as dashed (instead of solid). Bond critical points with an electron density above 0.025 will be shown as solid lines.

Table S11. QTAIM data for $\mathbf{3_2}$. (QTAIM methodology level: B3PW91-D3 / 6-311+G**/ PCM (benzene))

**Atoms**	**r (r) (e bohr^{-3})**	**∇^2 r (r) (e bohr^{-5})**	**Ellipticity**
O78 - O189	0.022207	0.052476	0.933118
O78 - Mg102	0.032328	0.212114	0.045635
O78 - Mg101	0.03538	0.242529	0.059442
O78 - C79	0.244303	-0.314433	0.162553
O189 - C190	0.24428	-0.327023	0.185121
N83 - Mg102	0.050172	0.328751	0.117469
Mg2 - O189	0.035176	0.239139	0.053894
Mg2 - N194	0.048806	0.318146	0.107647
Mg101 - O189	0.046748	0.353249	0.091705
Mg1 - O78	0.044735	0.332535	0.092657
Mg1 - O189	0.031177	0.206858	0.092939
C81 - N83	0.317926	-0.910697	0.118588
C81 - H82	0.278559	-0.94939	0.026522
C79 - H80	0.277912	-0.927166	0.098807
C79 - C81	0.326292	-0.93069	0.410372
C192 - N194	0.324221	-0.92519	0.118233
C192 - H193	0.279323	-0.955642	0.023157
C190 - H191	0.27585	-0.912478	0.102157
C190 - C192	0.321478	-0.910846	0.377185

5) NMR Spectra

5.1. NMR Spectra of Isolated complexes

^1H NMR (C_6D_6, 400 MHz) of **2a**: "*"is the peak of diethyl ether.

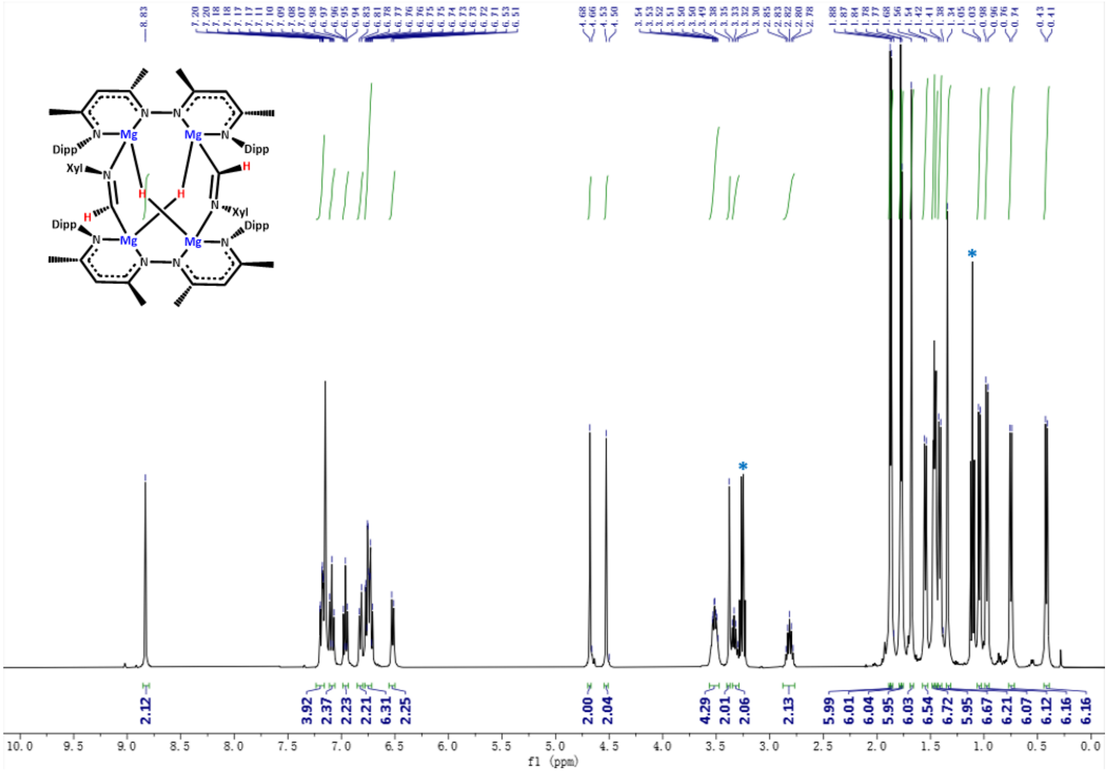

^{13}C NMR (C_6D_6, 101 MHz) of **2a**: "*"is the peak of diethyl ether.

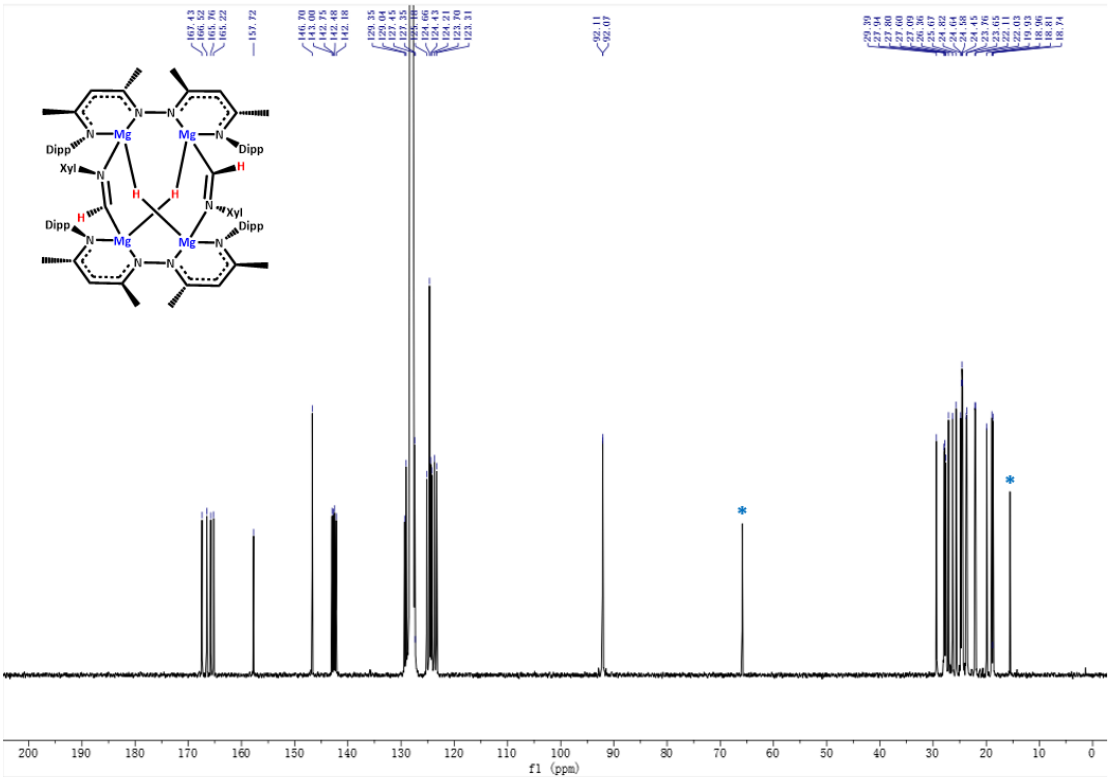

^{1}H NMR (C_6D_6, 400 MHz) of **2b**: "*"is the peak of some impurity which is unknown.

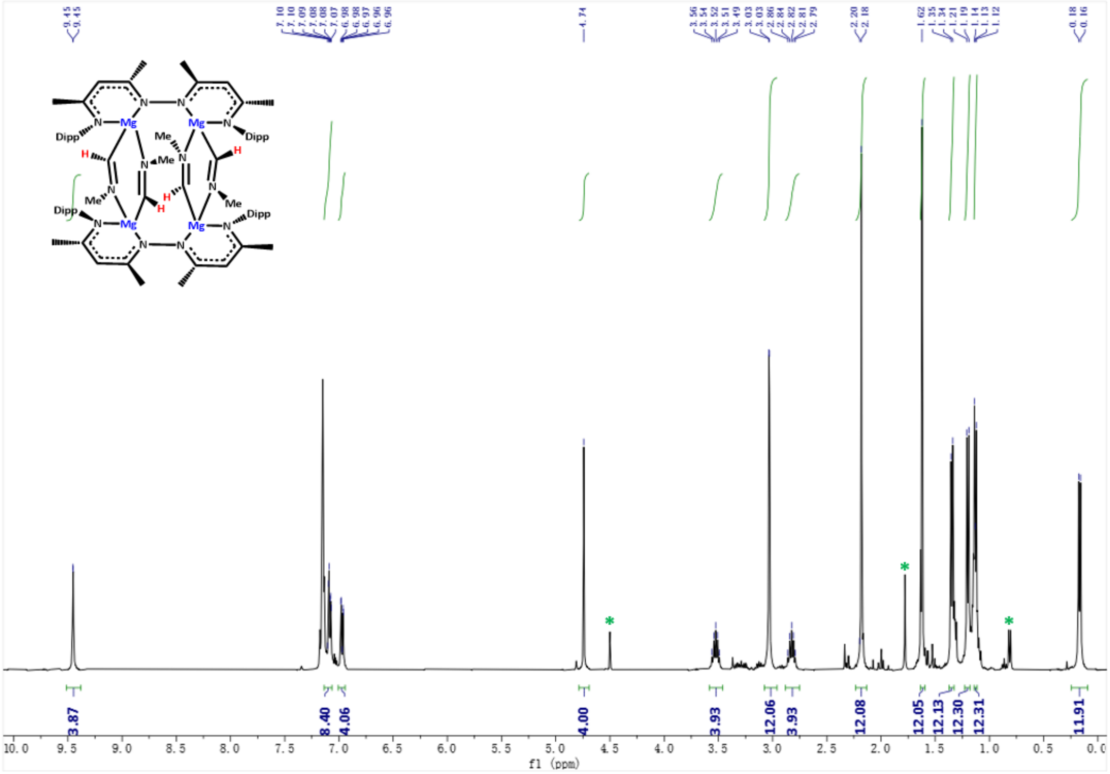

^{13}C NMR (C_6D_6, 101 MHz) of **2b**:

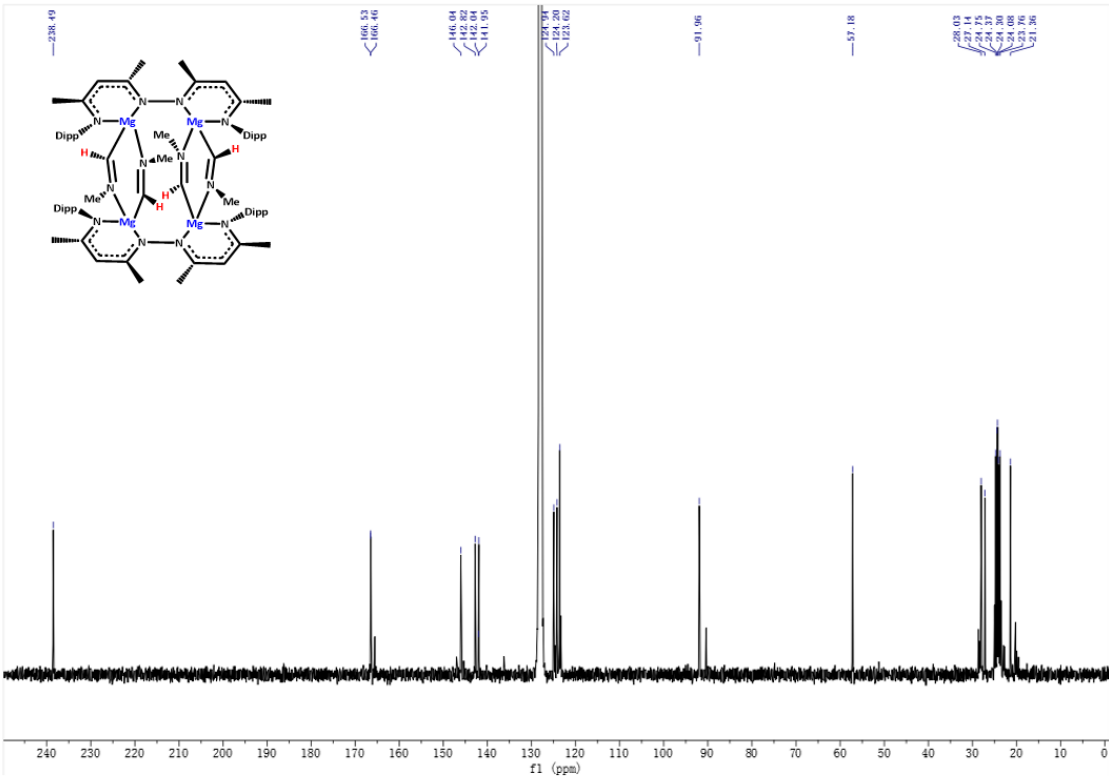

^{1}H NMR (C_6D_6, 400 MHz) of $\mathbf{3_2}$: "*"is the peak of diethyl ether.

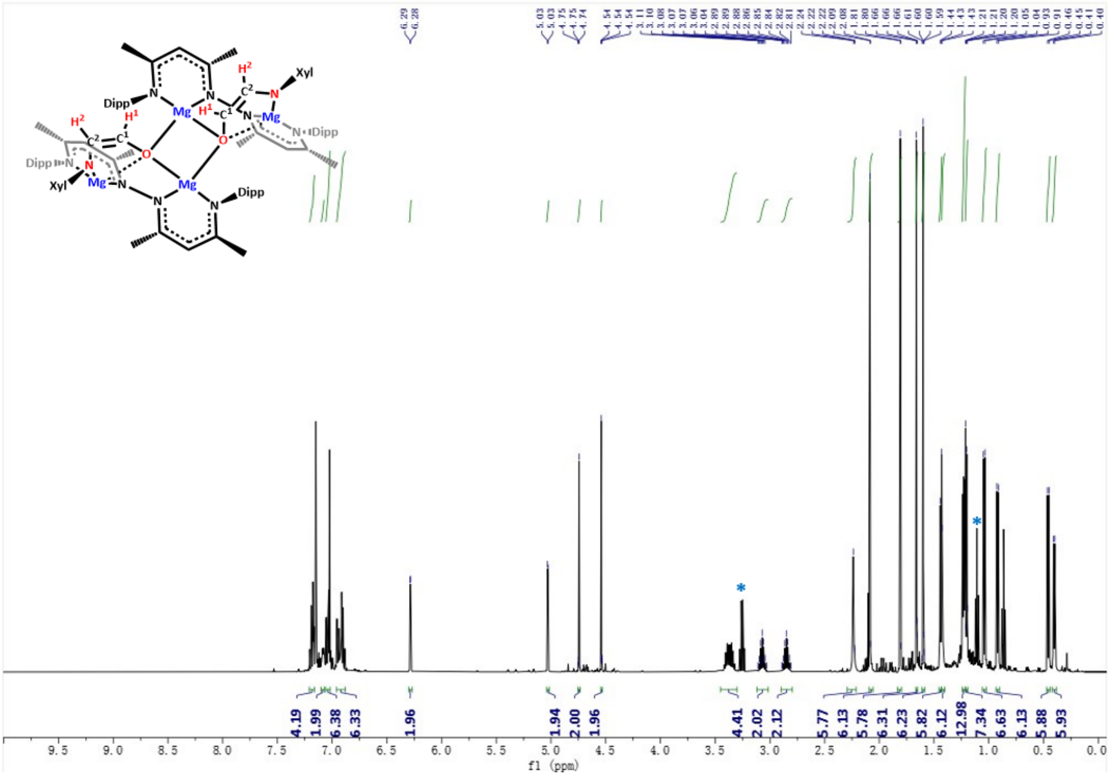

^{13}C NMR (C_6D_6, 101 MHz) of $\mathbf{3_2}$: "*"is the peak of diethyl ether.

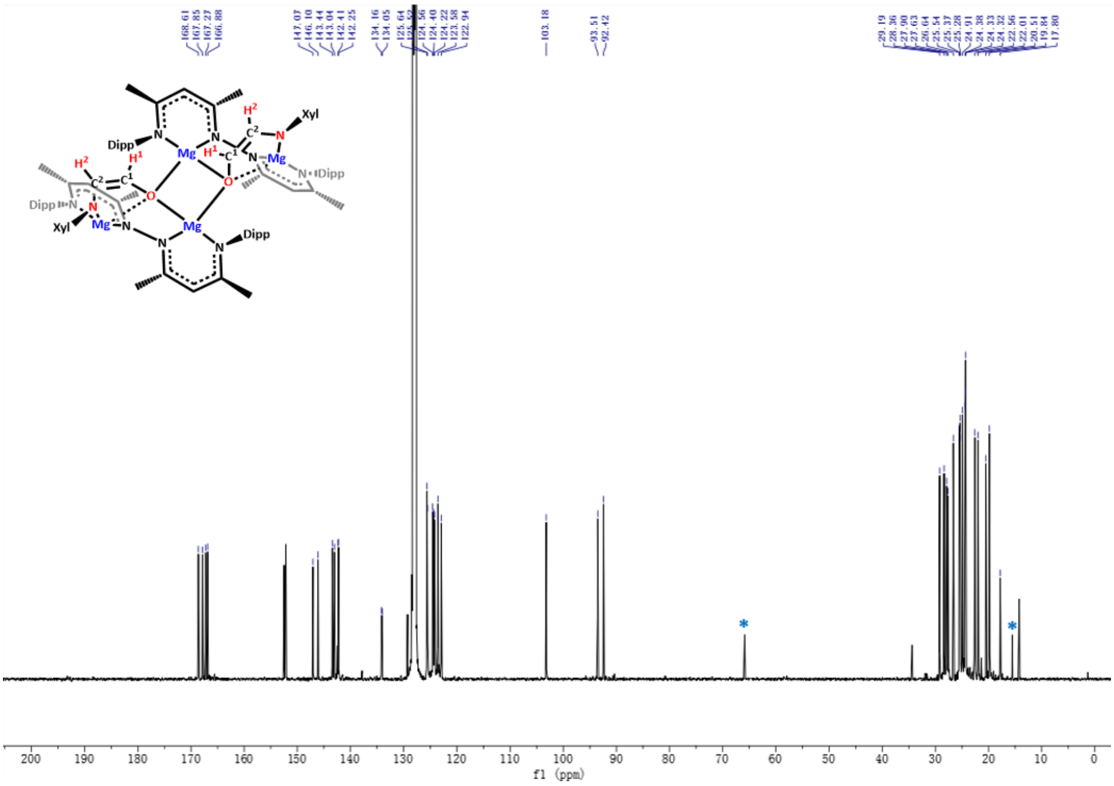

^{1}H NMR (d_8-THF, 400 MHz) of **3-THF$_2$**:

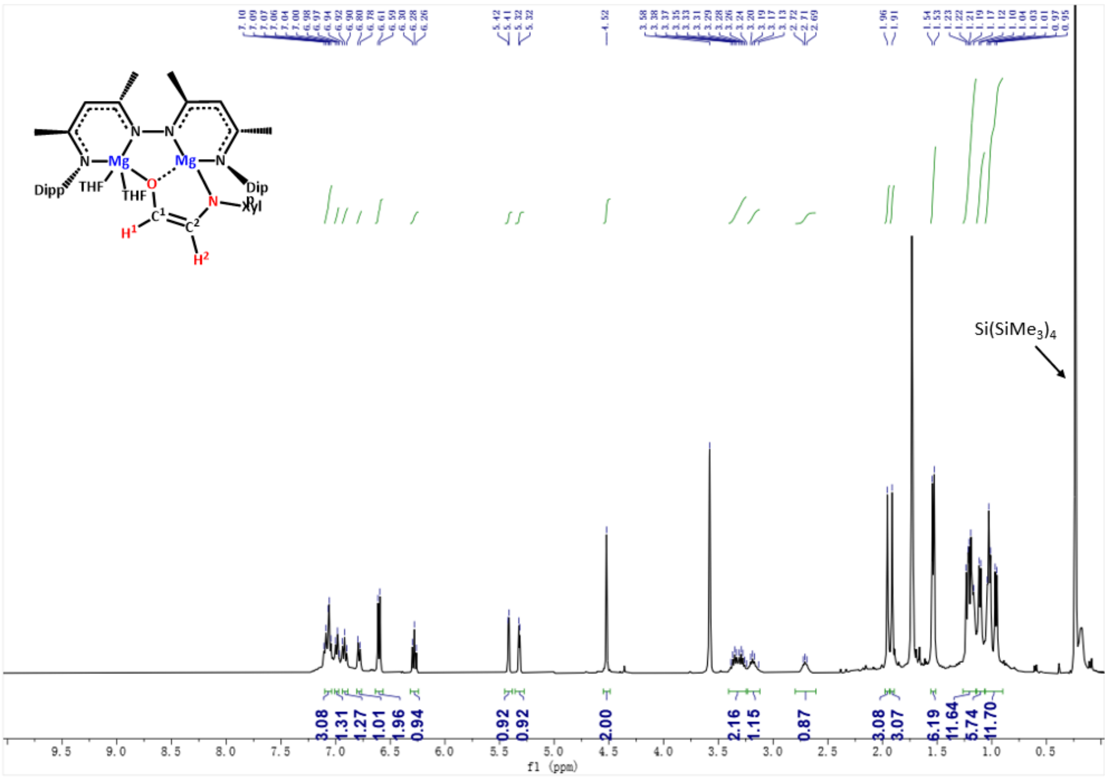

^{13}C NMR (d_8-THF, 101 MHz) of **3-THF$_2$**:

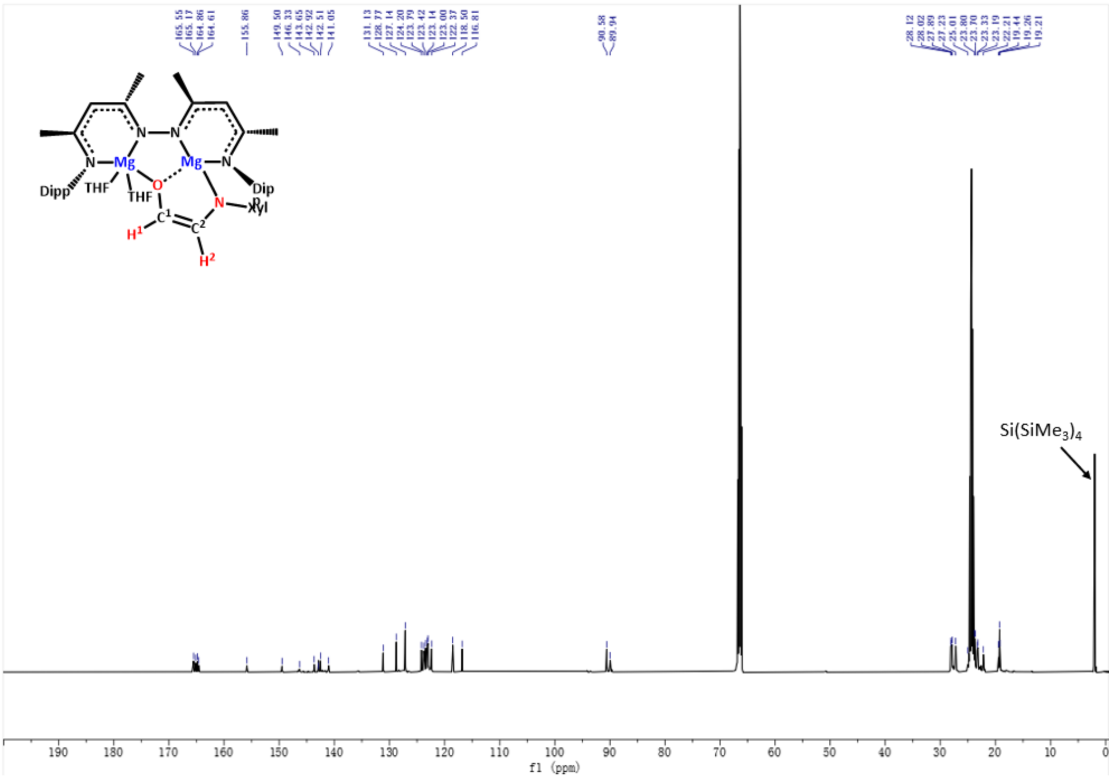

^{1}H NMR (C_6D_6, 400 MHz) of **2c**: "*"is the peak of diethyl ether.

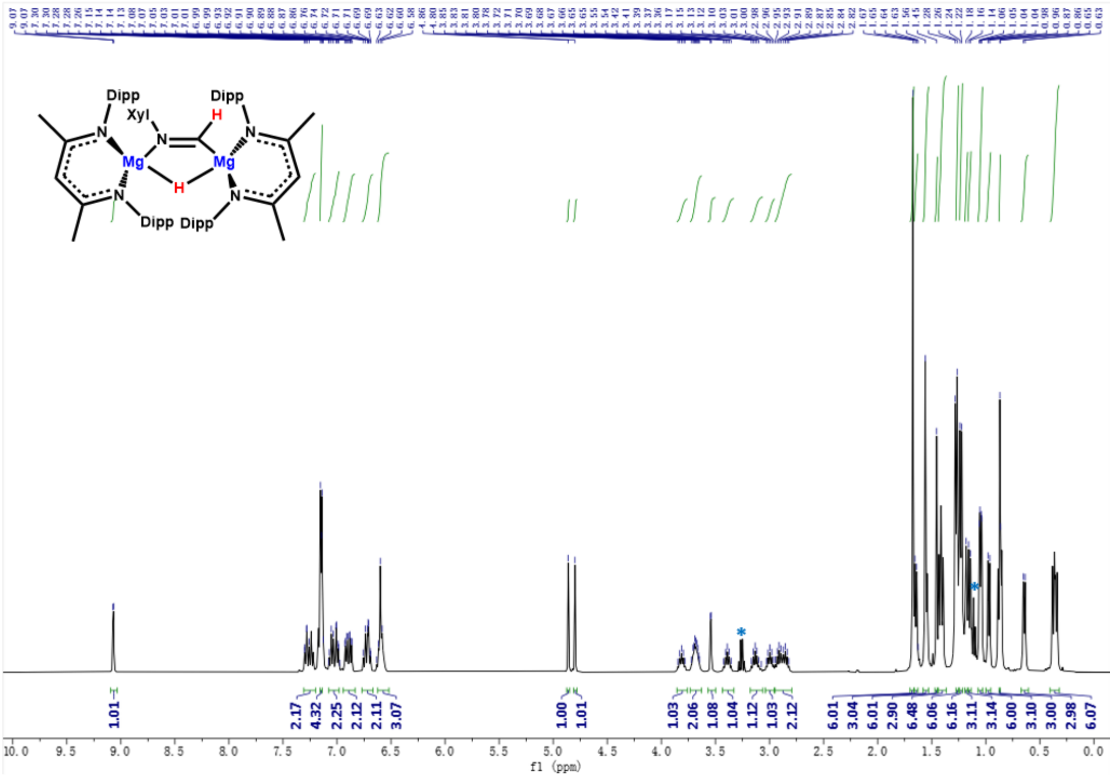

^{13}C NMR (C_6D_6, 101 MHz) of **2c**: "*"is the peak of diethyl ether.

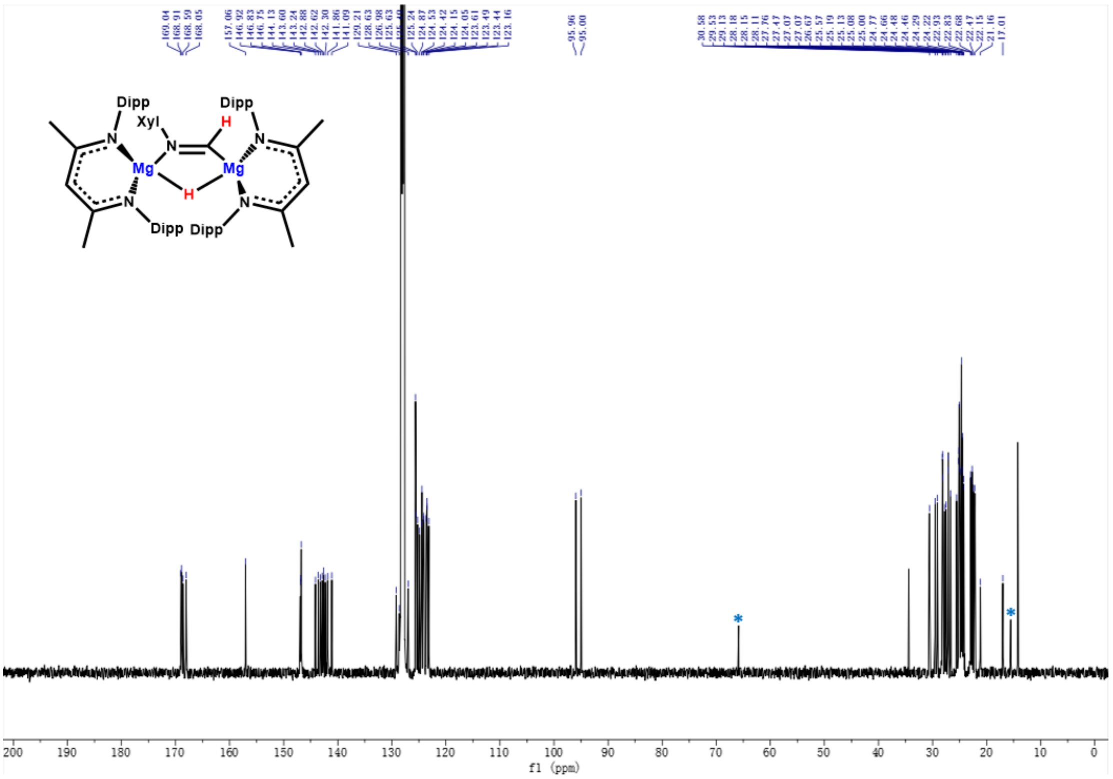

^{1}H NMR (C_6D_6, 400 MHz) of **4**: “*”is the peak of diethyl ether.

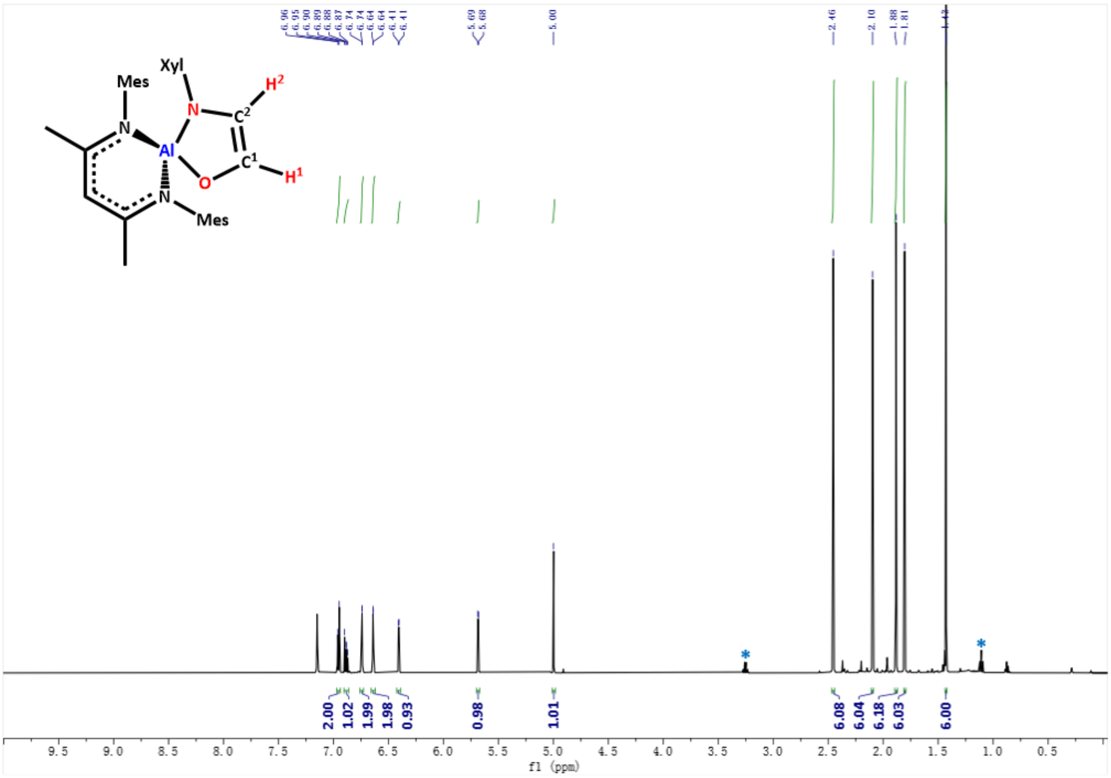

^{13}C NMR (C_6D_6, 101 MHz) of **4**: “*”is the peak of diethyl ether.

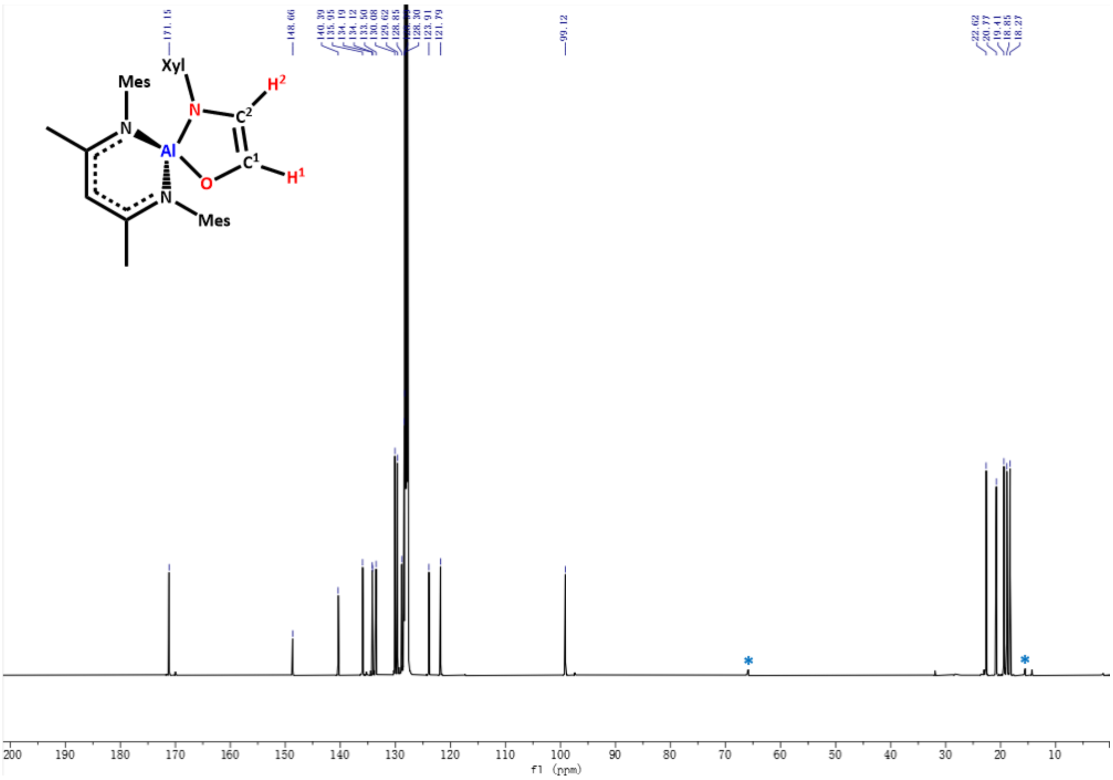

5.2. NMR Spectra for Determining the NMR Yields of 5

^{1}H NMR (C_6D_6, 400 MHz) of **5**: "*"is the peak of **5**, "*"is the peak of $^{Mes}BDIAlF_2$, "*"is the peak of BF_3-Et_2O.

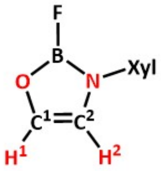

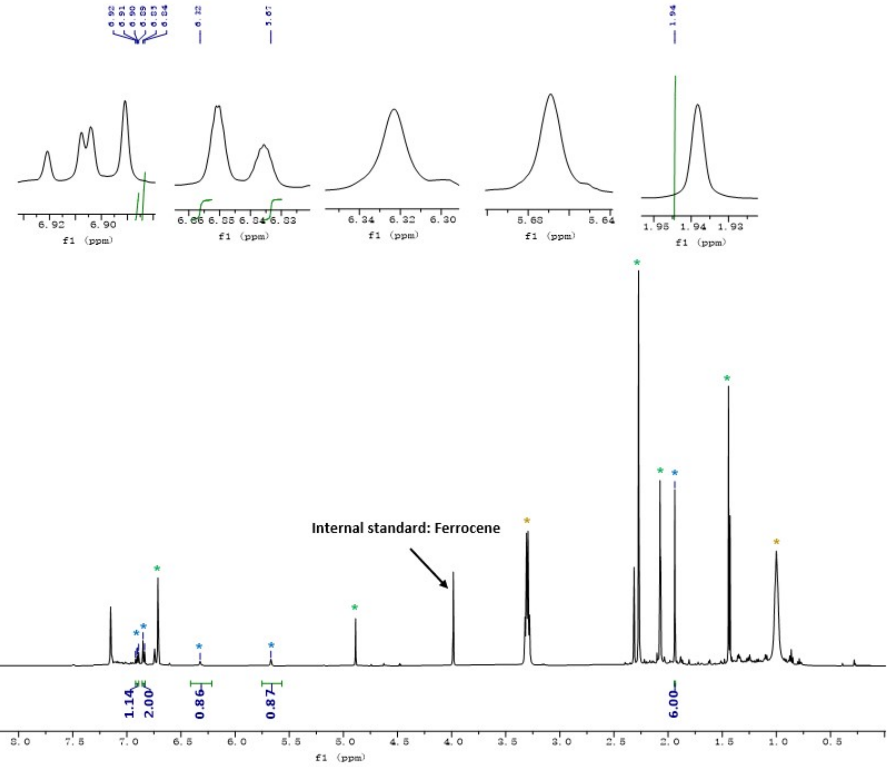

^{11}B NMR (C_6D_6, 128.0MHz) of **5**:

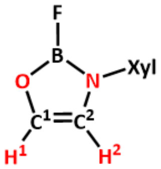

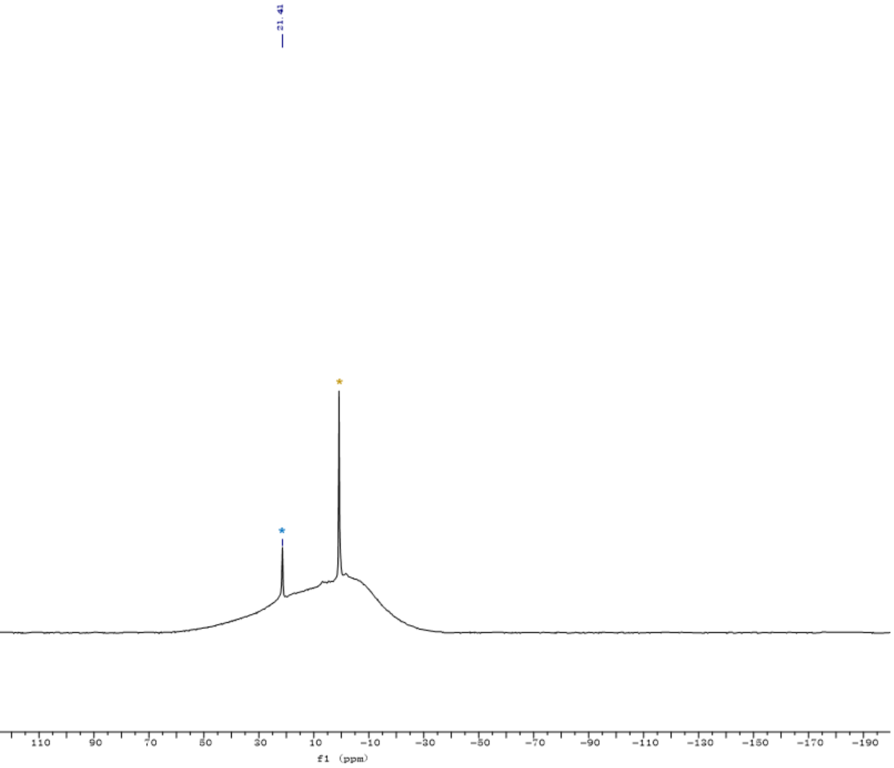

^{19}F NMR (C_6D_6, 376.5 MHz) of **5**:

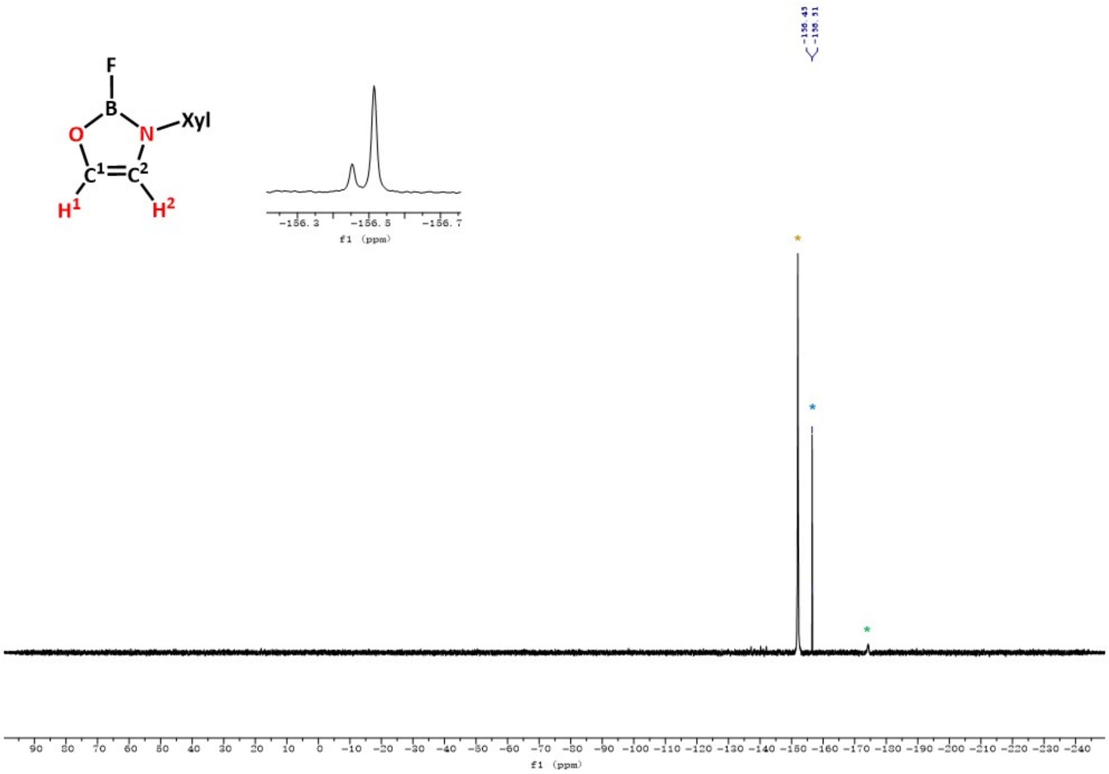

6) IR Spectroscopy

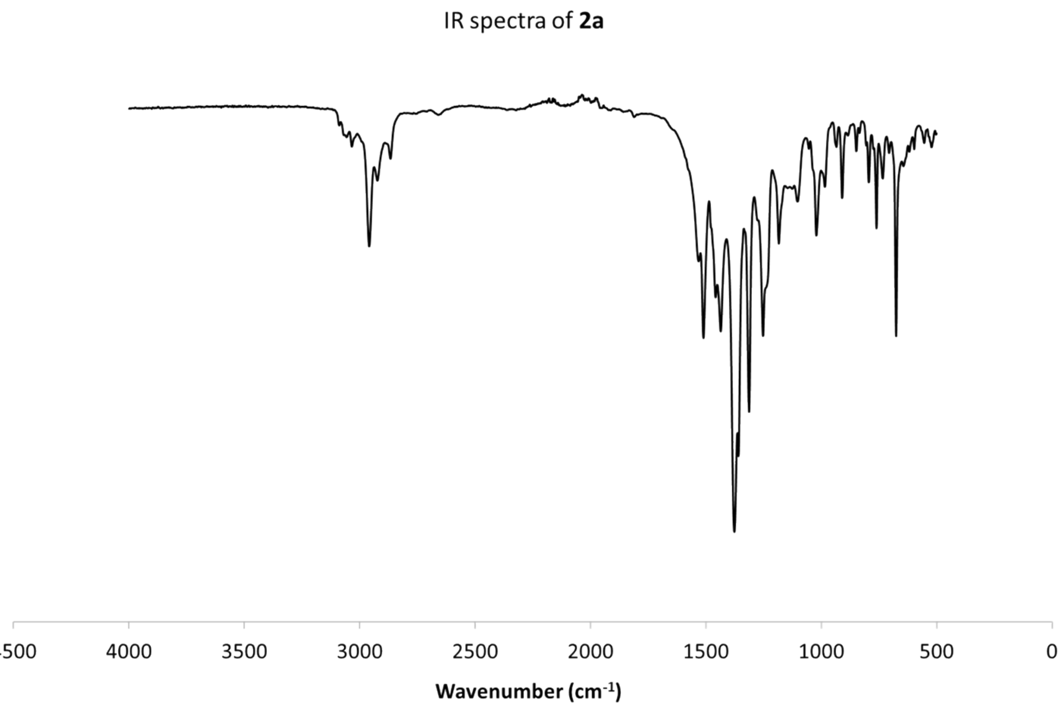

Figure S24. Infrared spectra of **2a**

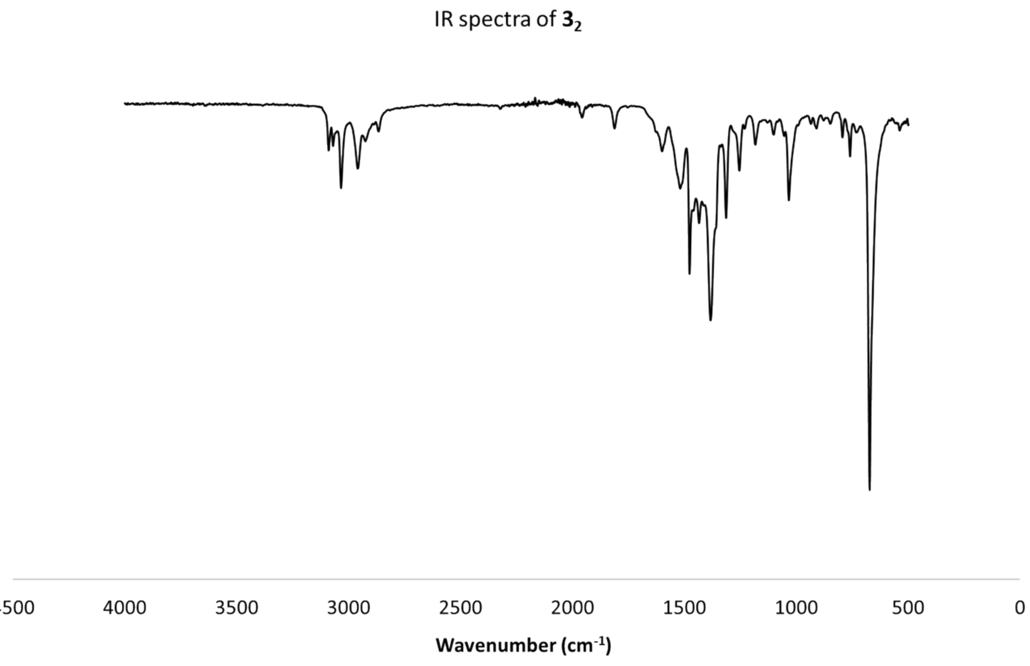

Figure S25. Infrared spectra of $\mathbf{3_2}$

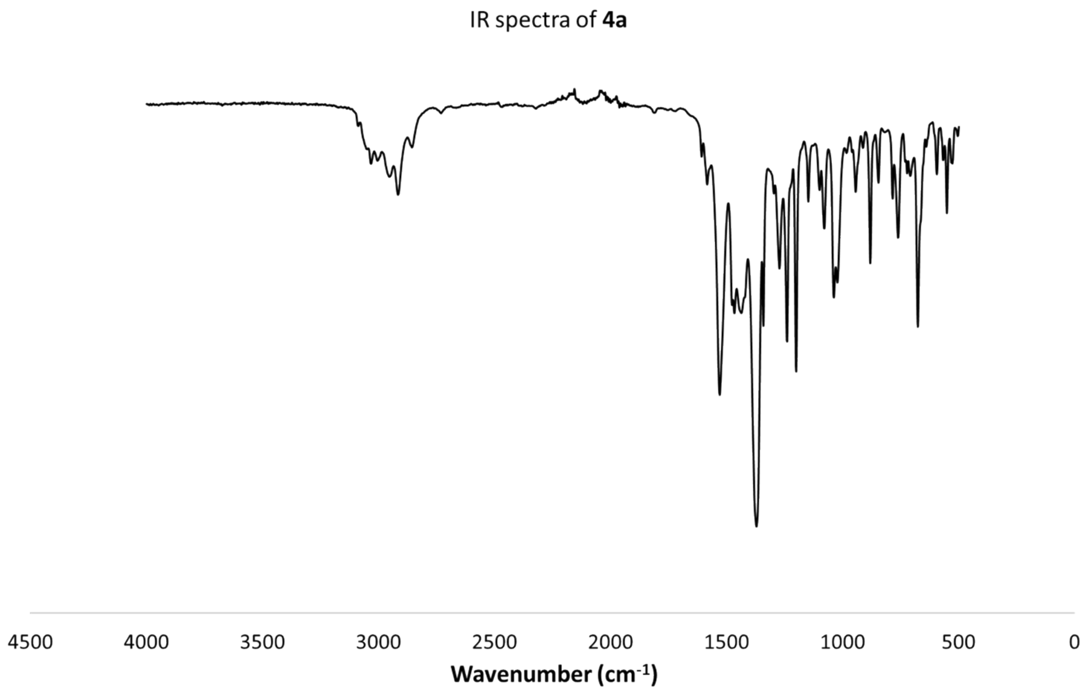

Figure S26. Infrared spectra of **4**

6) XYZ Coordinates

2.log

SCF (B3PW91) = -3898.52251869

E(SCF)+ZPE(0 K)= -3896.649681

H(298 K)= -3896.540365

G(298 K)= -3896.787348

Lowest Frequency = 20.6968cm-1

Mg	3.738540	10.492368	14.028706
Mg	5.776132	11.609547	10.771541
Mg	4.287351	8.181988	11.346675
Mg	2.263962	10.940418	10.632578
H	3.593521	9.242791	12.713278
H	4.097608	11.031493	10.331597
N	4.446638	9.802103	15.835057
C	5.741874	9.605746	16.107395
C	6.794985	10.141506	15.350637
H	7.796549	9.878340	15.668942
C	6.713107	11.070024	14.288282
N	5.561662	11.405171	13.721910
C	6.114102	8.789813	17.326292
H	5.757587	9.274304	18.240624
H	7.193857	8.651824	17.400598
H	5.633813	7.807223	17.295130
C	7.988184	11.720971	13.808796
H	8.102862	11.612021	12.725649
H	8.860283	11.288313	14.299835
H	7.972714	12.797078	14.008849
N	5.631901	12.358012	12.681861
C	5.445766	13.623795	13.030416
C	5.492676	14.681831	12.096692
H	5.243814	15.661809	12.486292
C	5.901655	14.638802	10.755341
N	6.210764	13.518919	10.089145
C	5.200980	13.965136	14.480947
H	4.356862	13.395777	14.881900
H	4.995928	15.029078	14.605122
H	6.067120	13.700086	15.095454
C	6.029206	15.969830	10.047420
H	7.077869	16.279524	9.995941
H	5.471370	16.748135	10.571226
H	5.669902	15.903670	9.016330
C	3.485046	9.447510	16.821063
C	3.049799	10.454270	17.717110
C	2.067037	10.123055	18.652722
H	1.711686	10.876960	19.347808
C	1.511167	8.846267	18.693597
H	0.745487	8.611158	19.428156
C	1.913351	7.884364	17.774345
H	1.442603	6.904949	17.784814
C	2.895442	8.165614	16.818790
C	3.598584	11.871608	17.606121
H	3.713665	12.066128	16.531339
C	2.654030	12.935846	18.165539
H	1.641204	12.823306	17.776105
H	3.013408	13.930741	17.884150
H	2.613340	12.904216	19.260942
C	4.987720	12.035490	18.241493
H	4.972562	11.715482	19.289724
H	5.287209	13.089379	18.216370
H	5.754153	11.467841	17.712078
C	3.244183	7.142502	15.750053
H	4.150376	7.490803	15.245729
C	3.526258	5.754150	16.331077
H	4.334932	5.781446	17.068979
H	3.813163	5.061855	15.534291
H	2.642627	5.332507	16.821859
C	2.142327	7.065456	14.682352
H	1.185376	6.768535	15.127055
H	2.402287	6.340627	13.902705
H	1.998957	8.026845	14.180674
C	6.865229	13.637894	8.829230
C	8.257570	13.893207	8.795363
C	8.913252	13.854377	7.562723
H	9.986552	14.021867	7.527113
C	8.220137	13.605979	6.381493
H	8.748934	13.581719	5.432440
C	6.847691	13.392897	6.423376
H	6.308270	13.197968	5.500106
C	6.149327	13.395566	7.635625
C	9.047107	14.198000	10.060535
H	8.332423	14.361700	10.872295
C	9.889030	15.473463	9.917960
H	10.709461	15.337141	9.204865
H	10.335744	15.741844	10.881298
H	9.289690	16.321602	9.571507

C	9.930337	13.016864	10.475000
H	9.337898	12.115598	10.644905
H	10.474944	13.248780	11.397257
H	10.662402	12.776535	9.697696
C	4.656008	13.110657	7.640272
H	4.298555	13.241424	8.667607
C	4.346656	11.664582	7.228920
H	4.700998	11.459339	6.211946
H	3.268075	11.474145	7.263273
H	4.818010	10.942666	7.901791
C	3.902328	14.099690	6.744091
H	4.075887	15.133166	7.060886
H	2.825887	13.906691	6.775546
H	4.218910	14.015195	5.699025
N	4.442686	6.110350	11.327535
C	3.387193	5.292038	11.347155
C	2.069614	5.742842	11.180787
H	1.283609	5.015798	11.354037
C	1.641095	6.981813	10.641107
N	2.479058	7.974380	10.359318
C	3.615520	3.808277	11.510782
H	4.127680	3.412440	10.626266
H	2.676810	3.265340	11.635545
H	4.265695	3.602677	12.367314
C	0.163232	7.121530	10.363968
H	-0.133910	8.170454	10.361029
H	-0.409666	6.581116	11.121383
H	-0.102290	6.698838	9.389141
N	1.973460	9.111537	9.705174
C	1.688836	9.021303	8.410053
C	1.044757	10.059690	7.691636
H	0.869015	9.858617	6.640494
C	0.465936	11.227186	8.211280
N	0.725807	11.683731	9.439336
C	2.024820	7.768286	7.637879
H	2.880933	7.259731	8.082745
H	2.247476	8.018729	6.598048
H	1.188209	7.061718	7.634216
C	-0.524753	11.973345	7.349791
H	-1.529834	11.887604	7.778536
H	-0.551525	11.581803	6.331212
H	-0.287546	13.041894	7.318236
C	5.751990	5.588960	11.137620
C	6.231291	5.434969	9.816290
C	7.583807	5.144926	9.619530
H	7.967775	5.054016	8.606639
C	8.453045	4.996901	10.696340
H	9.506113	4.791442	10.525604
C	7.961407	5.108684	11.992557
H	8.638159	4.979885	12.833636
C	6.613471	5.393356	12.234908
C	5.323529	5.657178	8.618000
H	4.297927	5.740364	8.991060
C	5.667076	6.980567	7.920774
H	6.717092	6.997755	7.608956
H	5.505092	7.829698	8.593596
H	5.045339	7.133250	7.032006
C	5.365719	4.484576	7.631928
H	6.349079	4.384382	7.159502
H	4.631239	4.633239	6.832879
H	5.138353	3.538003	8.132833
C	6.108710	5.521328	13.658613
H	5.016091	5.591160	13.611030
C	6.473177	4.295865	14.505266
H	6.110832	3.370683	14.045321
H	6.036468	4.376160	15.505520
H	7.557242	4.201457	14.630187
C	6.619831	6.807578	14.310849
H	7.713989	6.831407	14.337284
H	6.263160	6.885203	15.340497
H	6.283287	7.699496	13.772699
C	-0.133321	12.644803	10.041511
C	-1.272757	12.191980	10.744985
C	-1.980871	13.100518	11.536012
H	-2.836597	12.754153	12.109501
C	-1.592565	14.433847	11.627361
H	-2.141762	15.121182	12.264615
C	-0.499129	14.878953	10.892211
H	-0.204308	15.923961	10.949326
C	0.236486	14.003195	10.087557
C	-1.675994	10.727380	10.731613
H	-1.118897	10.234484	9.928585
C	-3.168891	10.530451	10.447501
H	-3.794782	10.920820	11.257347
H	-3.397031	9.464161	10.343502
H	-3.466210	11.035238	9.522575
C	-1.268528	10.059154	12.051168
H	-0.179867	10.072785	12.171172

H	-1.597048	9.014958	12.087752
H	-1.704401	10.586326	12.906571
C	1.430129	14.507825	9.304727
H	1.753287	13.689154	8.653348
C	2.603743	14.844605	10.226401
H	2.341081	15.638855	10.933214
H	3.460985	15.187753	9.642420
H	2.928561	13.979620	10.813676
C	1.065073	15.705620	8.420154
H	0.226763	15.469527	7.756580
H	1.917946	16.001283	7.801816
H	0.778089	16.575752	9.020547
C	1.678694	11.810692	12.555948
H	0.809573	12.485942	12.427100
C	6.333388	8.750411	10.810879
H	6.998671	7.867351	10.735418
N	6.967234	9.853878	10.524729
C	8.362650	9.757996	10.194083
C	9.307776	9.354082	11.156348
C	10.661899	9.373014	10.798253
H	11.402106	9.087106	11.541959
C	11.067832	9.723045	9.516093
H	12.123907	9.731249	9.260830
C	10.111233	10.038378	8.554589
H	10.414391	10.276589	7.538220
C	8.753962	10.059225	8.875823
C	8.919715	8.825671	12.509551
H	7.961010	9.204363	12.864055
H	9.680369	9.063114	13.258914
H	8.825386	7.735076	12.462753
C	7.719298	10.340506	7.830262
H	7.027638	9.499394	7.722429
H	8.177311	10.545442	6.860794
H	7.120914	11.215276	8.087533
N	1.995255	11.677550	13.815070
C	1.173231	12.382555	14.762347
C	0.321547	11.622183	15.586909
C	0.294977	14.417008	15.705802
C	-0.537213	12.290566	16.460855
C	-0.546723	13.682455	16.531663
H	0.279956	15.503801	15.744544
H	-1.203780	11.707172	17.090944
H	-1.210761	14.189919	17.225856
C	0.297203	10.126846	15.480312
H	0.074315	9.802433	14.460277
H	1.263717	9.698978	15.750741
H	-0.445476	9.693981	16.153754
C	1.151418	13.788854	14.792061
C	1.932441	14.642307	13.828283
H	2.329659	15.532274	14.326023
H	2.760623	14.114590	13.355952
H	1.274347	14.977168	13.018906

3-dimer.log

SCF (B3PW91) = -4125.29084310
E(SCF)+ZPE(0 K)= -4123.388065
H(298 K)= -4123.276083
G(298 K)= -4123.530185
Lowest Frequency = 7.0658cm-1

Mg	12.559452	7.866881	8.281883
Mg	11.413106	9.672067	5.690631
N	14.221088	6.704570	8.599432
C	15.429058	7.047592	8.140987
C	15.739040	8.273378	7.518079
H	16.760631	8.361007	7.165273
C	14.893390	9.344859	7.166978
N	13.621961	9.405284	7.552413
C	16.583747	6.067656	8.213986
H	16.405873	5.263931	8.928930
H	17.511620	6.580324	8.479856
H	16.733945	5.618335	7.226962
C	15.436379	10.409824	6.248978
H	14.877010	10.385041	5.306838
H	16.493610	10.247770	6.035141
H	15.304954	11.416139	6.655574
N	12.809609	10.408400	6.978392
C	12.720325	11.577469	7.592405
C	11.971035	12.643379	7.043818
H	11.981942	13.562995	7.614355
C	11.292010	12.676086	5.818795
N	11.059203	11.594183	5.056374
C	13.430824	11.788268	8.901467
H	13.075822	11.047678	9.627112
H	13.233111	12.783199	9.299871
H	14.511031	11.642623	8.813349
C	10.817498	14.030575	5.338908

H	11.243153	14.261185	4.357433	H	8.660667	9.703481	4.840213
H	11.102203	14.820578	6.035649	H	7.329973	9.969276	3.716688
H	9.729481	14.046459	5.215881	C	7.020546	12.710378	4.202597
C	14.046343	5.454969	9.272302	H	6.425161	12.379403	3.344710
C	14.162961	5.429699	10.683902	H	7.320498	13.747950	4.023215
C	13.983388	4.213227	11.346320	H	6.363656	12.689775	5.078512
H	14.072785	4.173345	12.426653	O	11.310144	8.151953	9.831033
C	13.679104	3.047227	10.649748	C	11.028696	6.919018	10.483236
H	13.547761	2.110592	11.184606	H	11.417058	6.016422	10.021274
C	13.518041	3.095333	9.272352	C	10.586435	6.940844	11.770772
H	13.252945	2.190425	8.732602	H	10.386061	5.983858	12.254386
C	14.465248	6.700158	11.462967	N	10.403923	8.093081	12.470848
H	13.939472	7.517819	10.950566	C	9.771688	7.961916	13.730966
C	13.958855	6.654565	12.904017	C	8.416812	7.556434	13.795595
H	12.897852	6.399011	12.952353	C	7.805493	7.394134	15.041017
H	14.087539	7.634770	13.365760	H	6.763236	7.084153	15.080857
H	14.523741	5.938738	13.511976	C	8.507225	7.630024	16.220370
C	15.963662	7.040402	11.462269	H	8.021228	7.501616	17.183820
H	16.548798	6.193079	11.837050	C	9.830312	8.055099	16.151402
H	16.156138	7.899599	12.113084	H	10.383262	8.258824	17.066094
H	16.326774	7.295331	10.466413	C	10.476673	8.232432	14.922738
C	10.664134	11.801849	3.701215	C	7.642025	7.307993	12.531116
C	11.672618	11.819869	2.708264	H	7.770840	6.283459	12.162859
C	11.284921	12.027237	1.381294	H	7.985783	7.960365	11.726353
H	12.038173	12.050328	0.600468	H	6.570719	7.470958	12.679234
C	9.944826	12.182271	1.036080	C	11.913652	8.668319	14.910214
H	9.666347	12.339634	-0.002504	H	12.088597	9.459305	15.643222
C	8.961714	12.112912	2.018322	H	12.206759	9.046636	13.931941
H	7.917658	12.213122	1.736445	H	12.575905	7.829478	15.152014
C	9.301275	11.922977	3.360436	Mg	9.587472	7.942588	8.638024
C	13.135623	11.602895	3.077649	Mg	10.763109	9.665120	11.244381
H	13.149285	10.832953	3.860638	N	7.935567	6.756365	8.387764
C	13.788007	12.861507	3.669938	C	6.727248	7.109007	8.841077
H	13.706879	13.700282	2.969092	C	6.422041	8.337635	9.460261
H	14.852641	12.679837	3.855369	H	5.400686	8.430499	9.812825
H	13.337258	13.153123	4.619603	C	7.273075	9.407319	9.803840
C	13.990236	11.080180	1.922226	N	8.537981	9.461919	9.399650
H	13.533765	10.213839	1.440646	C	5.561794	6.142066	8.757282
H	14.970078	10.773546	2.301672	H	5.759646	5.304149	8.088489
H	14.164033	11.854007	1.165034	H	4.657573	6.656633	8.421697
C	8.236786	11.812191	4.437740	H	5.352100	5.743129	9.754806
H	8.698891	12.127295	5.375909	C	6.747911	10.474912	10.727948
C	7.805598	10.352948	4.625731	H	7.308482	10.436607	11.668933
H	7.098273	10.251160	5.455793	H	5.688812	10.327976	10.943608

H	6.895221	11.480651	10.325272	C	11.570519	11.786361	13.228235
N	9.375495	10.445197	9.971684	C	10.567864	11.822893	14.226222
C	9.487009	11.612717	9.355499	C	10.965560	12.030568	15.550171
C	10.263383	12.660666	9.896641	H	10.216821	12.066739	16.334803
H	10.268714	13.580883	9.326997	C	12.309086	12.171157	15.887293
C	10.949448	12.676097	11.118931	H	12.595107	12.330509	16.923544
N	11.164035	11.587105	11.874617	C	13.285951	12.083793	14.900344
C	8.770515	11.837600	8.051814	H	14.332661	12.172031	15.176115
H	9.093906	11.081465	7.327480	C	12.936739	11.889939	13.561436
H	8.995263	12.823474	7.645129	C	9.099508	11.629566	13.865265
H	7.687183	11.726634	8.152933	H	9.068941	10.857262	13.085255
C	11.449538	14.020786	11.600769	C	8.465248	12.897505	13.273181
H	11.028928	14.257174	12.583025	H	8.563849	13.736242	13.971886
H	11.178170	14.816893	10.905637	H	7.396732	12.733271	13.093807
H	12.537696	14.017121	11.722832	H	8.915632	13.180178	12.320632
C	8.107495	5.503472	7.719618	C	8.242424	11.125349	15.026813
C	8.003050	5.475306	6.306885	H	8.687009	10.253424	15.509036
C	8.174462	4.254755	5.649467	H	7.255570	10.834000	14.653527
H	8.092785	4.212246	4.568614	H	8.085621	11.904919	15.781904
C	8.459550	3.087861	6.352278	C	13.992671	11.755501	12.478814
H	8.583658	2.147867	5.821636	H	13.535602	12.089826	11.545111
C	8.612990	3.139109	7.730531	C	14.379377	10.285277	12.281143
H	8.866348	2.233525	8.274459	H	15.072598	10.165111	11.441796
C	8.460935	4.338626	8.436289	H	13.503424	9.659790	12.078821
C	7.721101	6.746034	5.520190	H	14.856313	9.887384	13.183198
H	8.243372	7.563299	6.036852	C	15.236281	12.615646	12.711733
C	8.250836	6.694708	4.087558	H	15.826051	12.262734	13.564768
H	9.312768	6.439100	4.056329	H	14.968735	13.660993	12.897361
H	8.128005	7.672647	3.619254	H	15.887618	12.579350	11.832184
H	7.696751	5.976287	3.472980	O	10.802049	8.213580	7.081462
C	6.225251	7.094942	5.498049	C	11.166047	6.954910	6.522378
H	5.641503	6.252821	5.109727	H	10.695336	6.069332	6.944236
H	6.047936	7.958951	4.849229	C	11.653273	6.938019	5.240765
H	5.848693	7.345721	6.489878	H	11.890378	5.968698	4.800245
C	8.686686	4.347756	9.937662	N	11.836019	8.062641	4.515318
H	8.760629	5.395334	10.247606	C	12.462986	7.909927	3.253689
C	7.520940	3.699356	10.695486	C	13.809059	7.478950	3.183921
H	6.573191	4.199139	10.485675	C	14.408977	7.305604	1.934377
H	7.412632	2.646150	10.411515	H	15.445168	6.977040	1.888652
H	7.695145	3.739461	11.776679	C	13.705039	7.553438	0.758952
C	10.002174	3.653354	10.316238	H	14.183295	7.415550	-0.206969
H	10.847440	4.010320	9.722379	C	12.390273	8.001803	0.834779
H	10.237002	3.821992	11.372079	H	11.835647	8.215020	-0.076626
H	9.944507	2.569057	10.171101	C	11.754888	8.192547	2.066907

C	14.590864	7.216517	4.441723
H	14.464534	6.187834	4.799723
H	14.258576	7.862677	5.255588
H	15.661274	7.378272	4.286025
C	10.327552	8.660064	2.085330
H	10.177776	9.487295	1.387424
H	10.031037	9.000556	3.076157
H	9.648946	7.848357	1.800015
C	13.679439	4.291038	8.562037
C	13.447652	4.295770	7.061650
H	13.424268	5.342433	6.741566
C	12.097349	3.661686	6.701096
H	11.273971	4.081018	7.284443
H	11.869031	3.808776	5.640442
H	12.097175	2.581166	6.880714
C	14.576637	3.583726	6.305281
H	15.550497	4.030808	6.516382
H	14.627665	2.526334	6.589432
H	14.406079	3.632064	5.223778

3-monomer.log

SCF (B3PW91) = -2062.59140462
E(SCF)+ZPE(0 K)= -2061.640764
H(298 K)= -2061.585413
G(298 K)= -2061.723944
Lowest Frequency = 22.5938cm-1

Mg	5.586324	3.304449	8.348277
Mg	5.754440	3.541126	5.607684
N	6.083803	3.337401	10.279281
C	7.309542	2.990421	10.694849
C	8.406515	2.829146	9.833571
H	9.308377	2.434136	10.289203
C	8.524994	3.135001	8.448982
N	7.528797	3.696134	7.774804
C	7.518869	2.701272	12.163121
H	7.028282	1.759291	12.432399
H	7.061171	3.478904	12.781455
H	8.578007	2.624948	12.414542
C	9.828947	2.769002	7.784347
H	9.731110	2.750806	6.697274
H	10.149386	1.782327	8.128612
H	10.618404	3.479582	8.049606
N	7.588011	3.986081	6.402905
C	8.360992	4.940297	5.903832
C	8.257264	5.344256	4.544112
H	9.034618	6.027788	4.220154
C	7.277808	5.055759	3.582616
N	6.156806	4.349749	3.816443
C	9.349217	5.693928	6.757793
H	9.082946	5.634115	7.815354
H	9.370072	6.743717	6.456838
H	10.361751	5.294983	6.637498
C	7.509472	5.602751	2.191717
H	8.314038	6.339771	2.180752
H	6.601567	6.056542	1.785131
H	7.783356	4.789574	1.510572
C	5.020843	3.609722	11.183205
C	4.716569	4.962656	11.468938
C	3.616403	5.236374	12.284952
H	3.367763	6.266108	12.521356
C	2.814088	4.211975	12.781709
H	1.960348	4.446992	13.411698
C	3.090791	2.891796	12.445762
H	2.438100	2.099553	12.803290
C	4.186882	2.567519	11.639837
C	5.554843	6.080619	10.867732
H	5.866428	5.739626	9.871612
C	4.780275	7.385254	10.678954
H	3.836106	7.218747	10.154315
H	5.372307	8.083589	10.079791
H	4.568633	7.875711	11.636423
C	6.838186	6.327918	11.673382
H	6.596904	6.587042	12.710648
H	7.406294	7.158725	11.239918
H	7.487903	5.449941	11.680598
C	4.412549	1.132141	11.197192
H	5.394859	1.083216	10.715026
C	4.417786	0.143097	12.368209
H	5.137571	0.439498	13.137776
H	4.682702	-0.860563	12.019323
H	3.433348	0.075086	12.843794
C	3.364828	0.728530	10.147165
H	3.377467	1.393449	9.275690
H	2.355617	0.768683	10.572007
H	3.545031	-0.290791	9.788913
C	5.214587	4.143060	2.764898

C	5.430700	3.115182	1.821187
C	4.474173	2.910120	0.822644
H	4.626959	2.122951	0.089373
C	3.322701	3.687646	0.761182
H	2.592325	3.519750	-0.025825
C	3.096011	4.664002	1.726936
H	2.179691	5.245326	1.690487
C	4.019547	4.900961	2.749022
C	6.626537	2.184955	1.941740
H	7.365469	2.667888	2.588415
C	7.306031	1.889380	0.600903
H	7.569723	2.811842	0.073531
H	6.663273	1.299521	-0.061506
H	8.223115	1.312582	0.760095
C	6.199775	0.882500	2.637117
H	5.751799	1.078232	3.617637
H	7.057749	0.217071	2.784139
H	5.453557	0.351216	2.035635
C	3.746707	5.938765	3.825389
H	4.198395	5.567580	4.750363
C	2.261878	6.148544	4.130757
H	1.749175	6.692025	3.328760
H	2.160271	6.735427	5.049184
H	1.743654	5.195252	4.275900
C	4.421776	7.280148	3.514797
H	5.506031	7.173073	3.429628
H	4.216706	7.996747	4.317304
H	4.042140	7.693943	2.573688
O	5.249462	1.980454	6.838856
C	3.975961	2.182207	6.362441
H	3.465128	1.362684	5.865407
C	3.468081	3.436337	6.501641
H	2.503890	3.728747	6.099402
N	4.310894	4.395028	7.110471
C	3.714855	5.632902	7.436903
C	4.404563	6.834156	7.155952
C	3.756837	8.055064	7.349600
H	4.285350	8.972636	7.101092
C	2.462911	8.114286	7.862525
H	1.966216	9.071053	7.995461
C	1.846437	6.935689	8.272264
H	0.877531	6.974338	8.765429
C	2.465724	5.692913	8.102714
C	5.830878	6.795851	6.692812
H	6.447475	6.215606	7.388351
H	6.250400	7.802910	6.625479
H	5.946002	6.328942	5.709899
C	1.860041	4.466211	8.737398
H	1.314960	3.822402	8.039374
H	1.168886	4.755889	9.533414
H	2.642450	3.848439	9.195307

3-THF.log

SCF (B3PW91) = -2527.44192456
E(SCF)+ZPE(0 K)= -2526.251995
H(298 K)= -2526.184255
G(298 K)= -2526.350508
Lowest Frequency = 16.1904cm-1

Mg	5.438766	4.262309	8.179553
Mg	5.831814	2.714841	5.238679
N	5.993693	3.617857	10.055977
C	7.134721	2.993419	10.365324
C	8.196331	2.784679	9.466178
H	9.053693	2.251002	9.860406
C	8.266622	3.123100	8.093078
N	7.320655	3.825996	7.494036
C	7.302474	2.409649	11.752127
H	6.674284	1.518394	11.860305
H	6.977878	3.114285	12.522015
H	8.338477	2.124457	11.943969
C	9.438930	2.614335	7.292946
H	9.079994	2.198405	6.348135
H	9.996834	1.854797	7.843782
H	10.127660	3.425969	7.035988
N	7.352436	3.935553	6.084685
C	7.914941	5.020384	5.586918
C	7.841995	5.332104	4.210063
H	8.419226	6.194248	3.897803
C	7.084584	4.712962	3.212325
N	6.252311	3.666902	3.392260
C	8.632767	5.988084	6.494300
H	7.951958	6.331606	7.278446
H	8.998501	6.854208	5.940839
H	9.476481	5.515486	7.005142
C	7.238337	5.307140	1.824976
H	7.739495	6.275387	1.867724

H	6.274984	5.431505	1.324621
H	7.837109	4.645550	1.190967
C	4.986072	3.754037	11.055418
C	4.858736	4.987690	11.735815
C	3.846076	5.115036	12.690380
H	3.728007	6.052322	13.223344
C	2.965329	4.070864	12.957181
H	2.182681	4.196171	13.700632
C	3.071494	2.881023	12.247660
H	2.358116	2.081815	12.430758
C	4.072266	2.703843	11.288079
C	5.783577	6.151117	11.407955
H	5.870974	6.172997	10.313005
C	5.226501	7.506815	11.841396
H	4.199325	7.648818	11.500160
H	5.831923	8.309316	11.409418
H	5.258317	7.623020	12.931777
C	7.197708	5.968506	11.979544
H	7.157131	5.825748	13.065649
H	7.800522	6.862155	11.781538
H	7.716980	5.116971	11.536964
C	4.115145	1.434657	10.456341
H	5.092452	1.399840	9.967277
C	3.970633	0.155072	11.285945
H	4.696836	0.122662	12.104578
H	4.128836	-0.725716	10.653500
H	2.971073	0.063881	11.724554
C	3.046661	1.498659	9.354761
H	3.182768	2.366947	8.701013
H	2.046324	1.571572	9.795802
H	3.073666	0.595029	8.732745
C	5.508903	3.257817	2.247709
C	6.091459	2.409276	1.278811
C	5.316326	1.991711	0.191655
H	5.752159	1.330697	-0.552841
C	3.992451	2.395124	0.057367
H	3.402357	2.061737	-0.791962
C	3.424614	3.225479	1.020322
H	2.389193	3.532086	0.908943
C	4.159044	3.669510	2.124189
C	7.503348	1.872259	1.445051
H	7.992647	2.458441	2.229002
C	8.348494	1.982625	0.171083
H	8.356512	3.003825	-0.221717
H	7.973365	1.326874	-0.622171
H	9.383285	1.687878	0.375023
C	7.449063	0.416321	1.927914
H	6.852717	0.333815	2.841048
H	8.453338	0.027834	2.131637
H	6.980973	-0.227498	1.174563
C	3.530338	4.581665	3.163639
H	3.956200	4.323555	4.139087
C	2.015572	4.416878	3.292634
H	1.478907	4.822620	2.427487
H	1.669343	4.956565	4.178989
H	1.732680	3.365172	3.405285
C	3.879813	6.057524	2.923901
H	4.953440	6.235894	3.010344
H	3.381233	6.684438	3.670819
H	3.551438	6.379749	1.928906
O	4.564714	3.243775	6.668159
C	3.304010	3.788523	6.500695
H	2.631390	3.298440	5.804038
C	2.964034	4.901537	7.190715
H	1.978428	5.342798	7.042322
N	3.871611	5.525389	8.034587
C	3.446567	6.677117	8.696410
C	4.197947	7.868433	8.542958
C	3.751986	9.051726	9.132170
H	4.326352	9.964618	8.986049
C	2.586457	9.079308	9.896861
H	2.245726	10.007625	10.347534
C	1.886727	7.892919	10.109046
H	1.007770	7.891947	10.751532
C	2.304284	6.687137	9.537250
C	5.467775	7.833662	7.740330
H	6.237375	7.245731	8.260812
H	5.876082	8.836690	7.583376
H	5.314183	7.349918	6.769764
C	1.600972	5.399881	9.873202
H	0.912592	5.068411	9.086971
H	1.028970	5.501026	10.799792
H	2.335457	4.599298	10.010104
O	4.344651	1.234281	4.532533
C	4.084652	0.482555	3.331074
H	4.568640	-0.500505	3.429639
H	4.520203	1.015885	2.492476
C	2.561935	0.355234	3.269226

H	2.150793	1.150358	2.643110
H	2.261106	-0.602603	2.836965
C	2.118868	0.514111	4.742228
H	1.542216	-0.338897	5.109122
H	1.505762	1.409806	4.872222
C	3.437164	0.662007	5.497973
H	3.407807	1.325173	6.358780
H	3.830665	-0.316903	5.802544
O	6.659590	1.047770	6.257881
C	7.424850	-0.063176	5.780341
H	8.399831	-0.073456	6.287360
H	7.582447	0.072300	4.709997
C	6.583178	-1.271787	6.158776
H	7.164222	-2.196614	6.193142
H	5.782258	-1.399169	5.423874
C	6.013346	-0.864195	7.532315
H	6.600689	-1.301395	8.344373
H	4.979921	-1.196019	7.662238
C	6.131691	0.669578	7.553792
H	5.189837	1.204683	7.665132
H	6.830078	1.020018	8.319928

4a.log

SCF (B3PW91) = -1522.85454433
E(SCF)+ZPE(0 K)= -1522.195879
H(298 K)= -1522.155645
G(298 K)= -1522.266143
Lowest Frequency = 20.4666cm-1

Al	3.290883	4.927952	5.651605
N	2.039059	3.668369	4.997871
C	2.099892	3.121448	3.775765
C	3.190164	3.300051	2.912742
H	3.128906	2.804437	1.951828
C	4.409504	3.929611	3.209532
N	4.611566	4.644819	4.324350
C	0.980558	2.219525	3.329468
H	0.996516	1.281735	3.894552
H	1.062554	1.984947	2.267300
H	0.010123	2.685103	3.524100
C	5.544267	3.763154	2.236173
H	5.752689	4.701645	1.715155
H	5.315293	2.999247	1.492368
H	6.459115	3.484937	2.767905
C	1.091307	3.137934	5.942417
C	1.429106	1.958848	6.630242
C	0.518552	1.444622	7.552907
H	0.772136	0.531311	8.087517
C	-0.698500	2.078742	7.820272
C	-0.989508	3.261604	7.139980
H	-1.926064	3.777433	7.341715
C	-0.107126	3.812161	6.205835
C	2.764288	1.302820	6.408869
H	2.941684	1.055874	5.356325
H	2.851214	0.382314	6.991306
H	3.565006	1.982668	6.720596
C	-1.647298	1.518052	8.846631
H	-2.666478	1.885506	8.695846
H	-1.340511	1.805793	9.859516
H	-1.671730	0.424152	8.814351
C	-0.413332	5.111040	5.518768
H	0.295328	5.881905	5.842005
H	-1.424570	5.455201	5.749298
H	-0.315250	5.029436	4.431163
C	5.882200	5.273290	4.568468
C	6.225208	6.470369	3.918693
C	7.418021	7.101350	4.284214
H	7.673056	8.042384	3.801155
C	8.268897	6.576970	5.254895
C	7.918805	5.362859	5.853065
H	8.580578	4.923807	6.596751
C	6.737574	4.696319	5.528416
C	5.373585	7.090328	2.846658
H	5.847991	6.978192	1.863775
H	5.247456	8.159616	3.030572
H	4.378266	6.647377	2.797945
C	9.512902	7.312870	5.675249
H	10.307352	6.622668	5.975180
H	9.304716	7.962497	6.534285
H	9.896994	7.947346	4.871065
C	6.386161	3.390662	6.189880
H	5.589014	3.530358	6.930588
H	7.254315	2.970135	6.703858
H	6.028488	2.654813	5.461860
O	3.507344	4.517531	7.378405
C	3.100534	5.607549	8.102786
H	2.996368	5.466762	9.173998

C	2.824302	6.748424	7.439790
H	2.495720	7.665694	7.918400
N	2.939083	6.711118	6.025615
C	3.308014	7.896681	5.358360
C	2.580108	8.313531	4.220927
C	2.917679	9.511265	3.587664
H	2.342928	9.828297	2.720203
C	3.969093	10.299454	4.049847
H	4.215663	11.234020	3.553432
C	4.720067	9.858034	5.136017
H	5.576506	10.438430	5.472792
C	4.423831	8.657665	5.788992
C	1.449530	7.476486	3.692194
H	1.755376	6.440628	3.499856
H	1.062379	7.887271	2.755369
H	0.627312	7.428827	4.411283
C	5.344744	8.149766	6.865682
H	4.918261	8.236498	7.869644
H	6.292716	8.694305	6.843456
H	5.562421	7.087661	6.710039

cis-enamidolate-dianion.log

SCF (B3PW91) = -517.528281725
E(SCF)+ZPE(0 K)= -517.345281
H(298 K)= -517.333251
G(298 K)= -517.381711
Lowest Frequency = 77.6416cm-1

O	9.924148	8.021993	8.213131
C	8.636485	7.910792	8.011309
H	8.161297	8.700971	7.372444
C	7.709913	6.996058	8.450255
H	6.679420	7.197122	8.117772
N	7.915779	5.791538	9.143667
C	6.981539	5.355258	9.992837
C	5.970506	6.154155	10.689606
C	4.971188	5.530988	11.432045
H	4.237672	6.169395	11.934936
C	4.897740	4.143544	11.614354
H	4.097540	3.687497	12.194115
C	5.945686	3.376197	11.074921
H	5.963958	2.296293	11.246407
C	6.966810	3.936471	10.325596
C	6.112568	7.654259	10.803529
H	5.947139	8.209378	9.873810
H	7.137178	7.915381	11.101027
H	5.427251	8.042174	11.571845
C	8.066478	3.078665	9.771399
H	7.957317	2.030777	10.084617
H	9.054177	3.440234	10.088044
H	8.090324	3.124429	8.673633

CN-Xyl.log

SCF (B3PW91) = -402.968707697
E(SCF)+ZPE(0 K)= -402.814294
H(298 K)= -402.803844
G(298 K)= -402.848557
Lowest Frequency = 110.6830cm-1

C	-0.618805	0.938834	0.000261
C	0.786939	0.900061	0.000663
C	1.460599	2.122266	-0.000022
C	0.759770	3.326319	-0.001049
C	-0.633372	3.331386	-0.001411
C	-1.355095	2.136941	-0.000765
H	2.546960	2.125273	0.000253
H	1.302927	4.266932	-0.001566
H	-1.173977	4.273700	-0.002206
C	-1.900171	-1.279428	0.001435
N	-1.311193	-0.260134	0.000901
C	-2.855847	2.110320	-0.001140
H	-3.240383	1.581538	-0.880300
H	-3.240845	1.582640	0.878479
H	-3.266251	3.122458	-0.001883
C	1.513997	-0.413113	0.001784
H	1.248450	-1.009775	0.881491
H	1.249044	-1.010951	-0.877305
H	2.595801	-0.262859	0.002052

CO.log

SCF (B3PW91) = -113.263433183
E(SCF)+ZPE(0 K)= -113.258397
H(298 K)= -113.255092
G(298 K)= -113.277533
Lowest Frequency = 2210.5326cm-1

C -3.727095 0.240642 0.000000
O -2.590660 0.240642 0.000000

INT-1.log

SCF (B3PW91) = -3898.53468373
E(SCF)+ZPE(0 K)= -3896.658673
H(298 K)= -3896.549830
G(298 K)= -3896.795899
Lowest Frequency = 14.2492cm-1

Mg 3.760901 10.498915 14.141688
Mg 5.544560 11.735419 10.985032
Mg 4.545326 8.439729 11.075395
Mg 2.293446 10.786901 10.658066
H 4.032958 9.573250 12.524427
H 4.629176 11.723239 8.947514
N 4.316497 9.927246 16.048545
C 5.579144 9.915197 16.496674
C 6.653150 10.541609 15.851109
H 7.628884 10.392749 16.297468
C 6.628425 11.337330 14.687781
N 5.512338 11.553530 14.003187
C 5.900538 9.159575 17.767841
H 5.229985 9.443388 18.583076
H 6.931366 9.328330 18.083000
H 5.758015 8.084565 17.606446
C 7.943359 11.903837 14.210882
H 8.242071 11.385462 13.293413
H 8.726563 11.754848 14.955412
H 7.888606 12.965922 13.963114
N 5.594633 12.426577 12.896766
C 5.535842 13.730016 13.176600
C 5.787068 14.728570 12.218173
H 5.633066 15.746572 12.557851
C 6.371121 14.594508 10.944233
N 6.548923 13.429214 10.311734
C 5.263064 14.172251 14.594325
H 4.535066 13.516924 15.079764
H 4.904390 15.202869 14.614089
H 6.175923 14.118617 15.198283
C 6.775638 15.891441 10.278534
H 7.608175 15.754221 9.586736
H 7.042942 16.653332 11.013451
H 5.922100 16.266896 9.702282
C 3.302646 9.452508 16.929086
C 2.627093 10.391401 17.744157
C 1.604867 9.923086 18.574032
H 1.063539 10.620403 19.204707
C 1.250234 8.576584 18.594830
H 0.449809 8.236797 19.246660
C 1.906743 7.671956 17.768300
H 1.604737 6.628407 17.770740
C 2.937406 8.090892 16.921510
C 2.987772 11.871162 17.687096
H 3.162276 12.105406 16.627338
C 1.866932 12.785171 18.182398
H 0.908677 12.536603 17.725373
H 2.098522 13.824508 17.929973
H 1.759138 12.731521 19.272476
C 4.280604 12.203576 18.449285
H 4.209751 11.866469 19.489774
H 4.438568 13.288018 18.458182
H 5.162485 11.750632 17.994788
C 3.580921 7.122492 15.945391
H 4.525330 7.563962 15.610581
C 3.898821 5.757241 16.560803
H 4.519938 5.855011 17.457271
H 4.435184 5.135761 15.836675
H 2.990169 5.213643 16.841024
C 2.694749 6.956039 14.704988
H 1.702467 6.582641 14.982839
H 3.135720 6.254878 13.992356
H 2.548453 7.898463 14.165634
C 7.466522 13.369827 9.210270
C 8.815912 13.049680 9.499660
C 9.725687 12.942154 8.447291
H 10.761982 12.697665 8.659206
C 9.325122 13.124870 7.128274
H 10.042139 13.020141 6.318707
C 8.001727 13.437398 6.854677
H 7.689999 13.585517 5.824140
C 7.056600 13.579481 7.877428
C 9.272962 12.852716 10.934243
H 8.410529 12.459631 11.482367
C 9.651642 14.183306 11.601786
H 10.456715 14.674979 11.043940

H	10.003719	14.009168	12.625138
H	8.804182	14.869050	11.654889
C	10.429704	11.860285	11.077339
H	10.258113	10.955540	10.490994
H	10.551309	11.573864	12.127519
H	11.379721	12.298808	10.751714
C	5.647957	14.010687	7.509534
H	5.061299	14.040891	8.433974
C	4.970138	13.022537	6.552699
H	5.430038	13.055283	5.558841
H	3.908020	13.266117	6.437602
H	5.057730	12.000245	6.920647
C	5.650403	15.415905	6.887652
H	6.128145	16.152539	7.537678
H	4.629505	15.752965	6.678494
H	6.194774	15.414578	5.937066
N	5.051570	6.457573	11.298595
C	4.165865	5.492139	11.590993
C	2.776238	5.665102	11.565486
H	2.182066	4.833696	11.928705
C	2.037935	6.763792	11.060580
N	2.639668	7.842027	10.587458
C	4.675635	4.122796	11.983675
H	5.416631	3.763090	11.263858
H	3.862855	3.397220	12.043686
H	5.177130	4.162188	12.956587
C	0.533247	6.652732	11.070289
H	0.068353	7.634171	10.961731
H	0.207611	6.197250	12.009675
H	0.174507	6.012094	10.257719
N	1.894600	8.849524	9.954618
C	1.505945	8.632676	8.701644
C	0.828115	9.608628	7.930528
H	0.577629	9.309532	6.919028
C	0.353379	10.864110	8.342043
N	0.666088	11.415909	9.515884
C	1.793198	7.311071	8.026909
H	2.746140	6.894866	8.364408
H	1.816635	7.435076	6.942603
H	1.019501	6.572522	8.261486
C	-0.561111	11.614835	7.400452
H	-1.571663	11.685389	7.817759
H	-0.626265	11.127347	6.425990
H	-0.207027	12.642346	7.265531
C	6.428468	6.122806	11.162719
C	6.952257	5.950400	9.857869
C	8.323155	5.718146	9.721911
H	8.750775	5.589615	8.733587
C	9.163225	5.671433	10.831211
H	10.227953	5.497673	10.699585
C	8.638432	5.860576	12.103240
H	9.302321	5.844126	12.963399
C	7.271176	6.085687	12.292767
C	6.045449	6.049349	8.637709
H	5.401197	6.925781	8.794870
C	6.809590	6.276668	7.334107
H	7.353148	5.374798	7.026960
H	7.516485	7.104135	7.417298
H	6.105441	6.521442	6.533047
C	5.123083	4.829995	8.483174
H	5.712225	3.907155	8.431539
H	4.547468	4.912680	7.554150
H	4.409476	4.742244	9.303809
C	6.738001	6.365777	13.686437
H	5.646608	6.281405	13.656379
C	7.256835	5.373469	14.732940
H	7.060667	4.337324	14.438662
H	6.772525	5.554369	15.697789
H	8.336051	5.475355	14.889298
C	7.064209	7.801952	14.107044
H	8.139687	8.000026	14.038462
H	6.757467	7.984591	15.138890
H	6.549734	8.542946	13.486022
C	-0.061842	12.532808	9.996748
C	-1.318296	12.324830	10.617852
C	-1.936369	13.404501	11.251433
H	-2.889877	13.259497	11.749797
C	-1.343290	14.665507	11.278001
H	-1.837364	15.489291	11.785732
C	-0.116377	14.860013	10.655686
H	0.347882	15.843180	10.677432
C	0.540017	13.805782	10.010909
C	-1.924825	10.930952	10.682610
H	-1.624666	10.387312	9.780432
C	-3.455057	10.930487	10.728017
H	-3.836297	11.313929	11.680960
H	-3.831976	9.908197	10.619712
H	-3.881480	11.539320	9.923940

C	-1.346330	10.159439	11.877873
H	-0.267844	10.017465	11.759458
H	-1.805706	9.167793	11.961619
H	-1.521299	10.701392	12.813763
C	1.908171	14.027842	9.396454
H	2.211967	13.090351	8.914552
C	2.940245	14.349137	10.481447
H	2.718961	15.299112	10.979446
H	3.944832	14.417657	10.058781
H	2.962219	13.574896	11.257600
C	1.888813	15.111898	8.314651
H	1.169132	14.871636	7.525668
H	2.877885	15.209462	7.857484
H	1.617499	16.089060	8.729376
C	1.640042	11.599180	12.564521
H	0.719778	12.198359	12.412354
C	4.222402	10.866839	9.521017
H	3.536499	10.439017	8.780891
N	5.357359	9.939044	9.898487
C	6.337849	9.690021	8.904203
C	7.683680	9.518196	9.320486
C	8.703264	9.487095	8.370025
H	9.732063	9.386175	8.707753
C	8.426085	9.594717	7.008823
H	9.231145	9.611451	6.280474
C	7.098269	9.598528	6.599663
H	6.859291	9.556052	5.538735
C	6.045222	9.583054	7.520571
C	8.011898	9.352966	10.777945
H	7.873960	10.270893	11.363484
H	9.050034	9.041927	10.912048
H	7.398639	8.579441	11.247120
C	4.654668	9.285846	7.020761
H	4.113795	8.676441	7.749936
H	4.713333	8.712800	6.089983
H	4.035282	10.167635	6.824370
N	1.892152	11.432434	13.833942
C	0.913984	11.997579	14.726970
C	0.031342	11.117366	15.377389
C	-0.279836	13.894299	15.601728
C	-1.012461	11.661193	16.129708
C	-1.172270	13.041309	16.242300
H	-0.400076	14.971503	15.688504
H	-1.709250	10.989402	16.624620
H	-1.987488	13.449042	16.833452
C	0.175858	9.633233	15.206889
H	0.154225	9.350529	14.149634
H	1.125925	9.284368	15.617589
H	-0.621989	9.097348	15.725941
C	0.771227	13.391981	14.826239
C	1.660871	14.338080	14.064325
H	1.792549	15.276200	14.611618
H	2.644890	13.911529	13.864219
H	1.217892	14.577736	13.090283

INT-2.log

SCF (B3PW91) = -4011.78223409
E(SCF)+ZPE(0 K)= -4009.897409
H(298 K)= -4009.786135
G(298 K)= -4010.036860
Lowest Frequency = 16.3777cm-1

Mg	3.730893	10.403771	14.325132
Mg	5.922722	11.262213	10.850632
Mg	4.435403	8.280268	10.099201
Mg	2.202389	10.924691	10.670167
H	4.278054	10.327306	10.514067
N	4.465234	9.732504	16.147384
C	5.763802	9.618156	16.450859
C	6.816155	10.058264	15.629014
H	7.814864	9.898495	16.017675
C	6.743382	10.856788	14.469857
N	5.610941	11.060348	13.802371
C	6.162449	9.053621	17.797799
H	5.676573	9.612728	18.603334
H	7.242751	9.096502	17.943486
H	5.839027	8.013669	17.901935
C	8.001968	11.576726	14.043237
H	8.144699	11.559491	12.959793
H	8.879388	11.145123	14.526460
H	7.941464	12.633073	14.327508
N	5.685227	12.000932	12.742353
C	5.440607	13.266735	13.069674
C	5.464256	14.318564	12.131318
H	5.181919	15.290389	12.518089
C	5.855717	14.289901	10.785665
N	6.185906	13.175937	10.115978

C	5.099820	13.614842	14.496400
H	4.165884	13.118269	14.784991
H	4.966635	14.690291	14.617901
H	5.864132	13.272277	15.198378
C	5.931878	15.634666	10.092967
H	6.941692	16.049169	10.168708
H	5.246929	16.345032	10.559122
H	5.696951	15.555648	9.028832
C	3.510833	9.499936	17.188067
C	2.944037	10.619738	17.846254
C	1.964243	10.390357	18.815395
H	1.507648	11.232691	19.323239
C	1.538586	9.102915	19.124930
H	0.768907	8.950130	19.876721
C	2.085229	8.016143	18.454502
H	1.728986	7.015827	18.682372
C	3.072758	8.190746	17.480717
C	3.355456	12.039425	17.478774
H	3.388733	12.078671	16.383124
C	2.350559	13.108222	17.912478
H	1.335427	12.866834	17.590188
H	2.622560	14.066138	17.458221
H	2.355150	13.249847	19.000208
C	4.753621	12.403600	17.999157
H	4.798939	12.290938	19.088509
H	4.981293	13.448615	17.760348
H	5.536154	11.787224	17.553015
C	3.589594	6.991148	16.704360
H	4.551879	7.269419	16.263225
C	3.824124	5.762312	17.589434
H	4.456553	6.003521	18.449979
H	4.312480	4.965219	17.019567
H	2.884133	5.349763	17.970422
C	2.640203	6.658418	15.545209
H	1.640622	6.415503	15.921416
H	3.006399	5.803087	14.966526
H	2.533891	7.504520	14.861449
C	6.782506	13.324286	8.830768
C	8.126025	13.760706	8.723016
C	8.703118	13.825676	7.452333
H	9.738429	14.140680	7.355390
C	7.979419	13.502324	6.308320
H	8.446174	13.570109	5.329504
C	6.659790	13.083148	6.426449
H	6.100870	12.815657	5.533671
C	6.045183	12.971882	7.678366
C	8.949531	14.150312	9.944233
H	8.266441	14.276944	10.788479
C	9.691106	15.479261	9.743768
H	10.507846	15.380426	9.020457
H	10.134146	15.810139	10.688930
H	9.025073	16.269184	9.382095
C	9.934763	13.046242	10.340630
H	9.417897	12.115192	10.583386
H	10.518719	13.351354	11.216293
H	10.631508	12.826004	9.526218
C	4.635903	12.409747	7.770249
H	4.297665	12.514245	8.809990
C	4.640731	10.913625	7.421863
H	4.943385	10.757405	6.380519
H	3.642729	10.492246	7.560703
H	5.341307	10.356422	8.053137
C	3.631671	13.160840	6.890774
H	3.595143	14.223819	7.147538
H	2.632305	12.732567	7.020768
H	3.888653	13.081258	5.828919
N	4.400452	6.240557	10.482536
C	3.328249	5.455052	10.610207
C	2.013121	5.923337	10.477112
H	1.224360	5.211577	10.690000
C	1.589993	7.204840	10.073581
N	2.443457	8.170800	9.733030
C	3.525957	3.984316	10.892757
H	4.057564	3.513204	10.058282
H	2.574776	3.468650	11.033455
H	4.146771	3.831215	11.781482
C	0.108179	7.479214	10.040729
H	-0.100571	8.407867	10.576329
H	-0.447332	6.664077	10.506530
H	-0.265754	7.615423	9.021399
N	1.876729	9.402333	9.344649
C	1.481560	9.512974	8.079812
C	0.789662	10.642985	7.607950
H	0.500492	10.618725	6.563923
C	0.294591	11.718424	8.370783
N	0.669905	11.972430	9.627480
C	1.771583	8.397706	7.104254
H	2.812214	8.071246	7.203230

H	1.592057	8.724452	6.078679
H	1.154316	7.514990	7.297402
C	-0.758080	12.581137	7.705349
H	-1.667268	12.601319	8.314187
H	-1.010657	12.201129	6.714266
H	-0.428407	13.619723	7.606752
C	5.704208	5.680538	10.577938
C	6.370850	5.280416	9.401047
C	7.724807	4.941458	9.483072
H	8.260836	4.656875	8.581365
C	8.399705	4.972946	10.699417
H	9.454769	4.718012	10.744861
C	7.713503	5.312805	11.863288
H	8.240733	5.300975	12.812867
C	6.361984	5.666553	11.827261
C	5.646929	5.252759	8.064247
H	4.577831	5.371973	8.268463
C	6.069860	6.423159	7.164484
H	7.150010	6.408164	6.980999
H	5.826918	7.389950	7.618526
H	5.559088	6.371998	6.196517
C	5.835087	3.914774	7.339042
H	6.873904	3.765304	7.025214
H	5.212153	3.877550	6.438994
H	5.557594	3.073499	7.982130
C	5.597260	6.031976	13.089392
H	4.539878	5.824149	12.896279
C	6.017447	5.194101	14.299240
H	5.964372	4.122534	14.082281
H	5.362662	5.404890	15.147714
H	7.039024	5.424927	14.619896
C	5.697607	7.525576	13.419630
H	6.679364	7.783828	13.821013
H	4.958799	7.803793	14.177727
H	5.536510	8.157597	12.539338
C	-0.076586	12.952730	10.351569
C	-1.054714	12.512034	11.272914
C	-1.746761	13.473029	12.015055
H	-2.479838	13.158434	12.749603
C	-1.488418	14.830481	11.857034
H	-2.033908	15.559085	12.450226
C	-0.508547	15.249399	10.963729
H	-0.293928	16.309310	10.865241
C	0.218480	14.324192	10.210087

C	-1.314500	11.027108	11.476063
H	-0.337184	10.526817	11.445122
C	-2.165300	10.433475	10.343447
H	-3.113615	10.975562	10.254483
H	-2.396558	9.383004	10.551958
H	-1.649517	10.473007	9.381613
C	-1.933527	10.692486	12.832912
H	-1.383015	11.157534	13.654228
H	-1.917130	9.608314	12.984402
H	-2.980225	11.013176	12.891884
C	1.365287	14.762525	9.321524
H	1.456200	14.036869	8.509687
C	2.676542	14.699978	10.105535
H	2.679269	15.412523	10.936665
H	3.522976	14.922908	9.453523
H	2.842111	13.710150	10.538539
C	1.178182	16.139044	8.682065
H	0.217639	16.212470	8.161515
H	1.976007	16.325722	7.955266
H	1.222647	16.944938	9.422890
C	6.569884	8.604520	9.864362
H	7.251536	7.792415	9.551207
N	7.202476	9.702275	10.163328
C	8.635276	9.701486	10.035790
C	9.420964	9.372379	11.150889
C	10.812204	9.442199	11.026660
H	11.430436	9.203011	11.888582
C	11.404590	9.785492	9.815446
H	12.486577	9.839596	9.733880
C	10.606780	10.036210	8.701790
H	11.066017	10.275132	7.745836
C	9.213485	9.995585	8.789511
C	8.780466	8.850785	12.402499
H	7.819152	9.325402	12.600933
H	9.423338	8.981977	13.276201
H	8.571189	7.781147	12.287453
C	8.349212	10.227744	7.586391
H	7.784556	9.325089	7.324526
H	8.945169	10.523940	6.721077
H	7.618630	11.016546	7.773211
N	2.107153	11.534589	14.031585
C	1.044973	12.055649	14.784873
C	0.260294	11.202244	15.610244
C	-0.366566	13.915124	15.508627

C	-0.796081	11.725326	16.358892
C	-1.133693	13.074609	16.306189
H	-0.595197	14.978631	15.470166
H	-1.379240	11.045215	16.976305
H	-1.967107	13.463181	16.885004
C	0.465517	9.714084	15.633138
H	1.509861	9.433189	15.752409
H	-0.072635	9.262140	16.470025
H	0.108722	9.258400	14.705985
C	0.721579	13.444328	14.768140
C	1.532341	14.465314	14.009505
H	1.443140	15.446084	14.488940
H	2.587801	14.199475	13.972274
H	1.192134	14.573957	12.974668
C	2.277918	9.390211	12.915865
O	1.306033	8.804302	12.652347
C	2.176394	11.929163	12.646868
H	3.026451	12.603863	12.441018
H	1.259259	12.452139	12.348274

INT-3.log

SCF (B3PW91) = -4011.81989504
E(SCF)+ZPE(0 K)= -4009.934615
H(298 K)= -4009.823501
G(298 K)= -4010.075291
Lowest Frequency = 19.9031cm-1

Mg	3.871359	10.276252	14.585913
Mg	5.593384	11.522621	10.834859
Mg	4.000680	8.036427	10.416904
Mg	2.182487	10.685636	10.039888
H	3.882824	11.294483	10.161577
N	4.811561	9.752676	16.324248
C	6.145538	9.692731	16.448903
C	7.050568	10.208359	15.510288
H	8.100073	10.059183	15.736106
C	6.792514	11.040129	14.391383
N	5.578547	11.217693	13.887164
C	6.728221	9.061315	17.694174
H	6.406109	9.598106	18.591319
H	7.819091	9.046663	17.668760
H	6.362294	8.034133	17.802163
C	7.962780	11.777032	13.785596
H	7.988570	11.661489	12.698630
H	8.908641	11.425916	14.199805
H	7.875280	12.851940	13.977399
N	5.487076	12.133705	12.805470
C	5.206698	13.394219	13.130662
C	5.205584	14.457011	12.200208
H	4.862064	15.406950	12.591843
C	5.700884	14.490386	10.886523
N	6.100078	13.413619	10.202865
C	4.884278	13.737241	14.563717
H	4.030473	13.149718	14.917043
H	4.634683	14.793672	14.667202
H	5.725397	13.509508	15.225424
C	5.828994	15.855191	10.249189
H	6.862325	16.209250	10.323784
H	5.184955	16.583340	10.745267
H	5.581919	15.822265	9.184599
C	4.020525	9.646756	17.508470
C	3.777103	10.823466	18.254270
C	2.980484	10.740743	19.399717
H	2.793448	11.638818	19.982539
C	2.417275	9.534035	19.798776
H	1.796905	9.487746	20.689710
C	2.636988	8.388557	19.040176
H	2.174316	7.453751	19.342649
C	3.432180	8.423026	17.891251
C	4.362225	12.159905	17.830134
H	4.791477	12.017803	16.834250
C	3.295308	13.255522	17.725607
H	2.483616	12.962461	17.054369
H	3.741915	14.178704	17.340380
H	2.855689	13.488676	18.701560
C	5.503721	12.591856	18.759988
H	5.145440	12.717136	19.788370
H	5.925744	13.548421	18.431453
H	6.311916	11.854658	18.771866
C	3.594590	7.189776	17.024672
H	4.520571	7.318054	16.454457
C	3.721482	5.891257	17.825392
H	4.509747	5.966966	18.581383
H	3.964911	5.054606	17.162986
H	2.788014	5.632915	18.336804
C	2.431909	7.112120	16.022395
H	1.484281	6.968868	16.553419

H	2.561105	6.279758	15.321751
H	2.336380	8.036659	15.442193
C	6.817654	13.595024	8.985594
C	8.205591	13.863962	9.034624
C	8.921340	13.903131	7.835383
H	9.992564	14.085635	7.863144
C	8.290026	13.717089	6.609347
H	8.864361	13.753750	5.687577
C	6.919506	13.489042	6.571418
H	6.427744	13.343275	5.613112
C	6.163195	13.415893	7.745846
C	8.927672	14.109433	10.351195
H	8.173741	14.162024	11.142513
C	9.691924	15.440827	10.341791
H	10.535538	15.413256	9.643279
H	10.097301	15.653106	11.336922
H	9.050771	16.278235	10.049064
C	9.871223	12.954684	10.701780
H	9.335746	12.005466	10.760616
H	10.358080	13.134907	11.666798
H	10.652878	12.837469	9.944176
C	4.674094	13.122896	7.662585
H	4.263761	13.216072	8.672362
C	4.399321	11.686589	7.198030
H	4.823583	11.501631	6.204311
H	3.319897	11.499944	7.148313
H	4.827620	10.954750	7.889593
C	3.951928	14.129549	6.759509
H	4.111869	15.157472	7.100733
H	2.874991	13.934939	6.752808
H	4.301179	14.065867	5.723375
N	4.176792	5.994588	10.376895
C	3.151618	5.168732	10.156345
C	1.891419	5.615443	9.726880
H	1.097396	4.877332	9.722141
C	1.526696	6.903676	9.266037
N	2.410410	7.890860	9.115394
C	3.342517	3.690327	10.394962
H	4.076502	3.283237	9.690513
H	2.409629	3.136249	10.278626
H	3.743014	3.513843	11.398864
C	0.063976	7.147738	8.983629
H	-0.231230	8.121155	9.386278
H	-0.549744	6.367414	9.436396
H	-0.156706	7.170456	7.912548
N	1.922744	9.149844	8.725030
C	1.534497	9.363614	7.475185
C	0.811333	10.527469	7.119154
H	0.547397	10.602543	6.069861
C	0.249324	11.501968	7.964239
N	0.571191	11.657166	9.258433
C	1.839607	8.364340	6.386922
H	2.661101	7.709125	6.684404
H	2.108646	8.895717	5.469968
H	0.972818	7.736952	6.154360
C	-0.792269	12.410230	7.349706
H	-1.674212	12.472180	7.994127
H	-1.099268	12.052735	6.365809
H	-0.410840	13.430159	7.243308
C	5.481619	5.493457	10.629443
C	6.313136	5.160370	9.534218
C	7.655217	4.864179	9.782984
H	8.314426	4.625760	8.953589
C	8.172417	4.886497	11.076746
H	9.221912	4.663629	11.247552
C	7.339416	5.194759	12.145957
H	7.742956	5.207623	13.155517
C	5.989889	5.502206	11.943376
C	5.776566	5.213066	8.111642
H	4.695986	5.043126	8.153537
C	5.983137	6.610786	7.508279
H	7.044523	6.882492	7.512016
H	5.436430	7.371656	8.074044
H	5.621214	6.646077	6.474581
C	6.374277	4.136881	7.200299
H	7.432840	4.321190	6.986945
H	5.849843	4.124906	6.239210
H	6.289789	3.141804	7.648938
C	5.106604	5.873105	13.120162
H	4.085941	5.989303	12.735712
C	5.082950	4.768842	14.182091
H	4.782033	3.808647	13.751322
H	4.379320	5.021738	14.979770
H	6.067600	4.635357	14.643270
C	5.526302	7.214826	13.730755
H	6.521847	7.160484	14.182189
H	4.825312	7.520585	14.510879
H	5.553100	8.013571	12.982164

C	-0.158680	12.581510	10.066063
C	-1.094900	12.072194	10.999082
C	-1.759416	12.973166	11.835274
H	-2.468027	12.604916	12.568765
C	-1.516435	14.341293	11.761848
H	-2.038099	15.020936	12.429158
C	-0.578449	14.825990	10.860151
H	-0.359853	15.889911	10.834983
C	0.124276	13.962410	10.013533
C	-1.377986	10.579510	11.082162
H	-0.420602	10.055964	10.950995
C	-2.292796	10.120050	9.936790
H	-3.237477	10.674680	9.957572
H	-2.524309	9.053532	10.034957
H	-1.824563	10.271799	8.961372
C	-1.955865	10.142284	12.430656
H	-1.379545	10.541749	13.270769
H	-1.950688	9.049631	12.499799
H	-2.995532	10.468437	12.547564
C	1.248900	14.507299	9.154372
H	1.527222	13.732706	8.433918
C	2.481113	14.770739	10.026801
H	2.252436	15.483830	10.825394
H	3.294719	15.184666	9.426249
H	2.847809	13.855571	10.501712
C	0.860511	15.765552	8.371192
H	-0.060281	15.619666	7.797273
H	1.659845	16.037064	7.674264
H	0.703591	16.622099	9.035508
C	6.072421	8.667898	10.332595
H	6.744707	7.800841	10.170805
N	6.732603	9.788687	10.371990
C	8.160170	9.718965	10.188165
C	8.990624	9.299391	11.240896
C	10.374700	9.304075	11.031611
H	11.027949	9.000006	11.845923
C	10.916039	9.668743	9.803772
H	11.993110	9.670420	9.661523
C	10.070876	10.011060	8.751465
H	10.484127	10.265559	7.778878
C	8.685799	10.036890	8.923496
C	8.423222	8.771555	12.525511
H	7.484710	9.252283	12.799325
H	9.124115	8.895424	13.354820
H	8.205871	7.702447	12.422592
C	7.765554	10.341522	7.781655
H	7.117591	9.486211	7.558890
H	8.321658	10.604205	6.879886
H	7.113646	11.183230	8.017884
N	1.978828	10.911607	14.483630
C	1.107179	11.872630	15.017034
C	0.330327	11.557767	16.157408
C	0.178078	14.120182	15.046752
C	-0.484775	12.536081	16.731040
C	-0.561142	13.817254	16.186193
H	0.116054	15.112595	14.604189
H	-1.070800	12.286170	17.613494
H	-1.196888	14.570743	16.644478
C	0.401038	10.172125	16.731332
H	1.404229	9.950166	17.101117
H	-0.303112	10.045529	17.559139
H	0.181726	9.419769	15.965945
C	1.007227	13.165476	14.449886
C	1.771697	13.508231	13.205509
H	2.130486	14.541441	13.214299
H	2.633182	12.846480	13.081601
H	1.135988	13.398781	12.321918
C	2.765289	9.568122	12.757217
O	2.764158	9.297365	11.508496
C	1.549219	10.309109	13.246076
H	1.193682	11.036485	12.470343
H	0.712091	9.579759	13.337109

INT-4.log

SCF (B3PW91) = -4011.81844650
E(SCF)+ZPE(0 K)= -4009.932215
H(298 K)= -4009.821540
G(298 K)= -4010.071031
Lowest Frequency = 21.2400cm-1

Mg	12.741416	7.177414	8.638469
Mg	12.451390	9.581580	6.011364
N	13.946863	5.542087	8.026216
C	15.229802	5.557653	7.645483
C	15.986586	6.722132	7.416972
H	17.017746	6.577802	7.116926
C	15.522714	8.048935	7.429799

N	14.300396	8.335184	7.843317
C	15.958939	4.248463	7.417612
H	15.353661	3.551761	6.832344
H	16.165344	3.757308	8.373837
H	16.908641	4.409183	6.904454
C	16.420682	9.151186	6.932720
H	15.995665	9.602425	6.031466
H	17.418217	8.778069	6.695070
H	16.506184	9.952590	7.672875
N	13.782692	9.621701	7.606757
C	13.706396	10.448868	8.621098
C	12.831079	11.591348	8.594458
H	13.004113	12.282726	9.411721
C	12.430753	12.254626	7.381237
N	12.388068	11.645845	6.212051
C	14.554898	10.254098	9.847758
H	14.819482	9.205012	9.981495
H	14.046417	10.605378	10.743680
H	15.473381	10.841541	9.730090
C	12.091341	13.721711	7.479782
H	12.164205	14.215411	6.510285
H	12.732524	14.241168	8.193414
H	11.055017	13.804221	7.822242
C	13.295477	4.273883	8.067712
C	13.436971	3.433708	9.191593
C	12.783469	2.198734	9.191199
H	12.886615	1.539750	10.049347
C	11.974561	1.817554	8.127968
H	11.451492	0.865498	8.151364
C	11.806451	2.674094	7.043992
H	11.138588	2.378321	6.242465
C	14.227178	3.880974	10.410056
H	14.831648	4.744989	10.115354
C	13.264155	4.353214	11.507269
H	12.565264	5.096916	11.114653
H	13.808390	4.794624	12.350108
H	12.668810	3.515035	11.884521
C	15.176004	2.805249	10.949503
H	14.626259	1.953474	11.364762
H	15.796708	3.214075	11.754084
H	15.839326	2.420379	10.168343
C	12.356449	12.405284	5.001705
C	13.597655	12.680490	4.381930
C	13.595304	13.384339	3.175751

H	14.537902	13.616094	2.689162
C	12.403074	13.790516	2.582999
H	12.419656	14.333315	1.642014
C	11.192047	13.488322	3.193508
H	10.263203	13.790339	2.717103
C	11.144393	12.793379	4.406528
C	14.902865	12.254268	5.034500
H	14.676937	11.402223	5.687036
C	15.449474	13.362633	5.945776
H	15.664458	14.267152	5.365681
H	16.376491	13.039498	6.432508
H	14.732037	13.627057	6.727302
C	15.966904	11.808645	4.027354
H	15.573859	11.062634	3.329684
H	16.821942	11.371030	4.553249
H	16.349786	12.649132	3.438710
C	9.806513	12.425775	5.018112
H	9.995016	11.988783	6.005875
C	9.143558	11.353398	4.145160
H	8.199479	11.006930	4.577760
H	9.822959	10.501221	4.041204
H	8.930768	11.741063	3.142805
C	8.903465	13.646940	5.218350
H	8.666185	14.134741	4.267182
H	9.378908	14.389265	5.867516
H	7.951383	13.354951	5.674790
C	12.438410	8.030659	10.677689
H	12.735924	7.231229	11.394763
N	11.778352	8.974242	11.302387
C	11.494981	8.786494	12.700483
C	10.598939	7.768544	13.090236
C	10.404775	7.545537	14.456608
H	9.720485	6.759907	14.767126
C	11.053092	8.320507	15.415070
H	10.884568	8.135064	16.472106
C	11.893213	9.352874	15.010572
H	12.384134	9.979460	15.750868
C	12.126043	9.601681	13.654260
C	9.838140	6.959147	12.075288
H	9.310294	7.616190	11.377461
H	9.101165	6.316393	12.562951
H	10.484580	6.322726	11.463636
C	13.077153	10.686579	13.240253
H	12.675822	11.276252	12.411759

H	14.038558	10.269285	12.919652
H	13.259365	11.384966	14.059644
Mg	9.515247	6.962382	8.469765
Mg	11.075851	10.491836	10.070937
N	7.799631	5.859777	8.638960
C	6.739028	6.247336	9.344102
C	6.600452	7.525005	9.908549
H	5.682640	7.679774	10.466336
C	7.431864	8.664005	9.846440
N	8.632531	8.695276	9.260167
C	5.598826	5.282317	9.590727
H	5.801288	4.691906	10.490628
H	5.480959	4.579519	8.763321
H	4.658154	5.814957	9.744760
C	6.847231	9.865891	10.558442
H	5.845466	10.083898	10.176631
H	7.450940	10.764297	10.477081
H	6.740092	9.619432	11.619128
N	9.292923	9.946833	9.227617
C	8.732175	10.908452	8.490849
C	8.997991	12.284832	8.699216
H	8.516523	12.951503	7.992503
C	9.533891	12.919010	9.846930
N	10.310909	12.301222	10.737461
C	7.699488	10.551018	7.450732
H	7.948283	9.597129	6.976505
H	7.639939	11.336134	6.696938
H	6.703332	10.442966	7.891260
C	9.185567	14.381774	10.018135
H	9.239748	14.681466	11.066746
H	8.187185	14.600812	9.634550
H	9.899559	15.001458	9.463523
C	7.850137	4.552993	8.076633
C	7.617306	4.401461	6.688615
C	7.760445	3.128520	6.129317
H	7.593267	2.985379	5.066857
C	8.134152	2.035905	6.907822
H	8.253138	1.057803	6.449359
C	8.363177	2.202052	8.268069
H	8.670863	1.350439	8.868694
C	8.220810	3.452060	8.876682
C	7.212105	5.602376	5.843854
H	7.849301	6.441892	6.157975
C	7.416243	5.399665	4.341524
H	8.434522	5.084785	4.097577
H	7.214254	6.337648	3.815535
H	6.724269	4.652021	3.937249
C	5.754003	6.017172	6.098739
H	5.076780	5.179654	5.896448
H	5.474341	6.844876	5.436949
H	5.595286	6.348667	7.125568
C	8.496139	3.610723	10.362025
H	8.182036	4.616929	10.655250
C	7.708614	2.605498	11.211626
H	6.637924	2.630946	10.985223
H	8.056942	1.580478	11.043482
H	7.840068	2.823185	12.277232
C	9.995371	3.508706	10.650416
H	10.564601	4.220094	10.045250
H	10.198341	3.712546	11.707564
H	10.372794	2.509653	10.415271
C	10.618408	12.871603	12.013977
C	9.907722	12.389404	13.140396
C	10.292094	12.806689	14.415554
H	9.760382	12.426357	15.281663
C	11.342548	13.700506	14.597953
H	11.629436	14.012608	15.598422
C	12.012455	14.196806	13.488529
H	12.825400	14.905353	13.625331
C	11.672303	13.795137	12.192209
C	8.691354	11.505153	12.954945
H	8.875735	10.888511	12.068417
C	7.467710	12.394963	12.679550
H	7.330738	13.105843	13.502216
H	6.554900	11.796740	12.595997
H	7.581722	12.967585	11.755219
C	8.410862	10.567345	14.127195
H	9.303538	10.011573	14.416732
H	7.637266	9.844126	13.848772
H	8.039780	11.109913	15.004453
C	12.460726	14.354767	11.027327
H	12.036207	13.914078	10.123474
C	13.943279	13.962690	11.104479
H	14.473168	14.258461	10.191663
H	14.072367	12.885297	11.239462
H	14.435619	14.456732	11.948696
C	12.326231	15.879043	10.914316
H	12.799864	16.380318	11.765256

H	11.279315	16.191694	10.888193
H	12.815996	16.241635	10.003470
O	11.481782	8.200839	7.177102
C	10.407853	7.727814	6.649139
H	9.312593	8.599204	5.135925
C	10.261219	8.018796	5.183873
H	9.981947	7.050814	4.746444
N	11.407359	8.637717	4.567955
C	11.855322	8.141771	3.353766
C	13.263229	8.012532	3.162492
C	13.784780	7.488509	1.981689
H	14.864049	7.387621	1.883323
C	12.949174	7.063755	0.952071
H	13.359810	6.641864	0.039429
C	11.575418	7.214379	1.112514
H	10.908476	6.926809	0.301234
C	11.011328	7.761050	2.268960
C	14.197045	8.384245	4.276750
H	15.236296	8.152036	4.031347
H	13.970612	7.827279	5.199211
H	14.199029	9.469981	4.481376
C	9.521995	8.001145	2.286419
H	9.279226	8.944221	2.781038
H	8.964093	7.212893	2.801554
H	9.134409	8.052089	1.264517
C	12.465749	3.905147	6.983147
C	12.321762	4.818667	5.772655
H	12.301231	5.852739	6.141794
C	11.030276	4.584500	4.991019
H	10.149770	4.672694	5.634578
H	10.958600	5.314556	4.178415
H	11.008333	3.592741	4.526046
C	13.522425	4.719172	4.818907
H	14.447527	5.051621	5.293734
H	13.658774	3.685605	4.480157
H	13.356266	5.351894	3.940362
H	11.096500	6.259942	9.081962

INT-5.log

SCF (B3PW91) = -4011.88427664
E(SCF)+ZPE(0 K)= -4009.997200
H(298 K)= -4009.886329
G(298 K)= -4010.136330
Lowest Frequency = 21.0420cm-1

Mg	12.467939	6.915964	8.550323
Mg	11.682831	9.286370	6.129027
N	13.920395	5.391164	8.408985
C	15.188152	5.545705	7.997458
C	15.728517	6.723315	7.450113
H	16.751936	6.660406	7.100135
C	15.086658	7.958833	7.238777
N	13.862810	8.164605	7.687127
C	16.155724	4.385058	8.125928
H	15.783500	3.484362	7.633114
H	16.281686	4.121305	9.180654
H	17.133617	4.634644	7.711274
C	15.786545	9.015307	6.426376
H	15.319186	9.051204	5.435459
H	16.845273	8.785010	6.296885
H	15.686926	10.012557	6.860920
N	13.230223	9.402195	7.489249
C	13.367529	10.306897	8.425768
C	12.527834	11.480624	8.465086
H	12.876047	12.229712	9.169800
C	11.914913	12.063028	7.302786
N	11.619563	11.369565	6.217416
C	14.404913	10.152262	9.501588
H	14.476537	9.109857	9.817053
H	14.173186	10.776762	10.362118
H	15.383172	10.456707	9.112973
C	11.641008	13.547571	7.348957
H	11.634567	13.983836	6.349323
H	12.369019	14.077859	7.964291
H	10.646311	13.693610	7.784789
C	13.541077	4.063448	8.780253
C	13.588119	3.659942	10.131388
C	13.299841	2.329449	10.450527
H	13.347472	2.009668	11.488693
C	12.961354	1.409727	9.465127
H	12.756204	0.375224	9.727334
C	12.855044	1.833450	8.144751
H	12.552582	1.123732	7.378959
C	13.904705	4.642813	11.244100
H	14.048372	5.623611	10.776617
C	12.717558	4.738230	12.212230
H	11.789688	4.907447	11.659124

H	12.848735	5.557369	12.927906
H	12.598208	3.810817	12.783702
C	15.193877	4.298102	11.998625
H	15.122225	3.313640	12.474579
H	15.386219	5.035910	12.785624
H	16.060716	4.283326	11.331428
C	11.443720	12.058683	4.974003
C	12.589816	12.211243	4.157199
C	12.436114	12.830694	2.915095
H	13.300844	12.956598	2.271645
C	11.192504	13.278421	2.480876
H	11.093674	13.752813	1.508456
C	10.075432	13.101686	3.287348
H	9.103003	13.428542	2.930859
C	10.177459	12.487572	4.540087
C	13.958701	11.743853	4.627134
H	13.800724	10.932020	5.345258
C	14.685119	12.865401	5.384534
H	14.850890	13.728277	4.729404
H	15.660851	12.516517	5.741140
H	14.112731	13.206400	6.251435
C	14.835572	11.195048	3.500095
H	14.327166	10.401308	2.947581
H	15.760407	10.784339	3.917758
H	15.129740	11.976763	2.791040
C	8.929563	12.206719	5.353517
H	9.239227	11.967544	6.376859
C	8.229315	10.971710	4.765265
H	7.271668	10.774913	5.253897
H	8.857110	10.081555	4.877522
H	8.042525	11.106094	3.694614
C	7.991992	13.414989	5.428837
H	7.574408	13.673362	4.450375
H	8.516586	14.294518	5.815417
H	7.144694	13.207524	6.090303
C	12.183647	8.005013	10.526843
H	12.467888	7.166300	11.201821
N	11.727856	9.006230	11.241297
C	11.629105	8.822298	12.664031
C	10.736435	7.857045	13.173996
C	10.735533	7.614574	14.550763
H	10.056198	6.866366	14.951431
C	11.569905	8.326494	15.408116
H	11.554034	8.127183	16.475846
C	12.396723	9.321266	14.894083
H	13.028061	9.903483	15.560520
C	12.436459	9.588034	13.522029
C	9.780780	7.122981	12.274073
H	9.346173	7.802124	11.537875
H	8.970131	6.671186	12.851203
H	10.266259	6.329974	11.696609
C	13.368586	10.635731	12.984625
H	12.874105	11.279478	12.251086
H	14.236399	10.179188	12.496659
H	13.724381	11.290841	13.782708
Mg	9.588318	7.200037	8.581058
Mg	11.001553	10.602665	10.137567
N	7.834867	6.197359	8.246738
C	6.761788	6.405683	8.999661
C	6.657868	7.464361	9.930271
H	5.780439	7.434933	10.565972
C	7.472348	8.596313	10.087440
N	8.601152	8.796306	9.387333
C	5.567357	5.485154	8.895134
H	5.416021	5.152995	7.865286
H	4.659366	5.975013	9.251402
H	5.729983	4.588426	9.501265
C	7.050039	9.574815	11.159708
H	6.036943	9.363912	11.504602
H	7.105435	10.621226	10.859054
H	7.730381	9.458489	12.010089
N	9.155239	10.093799	9.432263
C	8.422834	11.062547	8.881062
C	8.667302	12.438241	9.107454
H	8.046257	13.111810	8.526752
C	9.415194	13.050156	10.137159
N	10.325203	12.412844	10.877424
C	7.228221	10.703279	8.032290
H	7.439067	9.817119	7.427576
H	6.956138	11.540427	7.390669
H	6.352526	10.463336	8.643875
C	9.141662	14.520222	10.367080
H	9.342916	14.806690	11.401367
H	8.110788	14.779807	10.118918
H	9.801419	15.116750	9.726314
C	7.847285	5.134758	7.297914
C	7.718084	5.463882	5.927824
C	7.766378	4.427897	4.992589

H	7.668170	4.652156	3.935775	H	8.599856	11.274238	15.391628
C	7.955278	3.106292	5.389589	C	12.548729	14.395900	10.859343
H	7.994815	2.315858	4.645123	H	11.996125	13.958976	10.023884
C	8.123626	2.806139	6.735052	C	14.017760	13.966369	10.742541
H	8.312156	1.778976	7.035369	H	14.428019	14.237401	9.763083
C	8.084303	3.807083	7.711972	H	14.135598	12.887635	10.876091
C	7.536329	6.913844	5.499989	H	14.631599	14.454957	11.506579
H	8.209352	7.517150	6.126005	C	12.439482	15.922729	10.751817
C	7.927097	7.172985	4.045032	H	13.036403	16.414810	11.527291
H	8.947185	6.834901	3.844459	H	11.407282	16.263509	10.865088
H	7.877258	8.245100	3.835261	H	12.811247	16.267381	9.780347
H	7.244024	6.678697	3.344968	O	10.890819	7.701691	7.148473
C	6.106172	7.411370	5.757353	C	10.964494	6.617645	6.243177
H	5.383549	6.805957	5.198682	H	10.574687	5.664610	6.584849
H	6.001422	8.451672	5.428517	C	11.428174	6.819813	4.987992
H	5.844239	7.369295	6.816501	H	11.487719	5.963077	4.316586
C	8.351834	3.446812	9.164738	N	11.788050	8.066129	4.529314
H	8.159026	4.335702	9.774568	C	12.515239	8.150993	3.328704
C	7.439070	2.318163	9.663764	C	13.758108	7.491800	3.159971
H	6.381826	2.526625	9.471678	C	14.470864	7.667852	1.969080
H	7.679592	1.367648	9.175071	H	15.432144	7.169799	1.856393
H	7.570382	2.172044	10.741262	C	13.984030	8.473398	0.944168
C	9.824807	3.067422	9.362390	H	14.555947	8.606226	0.029689
H	10.490855	3.883127	9.069426	C	12.753227	9.106940	1.105482
H	10.029518	2.831428	10.412447	H	12.353066	9.727830	0.306655
H	10.089202	2.187513	8.768109	C	12.009296	8.951965	2.276786
C	10.821692	12.968817	12.097416	C	14.329562	6.620256	4.245148
C	10.254860	12.499272	13.307973	H	14.027367	5.574598	4.123608
C	10.807778	12.917379	14.519164	H	13.982282	6.924519	5.233317
H	10.388901	12.551631	15.450948	H	15.423365	6.643570	4.242358
C	11.891050	13.790858	14.555175	C	10.667014	9.605141	2.421666
H	12.310131	14.103344	15.507757	H	10.682867	10.377086	3.193715
C	12.427209	14.263154	13.365092	H	9.910658	8.875066	2.723915
H	13.267960	14.951785	13.391226	H	10.352588	10.081550	1.488802
C	11.911571	13.864757	12.126818	C	13.115325	3.157511	7.781553
C	9.040054	11.592408	13.275869	C	12.920078	3.591144	6.337634
H	9.157914	10.945833	12.398892	H	12.969347	4.684739	6.313613
C	7.759705	12.417735	13.074037	C	11.534366	3.181967	5.825009
H	7.650963	13.150067	13.882038	H	10.742808	3.489859	6.511598
H	6.877814	11.768304	13.085308	H	11.332828	3.638709	4.850466
H	7.765849	12.957080	12.124264	H	11.448043	2.096891	5.697437
C	8.896730	10.699404	14.506582	C	14.014127	3.044375	5.411123
H	9.825041	10.172444	14.730776	H	14.991983	3.476530	5.639698
H	8.115494	9.951231	14.335993	H	14.092543	1.954361	5.498192

H	13.784773	3.277555	4.365273
H	10.878203	6.046046	9.217296

INT-6.log

SCF (B3PW91) = -4011.90592832
E(SCF)+ZPE(0 K)= -4010.014868
H(298 K)= -4009.904347
G(298 K)= -4010.154473
Lowest Frequency = 22.0084cm-1

Mg	12.302864	7.107003	8.224213
Mg	11.498041	9.461317	5.771915
N	13.917416	5.840329	8.521320
C	15.178826	6.141000	8.194842
C	15.608709	7.388067	7.702354
H	16.658172	7.447791	7.438666
C	14.846179	8.529509	7.388193
N	13.551581	8.616696	7.666859
C	16.250866	5.073223	8.277222
H	16.210337	4.446173	7.381091
H	16.109030	4.409879	9.132070
H	17.246137	5.518417	8.332162
C	15.516156	9.645771	6.627679
H	15.088753	9.683899	5.618588
H	16.590982	9.479078	6.543503
H	15.340860	10.626474	7.076935
N	12.864980	9.776587	7.251975
C	12.808431	10.780140	8.111030
C	12.086821	11.970461	7.853553
H	12.085604	12.705130	8.649166
C	11.463903	12.342199	6.652175
N	11.281638	11.499067	5.627130
C	13.528127	10.652972	9.424482
H	13.360370	9.659536	9.855984
H	13.214154	11.433755	10.116976
H	14.611888	10.742524	9.300926
C	10.990790	13.771015	6.521024
H	11.423988	14.246013	5.635467
H	11.245435	14.359282	7.403566
H	9.903649	13.797257	6.390747
C	13.648511	4.515556	8.984251
C	13.652091	4.258876	10.376041
C	13.446606	2.945264	10.808077
H	13.460934	2.724462	11.870348
C	13.217565	1.911227	9.905734
H	13.070527	0.896105	10.264947
C	13.146166	2.191491	8.547502
H	12.927698	1.390814	7.845269
C	13.873229	5.374844	11.387923
H	13.487303	6.300237	10.941911
C	13.109369	5.137231	12.692435
H	12.048109	4.947533	12.507190
H	13.172007	6.020428	13.330165
H	13.520116	4.292907	13.257559
C	15.360269	5.616296	11.683664
H	15.838988	4.699618	12.047285
H	15.467367	6.385417	12.456727
H	15.900535	5.958655	10.798317
C	11.057459	12.013981	4.317611
C	12.188205	12.194380	3.484466
C	11.978447	12.600870	2.164545
H	12.831200	12.743404	1.507581
C	10.693482	12.807444	1.669892
H	10.551533	13.115579	0.637567
C	9.591252	12.603389	2.493647
H	8.591182	12.745233	2.093450
C	9.751904	12.204335	3.823714
C	13.590375	11.946636	4.019844
H	13.491774	11.247886	4.858693
C	14.199602	13.234610	4.592253
H	14.275060	14.005021	3.816103
H	15.206461	13.041218	4.979195
H	13.598386	13.634044	5.412929
C	14.533670	11.310781	2.996272
H	14.103228	10.406907	2.559160
H	15.476631	11.038213	3.482350
H	14.782168	12.001390	2.182388
C	8.554830	11.900581	4.702901
H	8.913591	11.903108	5.735610
C	8.023939	10.489765	4.419441
H	7.176358	10.248404	5.070093
H	8.793654	9.729357	4.584222
H	7.692821	10.398872	3.379075
C	7.438433	12.940499	4.586896
H	6.980897	12.938287	3.591911
H	7.814531	13.950568	4.779702
H	6.641729	12.725520	5.306706

C	10.940905	7.212894	10.021788
H	11.604098	6.364901	10.286954
N	11.196583	8.318944	10.970331
C	11.168491	7.990786	12.340392
C	10.099330	7.281092	12.958119
C	10.172110	6.949035	14.314290
H	9.345571	6.397485	14.759516
C	11.228994	7.369060	15.115329
H	11.259331	7.116163	16.171384
C	12.228602	8.150965	14.542263
H	13.053650	8.509959	15.153858
C	12.221983	8.453299	13.179302
C	8.817991	6.991323	12.224717
H	8.629801	7.752571	11.460987
H	7.971500	6.992826	12.919174
H	8.819951	6.019171	11.718436
C	13.365894	9.232236	12.595860
H	13.067584	10.244555	12.294052
H	13.763354	8.728991	11.708752
H	14.170230	9.358211	13.326276
Mg	9.448319	7.777267	8.398430
Mg	10.806630	10.208831	10.575353
N	7.799953	6.598267	8.146363
C	6.653788	6.848473	8.787139
C	6.406075	8.001213	9.555004
H	5.460216	8.007295	10.085363
C	7.213186	9.136703	9.785905
N	8.383855	9.324713	9.177105
C	5.542316	5.823698	8.738461
H	5.398480	5.444163	7.723320
H	4.600332	6.238517	9.100571
H	5.801252	4.961557	9.361472
C	6.707443	10.088880	10.846345
H	5.632731	10.255311	10.738733
H	7.217751	11.050082	10.856015
H	6.871065	9.620620	11.823775
N	9.134774	10.477957	9.486554
C	8.715375	11.657645	9.042250
C	9.264078	12.872458	9.508424
H	8.872563	13.764952	9.033116
C	10.093977	13.095702	10.621150
N	10.776716	12.137468	11.265816
C	7.560246	11.734800	8.078533
H	7.582206	10.895545	7.379258
H	7.584865	12.677348	7.529636
H	6.602119	11.684835	8.607048
C	10.197763	14.531798	11.081826
H	10.379178	14.596073	12.156724
H	9.293999	15.094142	10.840241
H	11.039949	15.015760	10.574675
C	7.914812	5.397233	7.382882
C	7.779734	5.470999	5.976897
C	7.896303	4.292136	5.235008
H	7.788771	4.324645	4.155940
C	8.165568	3.073073	5.850598
H	8.255437	2.169711	5.253677
C	8.356642	3.024738	7.225567
H	8.619031	2.082611	7.699167
C	8.245867	4.176648	8.009988
C	7.534816	6.808097	5.295265
H	8.106013	7.559561	5.856918
C	8.039456	6.842832	3.852174
H	9.089896	6.541914	3.795770
H	7.951455	7.856450	3.452738
H	7.452987	6.190961	3.195151
C	6.055384	7.213933	5.353859
H	5.427983	6.447833	4.884130
H	5.895816	8.156112	4.818139
H	5.715772	7.353684	6.382549
C	8.580842	4.115533	9.487619
H	8.292231	5.075370	9.927101
C	7.835983	3.011066	10.244060
H	6.752257	3.082575	10.108265
H	8.147123	2.015003	9.911122
H	8.050715	3.078449	11.315908
C	10.096897	3.958647	9.657198
H	10.638807	4.710708	9.076676
H	10.399030	4.065881	10.703438
H	10.440495	2.981821	9.303412
C	11.275925	12.357079	12.592579
C	10.488654	11.860286	13.664089
C	10.988980	11.945972	14.964303
H	10.399387	11.560980	15.789700
C	12.235101	12.507587	15.222135
H	12.611219	12.559490	16.240125
C	12.991029	13.004947	14.170331
H	13.960195	13.452139	14.374801
C	12.536049	12.942262	12.847778

C	9.094260	11.301712	13.422787
H	9.076015	10.838433	12.429159
C	8.065051	12.441571	13.384840
H	8.064509	12.984659	14.336580
H	7.057479	12.044856	13.218347
H	8.283333	13.154763	12.585590
C	8.678388	10.228389	14.428830
H	9.447319	9.460062	14.534738
H	7.757567	9.740531	14.091860
H	8.469794	10.654830	15.416538
C	13.420675	13.519769	11.755989
H	12.921820	13.342339	10.796052
C	14.801402	12.844537	11.722906
H	15.361222	13.158603	10.835407
H	14.728163	11.754678	11.715066
H	15.391920	13.124049	12.601747
C	13.604589	15.036920	11.919642
H	14.165374	15.262167	12.833197
H	12.649489	15.562679	11.984445
H	14.168102	15.448180	11.074986
O	10.590844	7.986488	6.826213
C	10.987813	6.747985	6.262064
H	10.379844	5.878996	6.506212
C	11.642763	6.797048	5.048061
H	11.897237	5.855793	4.559726
N	11.958113	7.966003	4.459482
C	12.721879	7.955557	3.272368
C	14.029975	7.423881	3.239575
C	14.761282	7.498970	2.048906
H	15.773868	7.101075	2.030504
C	14.223578	8.078934	0.904863
H	14.808753	8.134069	-0.009018
C	12.928500	8.592753	0.942740
H	12.496391	9.043626	0.052332
C	12.166221	8.539814	2.111091
C	14.649675	6.796267	4.458732
H	14.517397	5.707881	4.466104
H	14.202632	7.171534	5.379754
H	15.725259	6.990876	4.501326
C	10.767067	9.083866	2.137751
H	10.690406	9.964010	2.782682
H	10.064464	8.342967	2.527708
H	10.441970	9.388700	1.139629
C	13.341582	3.489346	8.064371
C	13.164090	3.760441	6.579784
H	13.274984	4.839168	6.426924
C	11.749260	3.364777	6.136390
H	10.985484	3.828086	6.763770
H	11.562662	3.659827	5.098359
H	11.599214	2.281274	6.196723
C	14.206980	3.043713	5.713854
H	15.226561	3.346034	5.967117
H	14.142204	1.956681	5.836372
H	14.043252	3.267262	4.653561
H	9.979202	6.699421	10.229883

INT-7.log

SCF (B3PW91) = -4125.16223722
E(SCF)+ZPE(0 K)= -4123.262385
H(298 K)= -4123.149755
G(298 K)= -4123.402328
Lowest Frequency = 17.8707cm-1

Mg	12.711395	6.907616	8.128339
Mg	11.439599	9.349739	6.090350
N	14.351234	5.625482	8.177024
C	15.534215	5.823921	7.595497
C	15.908871	7.012710	6.939065
H	16.879634	6.999437	6.455962
C	15.153164	8.185101	6.756240
N	13.937085	8.304713	7.266415
C	16.553821	4.705931	7.543195
H	16.404087	3.970341	8.335010
H	17.572139	5.097443	7.603811
H	16.457227	4.184690	6.584059
C	15.720816	9.270637	5.886989
H	15.240952	9.194943	4.904517
H	16.798141	9.159732	5.753809
H	15.509245	10.266973	6.277945
N	13.197519	9.496817	7.175169
C	13.363703	10.403857	8.104547
C	12.409751	11.481689	8.279904
H	12.717946	12.202564	9.031699
C	11.712247	12.091648	7.184930
N	11.399613	11.438321	6.072239
C	14.567296	10.388319	9.006716
H	14.956456	9.378093	9.143708

H	14.341164	10.825838	9.975869
H	15.354735	10.998956	8.548485
C	11.425503	13.571166	7.282829
H	12.042860	14.117713	6.562506
H	11.635963	13.949589	8.282764
H	10.380855	13.780257	7.044770
C	14.095776	4.415268	8.887950
C	14.304110	4.392489	10.286886
C	13.987232	3.227794	10.988854
H	14.142488	3.193839	12.063203
C	13.457882	2.115442	10.340134
H	13.209694	1.221220	10.905379
C	13.235458	2.159180	8.968715
H	12.807527	1.295403	8.466046
C	14.874476	5.604850	11.003938
H	14.651160	6.473874	10.374375
C	14.250387	5.846461	12.383055
H	13.158295	5.834754	12.343025
H	14.560964	6.823320	12.769411
H	14.572919	5.094532	13.112237
C	16.403076	5.512036	11.116070
H	16.694217	4.620413	11.683411
H	16.805431	6.390092	11.634311
H	16.875892	5.455465	10.132412
C	11.236359	12.194862	4.863488
C	12.389180	12.389055	4.060089
C	12.244816	13.058311	2.842873
H	13.115416	13.219105	2.216219
C	11.003466	13.502098	2.402401
H	10.911390	14.007245	1.444888
C	9.881636	13.283788	3.188669
H	8.911888	13.614730	2.830748
C	9.975480	12.641362	4.428270
C	13.755661	11.868420	4.479964
H	13.586319	10.972884	5.086595
C	14.498358	12.879000	5.367945
H	14.590325	13.845652	4.859976
H	15.509584	12.521349	5.591989
H	13.987926	13.038861	6.320482
C	14.633587	11.464921	3.291810
H	14.123198	10.748889	2.641314
H	15.556033	11.000637	3.651727
H	14.935104	12.329869	2.690872
C	8.714163	12.376778	5.226659

H	9.003668	12.181398	6.265505
C	8.024485	11.115226	4.688438
H	7.075328	10.928531	5.197806
H	8.659067	10.234244	4.827151
H	7.824643	11.210471	3.615719
C	7.770981	13.585365	5.216253
H	7.370458	13.782828	4.217182
H	8.284864	14.489639	5.557543
H	6.908341	13.419197	5.866981
C	12.270304	7.963220	10.040884
H	12.900022	8.772219	9.648802
N	10.970077	8.474704	10.528627
C	10.920272	8.359660	11.964054
C	9.941562	7.574979	12.629400
C	10.072149	7.313022	13.997599
H	9.331201	6.674693	14.473241
C	11.095841	7.863362	14.758342
H	11.175310	7.649598	15.820209
C	11.980683	8.737959	14.140258
H	12.746611	9.240767	14.726096
C	11.911124	8.992592	12.768779
C	8.730631	7.032803	11.933958
H	8.963991	6.281028	11.174257
H	8.166371	7.824050	11.442218
H	8.055286	6.552567	12.646929
C	12.930791	9.947999	12.210830
H	12.552726	10.559013	11.380618
H	13.812575	9.429378	11.821453
H	13.242354	10.665013	12.974613
Mg	9.562426	7.627221	8.964856
Mg	10.733193	10.517885	9.952061
N	7.870488	6.403347	8.458216
C	6.654843	6.641782	8.951943
C	6.322021	7.792752	9.692034
H	5.358956	7.774220	10.192343
C	7.035460	8.998423	9.806353
N	8.210724	9.184512	9.199629
C	5.492109	5.699443	8.700219
H	5.799700	4.741132	8.285020
H	4.792825	6.157801	7.993471
H	4.943506	5.526230	9.630876
C	6.435487	10.014491	10.753635
H	6.439709	9.578246	11.758541
H	5.392909	10.228176	10.500638

H	6.988323	10.950032	10.794305
N	8.827431	10.435896	9.251710
C	8.191793	11.510541	8.801116
C	8.649820	12.815632	9.104036
H	8.120666	13.620168	8.603591
C	9.503265	13.203269	10.154067
N	10.321520	12.367655	10.810855
C	6.917963	11.375675	8.007580
H	6.897921	10.432449	7.457325
H	6.826295	12.210096	7.315263
H	6.033967	11.403800	8.650422
C	9.456117	14.667147	10.534166
H	9.672551	14.810372	11.595074
H	8.483455	15.108633	10.308865
H	10.214028	15.218440	9.965740
C	7.996719	5.321637	7.536765
C	7.851690	5.601592	6.154775
C	7.932060	4.537769	5.253299
H	7.820558	4.723195	4.190389
C	8.170381	3.235744	5.686630
H	8.225029	2.423678	4.966651
C	8.366268	2.986576	7.037502
H	8.580815	1.974816	7.369707
C	8.299584	4.017095	7.982858
C	7.680046	7.034525	5.661220
H	8.358828	7.651837	6.266581
C	8.081195	7.206813	4.194835
H	9.092346	6.838312	4.003626
H	8.051156	8.267535	3.931306
H	7.385923	6.695662	3.519120
C	6.260829	7.589977	5.860713
H	5.515097	6.920856	5.416775
H	6.169980	8.565442	5.369472
H	6.021084	7.733467	6.914923
C	8.540338	3.699734	9.449459
H	8.685005	4.655010	9.963237
C	7.338785	3.001188	10.101900
H	6.443741	3.625897	10.091524
H	7.104984	2.066864	9.578323
H	7.565054	2.754497	11.145194
C	9.801624	2.851413	9.650981
H	10.665845	3.267297	9.131703
H	10.056527	2.793517	10.713241
H	9.658613	1.827011	9.289251
C	10.833409	12.697688	12.104940
C	10.156329	12.157956	13.227707
C	10.714130	12.325556	14.496257
H	10.212802	11.900787	15.359645
C	11.905587	13.022014	14.676550
H	12.327099	13.136656	15.671516
C	12.542881	13.579508	13.576421
H	13.464046	14.139476	13.717139
C	12.025662	13.430778	12.284514
C	8.813918	11.467011	13.060800
H	8.839381	10.926821	12.106934
C	7.693836	12.516849	12.973189
H	7.704150	13.154343	13.864566
H	6.713248	12.031913	12.917356
H	7.802236	13.157184	12.094583
C	8.496708	10.456346	14.161203
H	9.329151	9.771696	14.328010
H	7.622466	9.861192	13.877879
H	8.254478	10.950915	15.109329
C	12.772231	14.058856	11.124408
H	12.222490	13.796863	10.215222
C	14.194975	13.498898	11.004175
H	14.657929	13.795605	10.056655
H	14.195853	12.408969	11.063911
H	14.832040	13.865946	11.815865
C	12.822057	15.589414	11.228673
H	13.413183	15.900384	12.096876
H	11.825521	16.023649	11.337427
H	13.290708	16.020786	10.337070
O	10.689311	7.894273	7.304181
C	10.935980	6.676079	6.630224
H	10.378040	5.809277	6.971776
C	11.422693	6.708005	5.341127
H	11.534491	5.752422	4.829881
N	11.727233	7.865444	4.715879
C	12.432638	7.872804	3.499173
C	13.597363	7.096709	3.273711
C	14.290630	7.245889	2.066855
H	15.192131	6.656367	1.911816
C	13.865437	8.127633	1.080182
H	14.425091	8.232115	0.154909
C	12.707310	8.871026	1.295298
H	12.348410	9.553812	0.528441
C	11.984710	8.754645	2.482945

C 14.131808 6.119913 4.288006
H 13.738619 5.109303 4.121132
H 13.874570 6.406224 5.307851
H 15.221826 6.058461 4.233502
C 10.719461 9.540090 2.668755
H 10.811172 10.286691 3.462275
H 9.887484 8.885630 2.938258
H 10.456554 10.090582 1.761820
C 13.542554 3.300876 8.221870
C 13.281827 3.322435 6.726391
H 13.572979 4.312251 6.363375
C 11.795477 3.132685 6.407011
H 11.176464 3.872013 6.918340
H 11.611071 3.227361 5.331167
H 11.437871 2.144971 6.717173
C 14.126456 2.276066 5.987853
H 15.189919 2.379155 6.220676
H 13.824365 1.259875 6.265680
H 14.000700 2.375303 4.903834
C 10.969918 5.988531 9.853527
O 11.292639 5.314257 10.737147
H 12.835580 7.494983 10.848764

INT-8.log

SCF (B3PW91) = -4125.18471832
E(SCF)+ZPE(0 K)= -4123.281712
H(298 K)= -4123.169790
G(298 K)= -4123.421888
Lowest Frequency = 11.1001cm-1

Mg 12.688739 6.403390 8.058722
Mg 11.483173 9.267776 6.300736
N 14.345141 5.171387 7.901676
C 15.538468 5.548622 7.445207
C 15.899594 6.878774 7.161728
H 16.896962 7.006446 6.755137
C 15.123901 8.052993 7.184230
N 13.865846 8.084898 7.620388
C 16.585160 4.508740 7.103877
H 16.478642 3.598449 7.695145
H 17.595393 4.904177 7.231037
H 16.466758 4.232874 6.049734
C 15.767063 9.263294 6.557278
H 15.627477 9.188496 5.473453
H 16.840471 9.283597 6.756211
H 15.329650 10.205628 6.876811
N 13.130547 9.285970 7.574524
C 13.320887 10.227716 8.461036
C 12.439415 11.383428 8.527364
H 12.793866 12.121547 9.242032
C 11.851895 11.999682 7.368567
N 11.538692 11.326202 6.271901
C 14.475202 10.216055 9.430128
H 14.943345 9.234257 9.503816
H 14.151924 10.529555 10.422274
H 15.228275 10.937746 9.094208
C 11.650822 13.495515 7.388293
H 12.148391 13.951217 6.527356
H 12.038278 13.948239 8.299602
H 10.585080 13.726394 7.309886
C 14.135083 3.803078 8.256985
C 14.381602 3.403050 9.592018
C 14.153672 2.068319 9.935367
H 14.340139 1.740705 10.953385
C 13.680937 1.149504 9.002157
H 13.510013 0.116546 9.293097
C 13.412725 1.564126 7.704075
H 13.024116 0.851503 6.980790
C 14.894338 4.395893 10.623661
H 14.518324 5.380843 10.324215
C 14.379255 4.113685 12.037204
H 13.293750 3.998924 12.044330
H 14.630508 4.951701 12.695460
H 14.839171 3.215793 12.466576
C 16.428930 4.468079 10.629810
H 16.860732 3.480112 10.827720
H 16.773488 5.150664 11.415007
H 16.827601 4.828485 9.679603
C 11.391175 12.025945 5.028368
C 12.529632 12.103433 4.187919
C 12.395113 12.717889 2.941140
H 13.255489 12.785178 2.284208
C 11.174367 13.227096 2.513529
H 11.088982 13.693065 1.535792
C 10.062393 13.118322 3.335896
H 9.104295 13.488717 2.984559
C 10.146184 12.520594 4.598372

C	13.874880	11.538640	4.615582
H	13.675362	10.680562	5.266623
C	14.664773	12.564717	5.442833
H	14.780028	13.500067	4.883857
H	15.666572	12.186149	5.672810
H	14.169392	12.793019	6.390296
C	14.717069	11.036335	3.440744
H	14.157122	10.336085	2.816314
H	15.608145	10.522636	3.813605
H	15.073216	11.859384	2.811338
C	8.880609	12.326076	5.410041
H	9.165625	12.104412	6.443894
C	8.118940	11.110214	4.859056
H	7.148082	10.984853	5.344194
H	8.692741	10.190907	5.013689
H	7.950149	11.213858	3.781931
C	8.009296	13.586489	5.425409
H	7.607635	13.819963	4.434447
H	8.579508	14.454844	5.770590
H	7.149702	13.461341	6.089682
C	12.288131	7.970396	10.709332
H	12.940971	8.495555	10.013950
N	10.955680	8.638419	10.783918
C	10.575896	8.707669	12.180543
C	9.606059	7.853777	12.760066
C	9.307067	7.963222	14.122387
H	8.569362	7.286194	14.546786
C	9.953262	8.876812	14.944790
H	9.710118	8.938453	16.001867
C	10.918190	9.707262	14.392225
H	11.431870	10.436403	15.013257
C	11.245951	9.628258	13.036020
C	8.946036	6.753729	11.987478
H	9.677770	6.189388	11.409276
H	8.153503	7.117834	11.329962
H	8.471457	6.040027	12.666063
C	12.411283	10.472630	12.588767
H	12.347147	10.874938	11.568933
H	13.342179	9.894348	12.598924
H	12.532050	11.342616	13.235946
Mg	9.672894	7.693603	9.115288
Mg	10.701092	10.669142	10.161222
N	8.045896	6.397797	8.714845
C	6.827507	6.569789	9.239187

C	6.445781	7.714739	9.961230
H	5.503638	7.656677	10.495072
C	7.111902	8.955324	10.045532
N	8.258564	9.179215	9.407529
C	5.769608	5.496378	9.073627
H	6.172137	4.501757	9.278020
H	5.399876	5.471319	8.044418
H	4.920626	5.682297	9.734357
C	6.493827	9.965783	10.982863
H	6.663592	9.612201	12.006079
H	5.413047	10.036154	10.832721
H	6.929268	10.958537	10.900808
N	8.819375	10.460617	9.412546
C	8.162751	11.463478	8.848707
C	8.602478	12.803079	8.997334
H	8.083112	13.532430	8.386101
C	9.432882	13.329312	10.000438
N	10.197326	12.592448	10.825663
C	6.892918	11.212090	8.078891
H	6.930756	10.246733	7.568006
H	6.725698	12.009404	7.356573
H	6.022564	11.196505	8.741755
C	9.432664	14.837428	10.131953
H	9.520051	15.147768	11.175431
H	8.531152	15.281219	9.705689
H	10.296915	15.245768	9.596150
C	8.159284	5.328541	7.782408
C	7.738670	5.555196	6.451134
C	7.733184	4.470403	5.570047
H	7.398525	4.609560	4.546554
C	8.181868	3.215266	5.972496
H	8.166829	2.382367	5.274709
C	8.714139	3.046375	7.246975
H	9.140180	2.087324	7.524121
C	8.737286	4.100309	8.166195
C	7.429768	6.969395	5.970009
H	8.035272	7.636642	6.597915
C	7.867561	7.181774	4.517828
H	8.906861	6.875567	4.369590
H	7.778226	8.240488	4.256728
H	7.238461	6.627954	3.811978
C	5.963273	7.394170	6.133391
H	5.290953	6.675818	5.649853
H	5.802549	8.371139	5.662527

H	5.679064	7.489055	7.183250
C	9.419081	3.958121	9.516331
H	9.865112	4.932598	9.745636
C	8.440915	3.630454	10.649901
H	7.701679	4.422628	10.785012
H	7.912618	2.691952	10.444265
H	8.984044	3.518343	11.594081
C	10.577638	2.961096	9.507330
H	11.266389	3.141881	8.677637
H	11.152687	3.065021	10.429835
H	10.232547	1.923185	9.436057
C	10.589650	13.139338	12.089263
C	9.749871	12.892858	13.206389
C	10.178706	13.303076	14.470710
H	9.553083	13.106837	15.335193
C	11.397709	13.951110	14.650709
H	11.714393	14.253927	15.645096
C	12.196572	14.219923	13.547512
H	13.139439	14.744579	13.681152
C	11.808827	13.828223	12.261643
C	8.396940	12.222434	13.027563
H	8.537801	11.437293	12.274606
C	7.349251	13.218336	12.499726
H	7.273485	14.078471	13.174664
H	6.363564	12.741672	12.452926
H	7.583545	13.583860	11.499896
C	7.843933	11.573055	14.297024
H	8.572641	10.913652	14.768026
H	6.962233	10.973953	14.047468
H	7.522910	12.325543	15.027176
C	12.706698	14.166799	11.089648
H	12.210563	13.767176	10.201114
C	14.087634	13.510380	11.206096
H	14.670286	13.663108	10.290683
H	14.009849	12.436288	11.385653
H	14.657209	13.937891	12.038032
C	12.863142	15.682653	10.906749
H	13.416433	16.121904	11.743885
H	11.894667	16.185944	10.852768
H	13.419553	15.905117	9.989207
O	10.614571	7.797086	7.340852
C	10.922370	6.625770	6.612217
H	10.320850	5.748817	6.836159
C	11.445883	6.751850	5.332918
H	11.590957	5.833093	4.763498
N	11.766212	7.940196	4.789313
C	12.395478	8.001173	3.525039
C	13.626764	7.362105	3.257347
C	14.216652	7.514057	1.996662
H	15.170171	7.025261	1.806906
C	13.620574	8.279533	1.002529
H	14.097102	8.391460	0.032568
C	12.404614	8.905695	1.268860
H	11.921208	9.504417	0.500450
C	11.782602	8.776101	2.511010
C	14.339119	6.531353	4.286843
H	14.217129	5.460853	4.087755
H	13.970522	6.722093	5.293740
H	15.413619	6.736318	4.286856
C	10.464113	9.441637	2.765345
H	10.562070	10.238049	3.506571
H	9.730531	8.730490	3.151544
H	10.067306	9.899804	1.855698
C	13.625583	2.889579	7.311585
C	13.292295	3.317825	5.895023
H	13.524424	4.384148	5.820715
C	11.798488	3.147128	5.600551
H	11.182805	3.715491	6.301189
H	11.557653	3.483628	4.585843
H	11.490673	2.098972	5.680152
C	14.137769	2.572195	4.855354
H	15.207597	2.674612	5.057965
H	13.903150	1.501885	4.850682
H	13.940253	2.957552	3.848631
C	12.028463	6.548547	10.178286
O	11.866043	5.659787	11.021077
H	12.780051	7.918279	11.688871

INT-9.log

SCF (B3PW91) = -4125.23188811

E(SCF)+ZPE(0 K)= -4123.331225

H(298 K)= -4123.218828

G(298 K)= -4123.474605

Lowest Frequency = 14.5297cm-1

Mg	12.554124	6.668027	7.898073
Mg	11.629507	9.310286	5.776916

N	14.139313	5.378585	8.126763
C	15.395669	5.643102	7.746141
C	15.848605	6.888516	7.275807
H	16.891314	6.925581	6.981498
C	15.120891	8.083895	7.079328
N	13.830454	8.160002	7.356803
C	16.423021	4.531331	7.748467
H	16.203245	3.769106	8.498483
H	17.428779	4.921555	7.918615
H	16.418419	4.038873	6.769831
C	15.826892	9.282370	6.503622
H	16.869946	9.060179	6.273936
H	15.791434	10.136210	7.186459
H	15.316235	9.590180	5.585389
N	13.141286	9.376254	7.175651
C	13.139013	10.245339	8.173114
C	12.361171	11.437095	8.146175
H	12.483032	12.079740	9.010003
C	11.688422	12.007717	7.052094
N	11.404083	11.334394	5.928307
C	13.984413	10.028510	9.402913
H	14.394966	9.020526	9.437003
H	13.387238	10.193232	10.307718
H	14.814332	10.741834	9.414291
C	11.269748	13.454553	7.180771
H	11.231397	13.947247	6.206827
H	11.944655	14.011143	7.834706
H	10.266828	13.494463	7.624178
C	13.788493	4.092488	8.634136
C	13.696078	3.915580	10.036855
C	13.254159	2.685263	10.528142
H	13.179540	2.532954	11.600317
C	12.884809	1.654598	9.667664
H	12.534854	0.707726	10.069820
C	12.957161	1.847229	8.293209
H	12.658371	1.045363	7.622487
C	14.060534	5.051967	10.976622
H	13.778902	5.978248	10.461487
C	13.317989	5.011380	12.314685
H	12.242083	4.862633	12.181458
H	13.459426	5.953381	12.853615
H	13.689607	4.207991	12.960689
C	15.574679	5.127994	11.220696
H	15.941244	4.191937	11.657037
H	15.806890	5.941875	11.916526
H	16.124364	5.312751	10.295690
C	11.194026	12.032406	4.703680
C	12.330668	12.307437	3.905314
C	12.139197	12.889368	2.649526
H	13.000950	13.111308	2.026865
C	10.863029	13.178362	2.179442
H	10.730921	13.622415	1.196556
C	9.754199	12.894757	2.970187
H	8.763133	13.119949	2.589242
C	9.895389	12.325935	4.239699
C	13.732858	11.994556	4.403375
H	13.636776	11.267910	5.217943
C	14.381143	13.249928	5.003417
H	14.493292	14.030378	4.241806
H	15.374667	13.017572	5.402899
H	13.774474	13.657577	5.817509
C	14.627341	11.365058	3.330674
H	14.171753	10.466137	2.904827
H	15.593257	11.086953	3.765831
H	14.837116	12.059134	2.509588
C	8.677330	11.961785	5.070484
H	8.971887	11.994318	6.126489
C	8.246349	10.525905	4.743306
H	7.345130	10.236734	5.295925
H	9.025103	9.794979	4.982262
H	8.029221	10.429098	3.674540
C	7.507787	12.930010	4.876825
H	7.102924	12.881756	3.860512
H	7.809088	13.963826	5.072562
H	6.688129	12.675404	5.553266
C	11.994281	7.984511	10.961410
H	13.095141	7.872050	11.006613
N	11.486592	9.128038	11.665795
C	11.388701	8.864783	13.046061
C	10.383978	7.994986	13.543633
C	10.354963	7.681156	14.906070
H	9.582053	7.011830	15.279589
C	11.279526	8.232355	15.788998
H	11.241228	7.986662	16.847049
C	12.243309	9.117597	15.306867
H	12.965239	9.557829	15.991831
C	12.314990	9.435166	13.948251
C	9.343679	7.417538	12.618274

H	9.639885	6.439589	12.215247
H	9.184016	8.068838	11.752504
H	8.388540	7.276084	13.133180
C	13.385176	10.346986	13.420117
H	12.946191	11.234451	12.957451
H	13.977818	9.846014	12.646227
H	14.057184	10.678153	14.217098
Mg	9.128985	8.105181	7.997446
Mg	10.431191	10.387815	10.503130
N	7.721130	6.595251	8.072818
C	6.607999	6.636078	8.821403
C	6.292767	7.663605	9.722302
H	5.412445	7.499224	10.332671
C	6.972773	8.878624	9.960679
N	7.969214	9.300468	9.192271
C	5.620042	5.492960	8.732980
H	6.034199	4.574413	9.161140
H	5.387267	5.268498	7.687918
H	4.693568	5.729024	9.258476
C	6.549452	9.698957	11.153732
H	5.736644	9.214061	11.695977
H	6.239677	10.711342	10.881998
H	7.402814	9.814822	11.829729
N	8.589857	10.516988	9.564920
C	8.044764	11.625932	9.081871
C	8.499250	12.909745	9.438261
H	7.971481	13.734857	8.973032
C	9.403457	13.264056	10.457692
N	10.203285	12.402402	11.093947
C	6.853076	11.536975	8.163017
H	7.035749	10.815338	7.362877
H	6.623871	12.511198	7.731688
H	5.966164	11.183180	8.698062
C	9.395389	14.734287	10.822239
H	9.702198	14.902708	11.855422
H	8.403798	15.166088	10.673411
H	10.093683	15.274959	10.174219
C	7.954703	5.427112	7.285386
C	7.721298	5.464807	5.894323
C	8.023002	4.332336	5.129660
H	7.839347	4.349827	4.058773
C	8.560469	3.192159	5.711471
H	8.805520	2.328308	5.100121
C	8.798192	3.167958	7.082662

H	9.240452	2.282808	7.529899
C	8.494084	4.265750	7.890205
C	7.156281	6.694779	5.206316
H	7.101729	7.496227	5.952201
C	8.066690	7.170065	4.068324
H	9.062817	7.413874	4.447019
H	7.647364	8.061540	3.592660
H	8.173835	6.406087	3.291152
C	5.727932	6.441954	4.705497
H	5.710730	5.642709	3.956033
H	5.316153	7.345453	4.242230
H	5.063779	6.146957	5.523903
C	8.799460	4.215094	9.378534
H	8.190728	4.974189	9.879523
C	8.475200	2.862238	10.020481
H	7.457832	2.535441	9.781681
H	9.168025	2.080207	9.691894
H	8.565757	2.934845	11.109138
C	10.263765	4.586805	9.619293
H	10.450979	5.614948	9.294163
H	10.521994	4.533615	10.681556
H	10.942473	3.919690	9.076446
C	10.858404	12.828058	12.292673
C	10.312776	12.395844	13.527873
C	10.961196	12.758853	14.710050
H	10.565112	12.422845	15.661877
C	12.114691	13.536762	14.694946
H	12.603724	13.805569	15.627260
C	12.638656	13.959304	13.481602
H	13.544753	14.559519	13.468533
C	12.032417	13.612884	12.269105
C	9.028548	11.580311	13.571193
H	9.101411	10.820172	12.781945
C	7.797804	12.448982	13.268028
H	7.732222	13.272982	13.987439
H	6.882487	11.852807	13.353682
H	7.826191	12.869206	12.261189
C	8.817904	10.829072	14.884863
H	9.703130	10.259039	15.171064
H	7.987598	10.124019	14.775268
H	8.557389	11.513459	15.701143
C	12.683805	14.060630	10.974072
H	12.029932	13.746480	10.154586
C	14.041457	13.368674	10.793079

H	14.448902	13.556073	9.793161
H	13.952506	12.290438	10.934088
H	14.765743	13.735866	11.528425
C	12.854903	15.583058	10.888733
H	13.580916	15.940977	11.626716
H	11.915340	16.111204	11.069716
H	13.226218	15.870391	9.898727
O	10.388832	7.935447	6.535033
C	10.945556	6.691934	6.165633
H	10.322018	5.819131	6.360302
C	11.685206	6.696590	4.979230
H	11.984781	5.739727	4.552005
N	12.020884	7.847312	4.390016
C	12.763713	7.878458	3.193128
C	14.015093	7.239205	3.048045
C	14.722632	7.408381	1.852371
H	15.693875	6.928792	1.749221
C	14.217206	8.178029	0.810486
H	14.786385	8.301057	-0.106790
C	12.972375	8.787415	0.953741
H	12.560980	9.381447	0.140940
C	12.234409	8.649409	2.129991
C	14.612810	6.395265	4.141708
H	14.381583	5.331851	3.999788
H	14.242359	6.678958	5.127132
H	15.702216	6.488489	4.160114
C	10.888366	9.306149	2.264949
H	10.925073	10.181538	2.922812
H	10.150854	8.619194	2.689105
H	10.525888	9.659440	1.295700
C	13.408297	3.056884	7.753658
C	13.450823	3.237901	6.246948
H	13.835211	4.243555	6.048350
C	12.043562	3.152035	5.647573
H	11.364166	3.866452	6.117311
H	12.062334	3.349835	4.569802
H	11.610322	2.156321	5.791651
C	14.385220	2.225992	5.571393
H	15.385694	2.241470	6.012689
H	13.998200	1.205433	5.668067
H	14.477867	2.444537	4.501856
C	11.571338	7.840556	9.538811
O	10.666117	8.690593	9.224673
H	11.640284	7.010625	11.362850

TS1.log

SCF (B3PW91) = -3898.47144866
E(SCF)+ZPE(0 K)= -3896.601654
H(298 K)= -3896.492379
G(298 K)= -3896.740807
Lowest Frequency = -144.5790cm-1

Mg	3.810087	10.534474	14.113640
Mg	5.453764	12.058611	10.895787
Mg	4.318248	8.303657	11.219235
Mg	2.140374	10.556528	10.656419
H	3.976875	9.360600	12.694232
H	3.770603	12.137352	10.365938
N	4.358823	9.954349	16.010519
C	5.626618	9.851235	16.422824
C	6.719639	10.430491	15.762260
H	7.694665	10.221445	16.185253
C	6.714128	11.262015	14.624747
N	5.608423	11.527622	13.936499
C	5.930070	9.041555	17.664543
H	5.320707	9.365109	18.512396
H	6.983832	9.108300	17.939502
H	5.680744	7.988978	17.488345
C	8.034234	11.845353	14.185608
H	8.223353	11.591431	13.139822
H	8.852909	11.463307	14.796941
H	8.043320	12.937508	14.238955
N	5.726535	12.457243	12.883191
C	5.675830	13.744457	13.223841
C	5.918595	14.789509	12.302339
H	5.749840	15.789700	12.683414
C	6.531661	14.712263	11.038157
N	6.744243	13.564187	10.378909
C	5.392690	14.126678	14.656956
H	4.634553	13.471554	15.094918
H	5.061497	15.164378	14.720297
H	6.290209	14.017284	15.275423
C	6.977389	16.015915	10.415346
H	8.001331	15.940827	10.039715
H	6.920188	16.841452	11.126291
H	6.341872	16.253139	9.555794
C	3.334212	9.507931	16.892079

C	2.750414	10.448728	17.773367	H	10.623587	11.130788	10.304038
C	1.730150	10.008194	18.619936	H	10.853218	11.624145	11.989494
H	1.260984	10.707850	19.304002	H	11.606545	12.548441	10.691162
C	1.285592	8.688061	18.589816	C	5.732912	14.171280	7.699062
H	0.490050	8.368624	19.257782	H	5.260393	14.351195	8.669296
C	1.839789	7.788872	17.686443	C	5.003097	12.981889	7.071148
H	1.460358	6.771496	17.641005	H	5.432692	12.721929	6.097133
C	2.863630	8.181069	16.818134	H	3.937309	13.196362	6.938720
C	3.202369	11.904546	17.758891	H	5.097131	12.106234	7.715924
H	3.374993	12.158532	16.703607	C	5.568498	15.434671	6.848003
C	2.147240	12.870592	18.298474	H	6.133240	16.276397	7.261050
H	1.172312	12.701428	17.840358	H	4.513808	15.724257	6.795280
H	2.444955	13.901169	18.081376	H	5.914658	15.276130	5.820933
H	2.043467	12.785308	19.386948	N	4.917973	6.340815	11.306558
C	4.524507	12.134952	18.508194	C	4.070066	5.330215	11.557302
H	4.452885	11.764766	19.537406	C	2.674120	5.452126	11.528596
H	4.744697	13.207603	18.552431	H	2.112045	4.582832	11.851363
H	5.370868	11.649921	18.020527	C	1.894807	6.528434	11.029724
C	3.386185	7.222796	15.761326	N	2.448097	7.650473	10.603222
H	4.290594	7.664984	15.330977	C	4.631072	3.963056	11.880389
C	3.760815	5.850306	16.328760	H	5.362338	3.659813	11.124909
H	4.487968	5.934478	17.143015	H	3.843254	3.209585	11.927467
H	4.196309	5.223698	15.543805	H	5.158359	3.973844	12.839612
H	2.885275	5.319953	16.718687	C	0.397807	6.349187	10.976919
C	2.368889	7.070430	14.621804	H	-0.101276	7.312775	11.105688
H	1.416866	6.679646	14.999524	H	0.071429	5.656350	11.756122
H	2.740166	6.385264	13.853347	H	0.073708	5.940501	10.013894
H	2.160199	8.022504	14.123852	N	1.646659	8.617555	9.968424
C	7.642558	13.555136	9.269997	C	1.406057	8.448507	8.673686
C	8.995133	13.213764	9.507713	C	0.738011	9.421068	7.886238
C	9.874170	13.179920	8.423706	H	0.502498	9.116127	6.872275
H	10.916628	12.923504	8.585669	C	0.266864	10.685510	8.280162
C	9.433585	13.447779	7.130333	N	0.632043	11.283109	9.415949
H	10.130790	13.405231	6.297813	C	1.870317	7.191951	7.973244
C	8.098122	13.758405	6.907463	H	2.875977	6.922509	8.310092
H	7.754261	13.954964	5.895308	H	1.874223	7.328989	6.890429
C	7.186024	13.826924	7.965534	H	1.228448	6.335848	8.204765
C	9.457526	12.878828	10.916032	C	-0.725757	11.371191	7.368014
H	8.625488	12.331027	11.374216	H	-1.743028	11.248734	7.757890
C	9.702319	14.130574	11.773528	H	-0.701011	10.948143	6.361469
H	10.431731	14.789551	11.289117	H	-0.536860	12.446321	7.313572
H	10.103550	13.844489	12.752373	C	6.308901	6.075678	11.164149
H	8.786044	14.695442	11.949912	C	6.855436	5.997661	9.858278
C	10.703667	11.992784	10.969186	C	8.236594	5.829383	9.726761

H	8.679708	5.770979	8.738847	H	-1.775091	10.535100	12.793319
C	9.068064	5.752312	10.840642	C	1.721459	14.011754	9.243584
H	10.139977	5.628404	10.711814	H	2.216184	13.067468	8.987334
C	8.522423	5.846615	12.113849	C	2.694648	14.811956	10.113849
H	9.176351	5.807628	12.980794	H	2.283361	15.784027	10.408285
C	7.145012	6.007085	12.298357	H	3.621122	15.004185	9.564507
C	5.961717	6.122168	8.630813	H	2.964293	14.256726	11.014615
H	5.302279	6.983393	8.811438	C	1.422641	14.762972	7.938604
C	6.739466	6.395924	7.343106	H	0.781638	14.176173	7.274012
H	7.307791	5.513264	7.026505	H	2.349490	14.989157	7.400022
H	7.429619	7.235496	7.449853	H	0.912484	15.711392	8.143214
H	6.041512	6.637085	6.535733	C	1.531469	11.407275	12.567829
C	5.061330	4.893917	8.423713	H	0.590737	11.982100	12.441517
H	5.666680	3.982777	8.356817	C	4.084944	10.214349	9.712562
H	4.501119	4.993698	7.487097	H	3.991003	10.726305	8.741537
H	4.334625	4.772634	9.228054	N	5.277141	9.652395	9.944066
C	6.593203	6.187495	13.699598	C	6.345624	9.687447	9.003434
H	5.504690	6.078402	13.652908	C	7.651348	9.671932	9.542945
C	7.131567	5.148490	14.689686	C	8.738333	9.772766	8.676119
H	6.979572	4.126830	14.326725	H	9.743983	9.755185	9.082981
H	6.625365	5.248629	15.654819	C	8.550787	9.889129	7.302114
H	8.203721	5.279407	14.871365	H	9.405392	10.002293	6.642471
C	6.879564	7.605221	14.201168	C	7.266296	9.814646	6.778570
H	7.950126	7.833923	14.158717	H	7.121313	9.823051	5.701016
H	6.558839	7.717559	15.238462	C	6.145490	9.670009	7.603272
H	6.352311	8.365016	13.614612	C	7.868912	9.480455	11.020810
C	-0.117754	12.369007	9.935413	H	7.242366	10.124032	11.648009
C	-1.349553	12.093302	10.581759	H	8.910060	9.666266	11.292592
C	-1.994351	13.124109	11.268475	H	7.625651	8.451435	11.307791
H	-2.922653	12.918119	11.793575	C	4.816416	9.377135	6.949693
C	-1.458141	14.407935	11.314074	H	4.166197	8.784336	7.598212
H	-1.970225	15.195508	11.859958	H	4.982590	8.805478	6.031848
C	-0.263980	14.673523	10.654988	H	4.258412	10.277110	6.671434
H	0.142678	15.681199	10.676265	N	1.934111	11.438677	13.809506
C	0.429031	13.670934	9.966680	C	1.078523	12.163945	14.713786
C	-1.920967	10.682343	10.622478	C	0.147069	11.440205	15.478261
H	-1.498922	10.114168	9.789032	C	0.186244	14.242262	15.534794
C	-3.445267	10.650450	10.468762	C	-0.768111	12.152000	16.257345
H	-3.956402	11.068948	11.342687	C	-0.750648	13.545464	16.290378
H	-3.790429	9.617024	10.357799	H	0.197072	15.329617	15.549275
H	-3.769169	11.216056	9.588827	H	-1.500301	11.601596	16.842931
C	-1.484244	9.962280	11.905843	H	-1.463402	14.084931	16.907962
H	-0.398033	9.827786	11.924547	C	0.104251	9.943210	15.397054
H	-1.942645	8.968612	11.970348	H	-0.176466	9.609001	14.392983

H	1.084011	9.513884	15.613259
H	-0.605547	9.526868	16.114773
C	1.104092	13.567509	14.721423
C	2.005364	14.339054	13.796915
H	2.295453	15.299611	14.232259
H	2.909033	13.785388	13.536734
H	1.482040	14.541822	12.855112

TS2.log

SCF (B3PW91) = -4011.78113084
E(SCF)+ZPE(0 K)= -4009.896481
H(298 K)= -4009.786260
G(298 K)= -4010.033827
Lowest Frequency = -104.0887cm-1

Mg	3.769513	10.370759	14.376570
Mg	5.848851	11.247175	10.891295
Mg	4.418702	8.211949	9.968295
Mg	2.138987	10.795890	10.607173
H	4.195000	10.314666	10.478632
N	4.512630	9.708711	16.187573
C	5.812017	9.562469	16.474838
C	6.863097	10.005283	15.653871
H	7.863978	9.823070	16.027344
C	6.785832	10.825540	14.507644
N	5.648999	11.036532	13.854591
C	6.205516	8.941300	17.797822
H	5.747568	9.486992	18.628663
H	7.288104	8.940469	17.933504
H	5.844699	7.910141	17.866675
C	8.038960	11.548845	14.074327
H	8.152111	11.555463	12.987020
H	8.925270	11.103024	14.527478
H	7.989011	12.598385	14.385045
N	5.702306	11.981177	12.797902
C	5.459893	13.246003	13.133174
C	5.437445	14.296753	12.193221
H	5.160096	15.265905	12.590432
C	5.789995	14.275258	10.836766
N	6.106063	13.165362	10.153345
C	5.154128	13.594207	14.568013
H	4.200161	13.134209	14.851633
H	5.066188	14.673046	14.700324
H	5.909114	13.211870	15.258779
C	5.844966	15.626100	10.153307
H	6.841776	16.066158	10.252717
H	5.134244	16.314450	10.614029
H	5.630272	15.550773	9.085051
C	3.558140	9.504972	17.230965
C	3.053846	10.642874	17.909161
C	2.066410	10.453081	18.878461
H	1.659838	11.311578	19.402351
C	1.568232	9.185818	19.163930
H	0.792975	9.062283	19.915333
C	2.047353	8.082861	18.468072
H	1.630373	7.101463	18.674134
C	3.041219	8.218861	17.493995
C	3.534027	12.041002	17.548290
H	3.631067	12.060300	16.456911
C	2.545624	13.150044	17.913246
H	1.540053	12.934276	17.544935
H	2.871106	14.090329	17.457032
H	2.499654	13.312956	18.996828
C	4.918022	12.356483	18.133028
H	4.904513	12.262167	19.224963
H	5.204214	13.385505	17.887500
H	5.692254	11.696559	17.736700
C	3.474572	7.014902	16.675818
H	4.450989	7.245871	16.237978
C	3.634135	5.746484	17.520490
H	4.288749	5.920343	18.380713
H	4.064376	4.936537	16.922818
H	2.672183	5.385254	17.898866
C	2.495709	6.783361	15.514420
H	1.486942	6.590042	15.895150
H	2.801064	5.925194	14.905446
H	2.432447	7.653476	14.853793
C	6.700251	13.324449	8.868524
C	8.037326	13.783207	8.763277
C	8.615338	13.859741	7.493701
H	9.645402	14.192141	7.399216
C	7.900282	13.524688	6.347751
H	8.367913	13.600987	5.369987
C	6.588105	13.082454	6.463149
H	6.036057	12.805711	5.568976
C	5.972119	12.960971	7.713464
C	8.856018	14.183132	9.984895

H	8.173234	14.299851	10.830485
C	9.579330	15.522217	9.784320
H	10.399641	15.433783	9.063687
H	10.014943	15.860782	10.730196
H	8.903291	16.301934	9.419168
C	9.856694	13.093194	10.381340
H	9.352581	12.156621	10.629458
H	10.439049	13.409217	11.254178
H	10.553955	12.879675	9.565580
C	4.573688	12.371425	7.799427
H	4.223042	12.481582	8.834269
C	4.617539	10.871326	7.472105
H	4.929751	10.709212	6.434456
H	3.630925	10.424025	7.613454
H	5.330245	10.342835	8.114083
C	3.561473	13.085896	6.898910
H	3.499238	14.151796	7.137911
H	2.570240	12.637726	7.025576
H	3.829542	12.995127	5.840751
N	4.415599	6.177072	10.336148
C	3.351024	5.378384	10.444861
C	2.032709	5.832478	10.297075
H	1.250708	5.110711	10.500964
C	1.595765	7.118352	9.922399
N	2.435400	8.091670	9.564934
C	3.563560	3.913209	10.743063
H	4.172811	3.456064	9.955332
H	2.618172	3.372952	10.811871
H	4.115634	3.778634	11.679161
C	0.114242	7.394600	9.953912
H	-0.067019	8.271055	10.581705
H	-0.431428	6.542727	10.361986
H	-0.291783	7.627051	8.965201
N	1.846410	9.328062	9.230231
C	1.407465	9.475035	7.986254
C	0.684198	10.613132	7.582431
H	0.356208	10.620565	6.549772
C	0.203258	11.655753	8.395376
N	0.605332	11.877465	9.653592
C	1.668719	8.394514	6.966085
H	2.714124	8.072951	7.018885
H	1.449294	8.750938	5.958303
H	1.063428	7.502556	7.155706
C	-0.870233	12.531464	7.782811

H	-1.751437	12.556644	8.431041
H	-1.168801	12.159084	6.801822
H	-0.535337	13.567060	7.673407
C	5.723299	5.647393	10.515317
C	6.473908	5.266374	9.383288
C	7.826420	4.955494	9.550900
H	8.425153	4.685671	8.684873
C	8.421125	4.998080	10.808401
H	9.476311	4.765522	10.921208
C	7.655073	5.324088	11.924955
H	8.120857	5.328393	12.906574
C	6.300995	5.649325	11.803304
C	5.838044	5.241008	8.002969
H	4.755459	5.329412	8.141836
C	6.287822	6.440959	7.156713
H	7.375698	6.442757	7.025393
H	6.015145	7.391463	7.629166
H	5.824253	6.413522	6.164354
C	6.106088	3.923295	7.266857
H	7.166965	3.802196	7.022288
H	5.547261	3.891559	6.325429
H	5.803619	3.063477	7.873140
C	5.462817	6.003352	13.021329
H	4.411923	5.876474	12.743319
C	5.741854	5.074010	14.206095
H	5.640195	4.021833	13.921586
H	5.040264	5.278437	15.017860
H	6.749843	5.218531	14.609417
C	5.632896	7.467814	13.440527
H	6.622336	7.654325	13.864048
H	4.898936	7.733329	14.207540
H	5.503367	8.159547	12.600859
C	-0.112231	12.860424	10.405952
C	-1.061593	12.425205	11.360491
C	-1.720245	13.391471	12.125738
H	-2.427749	13.083228	12.887312
C	-1.463125	14.747659	11.954641
H	-1.982108	15.478913	12.567765
C	-0.517450	15.160761	11.023308
H	-0.301033	16.219500	10.914136
C	0.180831	14.230721	10.248429
C	-1.330470	10.942156	11.566633
H	-0.358934	10.431786	11.550792
C	-2.174355	10.345840	10.429597

H	-3.122233	10.887390	10.332395
H	-2.406409	9.296327	10.641805
H	-1.653003	10.379942	9.470847
C	-1.967916	10.614347	12.917036
H	-1.426266	11.078961	13.744527
H	-1.958058	9.530754	13.071920
H	-3.013627	10.941322	12.960871
C	1.296457	14.671082	9.321631
H	1.421144	13.899564	8.558910
C	2.616898	14.735008	10.090839
H	2.596873	15.524281	10.849619
H	3.444982	14.934261	9.407675
H	2.831045	13.799649	10.614651
C	1.023849	15.990122	8.595780
H	0.057379	15.974242	8.081224
H	1.804590	16.176388	7.850489
H	1.023856	16.843477	9.282661
C	6.548420	8.605534	9.875295
H	7.253384	7.805105	9.584329
N	7.154774	9.709704	10.200762
C	8.590724	9.724899	10.104688
C	9.358215	9.382612	11.228231
C	10.750913	9.465067	11.130498
H	11.355020	9.216500	11.999734
C	11.362730	9.829156	9.935069
H	12.445596	9.891978	9.873911
C	10.583527	10.086883	8.809978
H	11.058197	10.339798	7.865223
C	9.189129	10.036626	8.872173
C	8.699005	8.824775	12.453555
H	7.735890	9.293876	12.655561
H	9.329773	8.925078	13.339868
H	8.489858	7.760150	12.300168
C	8.346196	10.273996	7.654931
H	7.802773	9.366008	7.367516
H	8.955109	10.592716	6.806866
H	7.598467	11.047417	7.837502
N	2.239502	11.635672	14.016032
C	1.179465	12.220902	14.723235
C	0.343911	11.414932	15.541004
C	-0.121119	14.162436	15.411649
C	-0.682863	12.003995	16.282797
C	-0.927342	13.373723	16.225196
H	-0.295705	15.235296	15.355420
H	-1.313190	11.365747	16.898382
H	-1.732695	13.817390	16.804376
C	0.504366	9.922055	15.567623
H	1.428771	9.616896	16.061387
H	-0.316080	9.452493	16.117637
H	0.523526	9.512322	14.555755
C	0.926728	13.617981	14.664263
C	1.753182	14.551565	13.818856
H	1.799471	15.545312	14.277379
H	2.770052	14.190993	13.672154
H	1.312154	14.670467	12.824087
C	2.427356	9.687235	12.753589
O	1.488115	9.049990	12.375499
C	2.282336	11.852258	12.605271
H	3.183496	12.390682	12.262340
H	1.380434	12.372899	12.250914

TS3.log

SCF (B3PW91) = -4011.80285602
E(SCF)+ZPE(0 K)= -4009.920848
H(298 K)= -4009.810084
G(298 K)= -4010.061221
Lowest Frequency = -851.7669cm-1

Mg	12.689130	7.089686	8.776252
Mg	12.467688	9.314827	6.255252
N	13.976915	5.466069	8.298946
C	15.283822	5.521776	7.998891
C	16.012253	6.698424	7.748145
H	17.058713	6.572408	7.496956
C	15.523535	8.014580	7.706138
N	14.282067	8.283884	8.071466
C	16.089706	4.238630	7.915190
H	15.619540	3.507069	7.253143
H	16.148910	3.764845	8.900312
H	17.104091	4.432463	7.562848
C	16.436486	9.120655	7.240206
H	15.898341	9.825262	6.599352
H	17.293165	8.722911	6.693558
H	16.811049	9.692292	8.096628
N	13.741661	9.550676	7.794998
C	13.689079	10.470183	8.724691
C	12.788536	11.587173	8.604999

H	12.978983	12.373912	9.325233
C	12.227848	12.076912	7.369963
N	12.106456	11.333200	6.289362
C	14.579523	10.382958	9.930554
H	14.766007	9.340573	10.194460
H	14.151449	10.895633	10.787911
H	15.535628	10.863996	9.695106
C	11.781680	13.517677	7.347782
H	11.693110	13.888818	6.325390
H	12.464811	14.161504	7.904905
H	10.792926	13.577113	7.815762
C	13.445033	4.142759	8.398956
C	13.515088	3.446523	9.623473
C	13.167896	2.093782	9.649182
H	13.229569	1.543530	10.584553
C	12.752472	1.440153	8.494011
H	12.513549	0.380271	8.520765
C	12.587307	2.164627	7.318006
H	12.204631	1.662727	6.433912
C	13.897076	4.166154	10.904652
H	14.306190	5.142894	10.622127
C	12.624299	4.413054	11.726877
H	11.878214	4.934887	11.119598
H	12.837091	5.014759	12.618492
H	12.185486	3.462705	12.052855
C	14.959295	3.437154	11.732560
H	14.592355	2.473998	12.104114
H	15.238898	4.037645	12.605097
H	15.865220	3.245476	11.148760
C	11.825018	11.871270	4.997734
C	12.923094	11.999938	4.111256
C	12.680850	12.423812	2.804310
H	13.511087	12.528989	2.113304
C	11.389339	12.705847	2.368633
H	11.217330	13.031045	1.346524
C	10.321416	12.551397	3.241758
H	9.312524	12.745694	2.888098
C	10.511627	12.122585	4.561256
C	14.340247	11.718577	4.583973
H	14.281700	10.990944	5.402903
C	14.962910	12.983050	5.192520
H	15.039693	13.773457	4.437571
H	15.968396	12.774727	5.574903
H	14.355730	13.364024	6.019405
C	15.240535	11.112561	3.505350
H	14.768135	10.245890	3.032737
H	16.185977	10.786272	3.951959
H	15.488827	11.837748	2.722968
C	9.300465	11.884158	5.445346
H	9.646772	11.639798	6.455383
C	8.503763	10.681840	4.918092
H	7.586217	10.528303	5.489858
H	9.096371	9.763520	4.981700
H	8.225673	10.819570	3.867965
C	8.420658	13.135271	5.548346
H	8.023340	13.432530	4.572346
H	8.984253	13.981031	5.953931
H	7.564140	12.952394	6.204201
C	12.400609	8.048292	10.785673
H	12.652673	7.246186	11.517926
N	11.858567	9.069088	11.403912
C	11.601543	8.962447	12.814467
C	10.741074	7.954040	13.300908
C	10.568626	7.839197	14.686171
H	9.908531	7.062174	15.064728
C	11.216666	8.690645	15.572202
H	11.071469	8.581157	16.643191
C	12.027954	9.707193	15.074396
H	12.518712	10.396602	15.756616
C	12.223109	9.865469	13.700883
C	9.998125	6.994114	12.407816
H	10.059692	7.260299	11.352927
H	8.938922	6.958947	12.681092
H	10.394936	5.979820	12.510135
C	13.141452	10.947189	13.209043
H	12.746511	11.450914	12.323565
H	14.125807	10.541397	12.949699
H	13.274346	11.722028	13.967332
Mg	9.657375	6.991620	8.370763
Mg	11.095238	10.582973	10.189004
N	7.816885	6.032415	8.339103
C	6.888986	6.211254	9.265249
C	6.970219	7.226788	10.242631
H	6.216305	7.181513	11.020880
C	7.816849	8.342930	10.291945
N	8.813296	8.582617	9.424291
C	5.672347	5.314212	9.342412
H	5.813756	4.564441	10.128470

H	5.500232	4.783887	8.404304
H	4.781705	5.894212	9.596790
C	7.582735	9.298939	11.433189
H	6.818050	8.919053	12.111957
H	7.287724	10.300094	11.109085
H	8.518885	9.404941	11.988382
N	9.310789	9.911788	9.441304
C	8.504065	10.828393	8.916361
C	8.697438	12.229159	9.067683
H	8.032352	12.835194	8.464327
C	9.424810	12.950528	10.032792
N	10.356047	12.422941	10.841101
C	7.264682	10.390564	8.175110
H	7.468137	9.498482	7.576984
H	6.888635	11.196554	7.543851
H	6.465708	10.117338	8.873026
C	9.114904	14.431173	10.114966
H	9.228669	14.807788	11.133579
H	8.105932	14.654284	9.763712
H	9.821130	14.985006	9.485715
C	7.705018	4.994470	7.375227
C	7.394457	5.341593	6.039627
C	7.392906	4.331209	5.075612
H	7.155890	4.570977	4.043828
C	7.700410	3.014358	5.411661
H	7.705290	2.244962	4.644311
C	8.007947	2.690455	6.727134
H	8.260077	1.664518	6.982499
C	8.012280	3.664696	7.730870
C	7.060432	6.782906	5.690125
H	7.757732	7.409578	6.262049
C	7.249401	7.116417	4.209926
H	8.224594	6.784018	3.842862
H	7.179570	8.197666	4.055778
H	6.480118	6.647536	3.585792
C	5.640746	7.162961	6.134443
H	4.898473	6.530266	5.634878
H	5.427103	8.206840	5.876808
H	5.513869	7.053256	7.213180
C	8.370992	3.282872	9.157161
H	8.196566	4.153515	9.795459
C	7.496099	2.135414	9.678947
H	6.429786	2.351508	9.559186
H	7.706105	1.200226	9.148167
H	7.694717	1.958600	10.741690
C	9.855415	2.931463	9.272458
H	10.493913	3.759398	8.950479
H	10.119921	2.688891	10.307245
H	10.101618	2.064375	8.654354
C	10.740431	13.114202	12.038156
C	10.060166	12.782666	13.236627
C	10.496468	13.329840	14.443864
H	9.985860	13.061982	15.363719
C	11.572702	14.210656	14.489019
H	11.904131	14.624876	15.437379
C	12.206373	14.568500	13.306810
H	13.031518	15.275746	13.334267
C	11.805501	14.039690	12.074638
C	8.844980	11.883004	13.185442
H	9.004639	11.227908	12.327114
C	7.571355	12.696765	12.910886
H	7.405956	13.429440	13.709323
H	6.698530	12.035400	12.871337
H	7.624457	13.235306	11.962463
C	8.651489	11.011453	14.423964
H	9.560277	10.462052	14.671191
H	7.852261	10.284960	14.244970
H	8.355508	11.603290	15.297985
C	12.521273	14.494231	10.820607
H	12.050021	13.966332	9.988502
C	14.013019	14.134578	10.849663
H	14.485385	14.346263	9.883582
H	14.171304	13.078459	11.082942
H	14.541018	14.717461	11.611796
C	12.351416	16.000056	10.576713
H	12.862123	16.583673	11.350273
H	11.298944	16.294469	10.583332
H	12.782300	16.284547	9.609985
O	11.339804	8.042426	7.290655
C	10.604413	7.246959	6.472159
H	9.873163	7.993542	5.419588
C	10.937308	7.404508	5.082670
H	10.524691	6.653592	4.408062
N	12.011473	8.171555	4.691973
C	12.531484	8.027853	3.396150
C	13.885200	7.637410	3.265781
C	14.434859	7.484382	1.990427
H	15.472654	7.171223	1.897814

C	13.675162	7.721684	0.847345
H	14.112387	7.592062	-0.138953
C	12.356215	8.147744	0.981224
H	11.767835	8.374494	0.094446
C	11.773620	8.320404	2.240148
C	14.732875	7.441789	4.490932
H	15.647174	6.885957	4.268088
H	14.198557	6.921879	5.289868
H	15.044416	8.411922	4.901153
C	10.396869	8.908202	2.371901
H	10.427946	9.764215	3.052754
H	9.661744	8.205846	2.777311
H	10.023080	9.256010	1.404957
C	12.892351	3.527592	7.255490
C	12.652190	4.313352	5.976406
H	12.656381	5.376557	6.247135
C	11.283750	3.995468	5.365487
H	10.470863	4.230024	6.058370
H	11.134368	4.580108	4.452934
H	11.193959	2.941981	5.081834
C	13.773988	4.099456	4.952139
H	14.743319	4.407428	5.353819
H	13.841692	3.042817	4.667591
H	13.586955	4.688515	4.047246
H	11.073301	6.123534	9.182296

TS4.log

SCF (B3PW91) = -4011.86629329
E(SCF)+ZPE(0 K)= -4009.979418
H(298 K)= -4009.869769
G(298 K)= -4010.117289
Lowest Frequency = -476.9764cm-1

Mg	12.508248	7.038190	8.150496
Mg	11.695061	9.394896	5.862240
N	14.058565	5.636081	8.307443
C	15.294401	5.760402	7.818776
C	15.806188	6.924805	7.214444
H	16.817142	6.854336	6.828093
C	15.143149	8.142720	6.981755
N	13.889133	8.313246	7.361932
C	16.248241	4.582109	7.834134
H	16.241428	4.109449	6.845950
H	15.969334	3.825376	8.568447
H	17.272154	4.910188	8.030345
C	15.860492	9.223885	6.220591
H	15.553088	9.162142	5.170598
H	16.943601	9.097643	6.269562
H	15.594262	10.222105	6.575790
N	13.240776	9.551245	7.190491
C	13.248042	10.350344	8.232164
C	12.366227	11.481957	8.330030
H	12.652536	12.199363	9.096089
C	11.676718	12.078698	7.225528
N	11.369808	11.431815	6.109471
C	14.165892	10.056596	9.383052
H	13.987443	9.035695	9.734392
H	14.002291	10.750878	10.204273
H	15.211689	10.119358	9.067169
C	11.397626	13.557120	7.324666
H	12.184679	14.110055	6.799425
H	11.381532	13.880090	8.365587
H	10.443692	13.813806	6.858869
C	13.689186	4.437843	8.984471
C	13.803165	4.389138	10.395455
C	13.406153	3.221821	11.053242
H	13.491061	3.163290	12.134196
C	12.904158	2.130074	10.349493
H	12.605124	1.230370	10.880643
C	12.770824	2.203464	8.968109
H	12.360330	1.357368	8.422261
C	14.382949	5.562766	11.171373
H	14.164923	6.467393	10.590309
C	13.769457	5.735373	12.564245
H	12.675920	5.740842	12.539732
H	14.086074	6.688187	12.997969
H	14.091682	4.943774	13.250593
C	15.910953	5.455329	11.283886
H	16.195336	4.525732	11.790520
H	16.310336	6.294487	11.864949
H	16.391487	5.468601	10.303692
C	11.126578	12.189959	4.919348
C	12.248354	12.661999	4.200063
C	12.034956	13.294632	2.971651
H	12.889260	13.652129	2.403334
C	10.755548	13.451106	2.454934
H	10.608596	13.937972	1.494906

C	9.662099	12.974374	3.170167
H	8.667270	13.097473	2.755397
C	9.821721	12.343288	4.406798
C	13.675031	12.420620	4.667348
H	13.646115	12.006389	5.679793
C	14.503110	13.710067	4.715725
H	14.658569	14.131005	3.716612
H	15.491122	13.508661	5.143055
H	14.018004	14.477748	5.327085
C	14.341231	11.369386	3.769355
H	13.820696	10.407806	3.822587
H	15.382145	11.210252	4.066670
H	14.333065	11.681811	2.720104
C	8.627590	11.791354	5.161412
H	8.878526	11.825484	6.227786
C	8.375946	10.325739	4.785494
H	7.544180	9.909342	5.361876
H	9.242401	9.682937	4.967585
H	8.132709	10.242737	3.721162
C	7.350487	12.605298	4.935201
H	7.018882	12.555440	3.893017
H	7.493399	13.659214	5.194476
H	6.531834	12.207253	5.539919
C	11.759551	7.610526	10.074886
H	11.917968	6.691051	10.671677
N	11.373137	8.639208	10.887641
C	11.210012	8.302604	12.269212
C	10.204141	7.412003	12.708481
C	10.208320	6.972216	14.036564
H	9.440959	6.270717	14.357062
C	11.148679	7.438559	14.948878
H	11.135578	7.093372	15.978967
C	12.086734	8.379476	14.532173
H	12.816234	8.769168	15.238291
C	12.133809	8.816901	13.206098
C	9.123505	6.956443	11.776212
H	8.764564	7.798436	11.180622
H	8.276955	6.531394	12.322686
H	9.485756	6.214937	11.055431
C	13.224108	9.755594	12.776787
H	12.826091	10.652723	12.292320
H	13.897996	9.266456	12.067044
H	13.807196	10.098018	13.635180
Mg	9.372093	7.649587	8.337518
Mg	10.913824	10.485585	10.119415
N	7.681413	6.547918	7.987015
C	6.547801	6.796534	8.643260
C	6.355028	7.900660	9.496869
H	5.426500	7.889521	10.057731
C	7.184693	9.006980	9.762103
N	8.338951	9.224318	9.120536
C	5.385054	5.835469	8.528031
H	5.317237	5.413327	7.522550
H	4.442877	6.328906	8.773949
H	5.517926	4.996772	9.218402
C	6.734098	9.891430	10.904341
H	5.656673	10.070754	10.865639
H	7.251982	10.846267	10.952238
H	6.945148	9.356517	11.837875
N	9.016681	10.438140	9.338696
C	8.400925	11.562734	8.983002
C	8.851158	12.839244	9.388492
H	8.315510	13.674150	8.949791
C	9.700587	13.167227	10.460883
N	10.521153	12.298247	11.058917
C	7.117167	11.526972	8.191715
H	7.105112	10.690292	7.490012
H	6.991356	12.467074	7.654691
H	6.247592	11.412941	8.846321
C	9.630998	14.603688	10.933145
H	9.900205	14.692299	11.987372
H	8.633503	15.022307	10.785913
H	10.336645	15.214062	10.357887
C	7.802778	5.348513	7.226428
C	7.792020	5.439008	5.814408
C	7.930621	4.266174	5.068913
H	7.918759	4.314413	3.984938
C	8.093811	3.032186	5.692487
H	8.201479	2.130457	5.096007
C	8.153463	2.964020	7.078301
H	8.329572	2.005643	7.559151
C	8.025596	4.110391	7.869538
C	7.622578	6.789572	5.138040
H	8.169715	7.520972	5.747012
C	8.211021	6.846882	3.727675
H	9.255638	6.524627	3.718976
H	8.172013	7.873249	3.352304
H	7.646030	6.227128	3.022395

C	6.148169	7.218134	5.121745
H	5.541099	6.476658	4.590182
H	6.030883	8.179705	4.609816
H	5.750810	7.324631	6.133849
C	8.198964	3.998547	9.375794
H	7.926321	4.960400	9.821090
C	7.304666	2.917457	9.997097
H	6.255071	3.034799	9.709451
H	7.618573	1.913533	9.690868
H	7.367067	2.958181	11.089689
C	9.670272	3.732951	9.721580
H	10.313511	4.539959	9.356128
H	9.809838	3.664590	10.806030
H	10.018934	2.795095	9.279001
C	11.081966	12.580845	12.341491
C	10.461122	11.982336	13.466926
C	11.070663	12.110825	14.715643
H	10.617963	11.640083	15.581906
C	12.255845	12.824477	14.870936
H	12.718601	12.907311	15.850605
C	12.836874	13.436799	13.768999
H	13.754101	14.006608	13.894562
C	12.268922	13.329689	12.494313
C	9.130851	11.261685	13.318859
H	9.157401	10.718635	12.365828
C	7.981504	12.280394	13.246183
H	7.984903	12.916310	14.138689
H	7.015097	11.766468	13.202500
H	8.057231	12.924742	12.367677
C	8.847360	10.243291	14.421081
H	9.688767	9.565939	14.569643
H	7.974204	9.640556	14.150892
H	8.618052	10.733076	15.375032
C	12.952392	14.020423	11.327929
H	12.363670	13.804219	10.430277
C	14.373078	13.486497	11.104914
H	14.797585	13.881041	10.175297
H	14.388118	12.396283	11.053675
H	15.036420	13.780007	11.925502
C	12.996714	15.544330	11.513029
H	13.638632	15.814479	12.358496
H	12.005858	15.961531	11.707263
H	13.405764	16.028647	10.619420
O	10.742581	7.953857	6.937839
C	10.992470	6.720701	6.286545
H	10.394123	5.864940	6.588650
C	11.596944	6.751222	5.057285
H	11.728662	5.805596	4.531945
N	11.998364	7.912892	4.488628
C	12.758835	7.880478	3.305899
C	13.918721	7.079547	3.164371
C	14.659946	7.167022	1.980766
H	15.556892	6.558417	1.883071
C	14.287806	8.018784	0.945971
H	14.883123	8.074709	0.038695
C	13.141167	8.796989	1.087311
H	12.830104	9.459075	0.282059
C	12.369207	8.737130	2.247522
C	14.397404	6.158833	4.257266
H	14.016799	5.137711	4.128438
H	14.082433	6.497272	5.244941
H	15.489331	6.097790	4.264178
C	11.131379	9.576559	2.381616
H	11.278967	10.410581	3.077716
H	10.294514	8.986314	2.765881
H	10.843923	10.018916	1.423994
C	13.142778	3.353493	8.265385
C	12.941992	3.420369	6.761495
H	13.222284	4.429152	6.440235
C	11.471097	3.206081	6.391812
H	10.811921	3.862767	6.961793
H	11.299970	3.395249	5.326640
H	11.148599	2.179350	6.596621
C	13.828575	2.414614	6.016309
H	14.884707	2.541274	6.266886
H	13.549596	1.385274	6.269566
H	13.717059	2.532218	4.932461
H	10.554994	6.703528	9.381696

TS5.log

SCF (B3PW91) = -4125.16046927
E(SCF)+ZPE(0 K)= -4123.260575
H(298 K)= -4123.148650
G(298 K)= -4123.400309
Lowest Frequency = -241.6893cm-1

Mg	12.636824	6.829369	8.115108

Mg	11.449602	9.345508	6.117806
N	14.265254	5.542289	8.178645
C	15.453179	5.758967	7.611819
C	15.845325	6.971146	7.011322
H	16.820978	6.966631	6.538485
C	15.105321	8.154993	6.860559
N	13.882968	8.278581	7.361289
C	16.464124	4.636745	7.507355
H	16.317787	3.870064	8.269268
H	17.485294	5.019521	7.572766
H	16.353205	4.156713	6.528520
C	15.695742	9.254186	6.023815
H	15.232950	9.200744	5.031856
H	16.773899	9.134989	5.906273
H	15.487322	10.245701	6.427767
N	13.167546	9.485826	7.255423
C	13.331051	10.396881	8.182235
C	12.402645	11.499438	8.317675
H	12.715554	12.229662	9.058326
C	11.725129	12.098750	7.204042
N	11.418377	11.432737	6.098076
C	14.502709	10.361067	9.124398
H	14.877071	9.346588	9.269975
H	14.242793	10.794271	10.087162
H	15.312180	10.967104	8.700364
C	11.450306	13.581940	7.276236
H	12.060231	14.110957	6.536971
H	11.674600	13.980060	8.265366
H	10.403499	13.790745	7.047098
C	14.017990	4.293821	8.830472
C	14.216613	4.214949	10.228596
C	13.917518	3.014213	10.874450
H	14.059314	2.936321	11.948160
C	13.427336	1.918406	10.168171
H	13.193795	0.994469	10.690337
C	13.227031	2.014288	8.796616
H	12.832795	1.161348	8.249876
C	14.775550	5.403018	10.994402
H	14.495839	6.296724	10.425104
C	14.211399	5.549981	12.410715
H	13.120529	5.515764	12.406636
H	14.521461	6.511120	12.836388
H	14.587611	4.767658	13.080232
C	16.310550	5.360082	11.036211
H	16.655935	4.443773	11.528827
H	16.702402	6.215228	11.598995
H	16.746412	5.390258	10.035072
C	11.262885	12.173884	4.878421
C	12.418045	12.345704	4.073827
C	12.280348	12.997792	2.846636
H	13.152581	13.140671	2.217892
C	11.043494	13.448484	2.400061
H	10.956885	13.941286	1.435650
C	9.919179	13.252668	3.188773
H	8.952390	13.587459	2.826145
C	10.006318	12.624706	4.436155
C	13.779938	11.824127	4.506782
H	13.603456	10.941047	5.129278
C	14.525193	12.845766	5.379761
H	14.626338	13.802120	4.854419
H	15.532569	12.485023	5.615625
H	14.010720	13.027092	6.326356
C	14.659542	11.393959	3.329469
H	14.145747	10.673162	2.687206
H	15.576505	10.927593	3.701047
H	14.971364	12.246909	2.716766
C	8.741478	12.374458	5.233107
H	9.028178	12.175231	6.271743
C	8.039590	11.120550	4.691434
H	7.076541	10.954791	5.181283
H	8.655522	10.229783	4.851760
H	7.864055	11.209288	3.614029
C	7.811860	13.593353	5.224780
H	7.408234	13.793257	4.227387
H	8.337724	14.492530	5.561293
H	6.951769	13.437936	5.881908
C	12.272705	7.934016	10.184853
H	12.809722	8.584724	9.488840
N	11.039874	8.568380	10.648665
C	10.935698	8.430634	12.080629
C	9.943634	7.633661	12.704727
C	10.010683	7.401678	14.083576
H	9.258996	6.759014	14.535550
C	10.992024	7.976175	14.880718
H	11.022096	7.781610	15.948914
C	11.904616	8.842103	14.290838
H	12.644312	9.352842	14.902545
C	11.891668	9.076892	12.914258

C	8.795173	7.027047	11.956435
H	9.118901	6.253851	11.254776
H	8.236108	7.779609	11.402338
H	8.090818	6.553409	12.645503
C	12.921818	10.024584	12.364947
H	12.493737	10.763804	11.674964
H	13.716093	9.504924	11.821382
H	13.370810	10.616975	13.165578
Mg	9.569669	7.572770	8.971546
Mg	10.759476	10.558707	10.003411
N	7.882693	6.408589	8.452768
C	6.676530	6.638026	8.975681
C	6.351886	7.794258	9.708079
H	5.399852	7.782080	10.227355
C	7.081290	8.993088	9.821776
N	8.258049	9.169416	9.215090
C	5.553452	5.648160	8.754232
H	5.854700	4.633135	9.017143
H	5.279703	5.620681	7.694465
H	4.667372	5.919323	9.330715
C	6.492696	10.013123	10.771728
H	6.540197	9.591558	11.782003
H	5.438201	10.200822	10.550166
H	7.026173	10.960480	10.782242
N	8.870043	10.425359	9.259218
C	8.225461	11.484919	8.784774
C	8.659230	12.802050	9.070028
H	8.125558	13.588101	8.545668
C	9.490141	13.226518	10.124391
N	10.306904	12.418907	10.816106
C	6.961131	11.318159	7.981348
H	6.972286	10.378119	7.425271
H	6.846797	12.154418	7.294530
H	6.072841	11.313137	8.618932
C	9.418698	14.699037	10.465520
H	9.621345	14.873414	11.524411
H	8.442896	15.121046	10.217603
H	10.175612	15.245024	9.890611
C	7.985646	5.320270	7.538081
C	7.789267	5.591091	6.160118
C	7.835834	4.521998	5.263278
H	7.682827	4.699740	4.203917
C	8.098455	3.224157	5.697876
H	8.126453	2.406217	4.982948
C	8.362614	2.988838	7.039754
H	8.611868	1.983653	7.369130
C	8.329030	4.026050	7.979994
C	7.617805	7.025043	5.669327
H	8.299437	7.637175	6.277539
C	8.018872	7.199046	4.202951
H	9.026247	6.820868	4.010220
H	7.997598	8.260692	3.941504
H	7.319626	6.694436	3.526581
C	6.202612	7.587763	5.873387
H	5.455697	6.940016	5.400077
H	6.125310	8.578553	5.411020
H	5.951574	7.700563	6.929287
C	8.700300	3.735348	9.425006
H	8.733300	4.693968	9.952369
C	7.679403	2.834772	10.133832
H	6.683146	3.283274	10.173347
H	7.590898	1.868119	9.624755
H	7.998397	2.641586	11.163839
C	10.093508	3.104254	9.513897
H	10.845565	3.712251	9.011195
H	10.408648	3.008742	10.555963
H	10.114089	2.107453	9.060008
C	10.799740	12.793959	12.105566
C	10.116953	12.281324	13.237400
C	10.659442	12.495746	14.505566
H	10.153115	12.093697	15.376903
C	11.841809	13.210090	14.675608
H	12.251101	13.361235	15.670797
C	12.486531	13.737001	13.564926
H	13.401402	14.309267	13.697109
C	11.984297	13.542317	12.273388
C	8.786358	11.566795	13.076870
H	8.832823	10.997194	12.141365
C	7.652057	12.595885	12.941647
H	7.638005	13.258652	13.814330
H	6.680174	12.093887	12.884122
H	7.764539	13.212757	12.046914
C	8.469878	10.585475	14.203541
H	9.306039	9.911514	14.393896
H	7.601421	9.976307	13.932687
H	8.218552	11.104553	15.136033
C	12.736863	14.137506	11.099823
H	12.191749	13.848663	10.196153

C	14.160713	13.576081	11.000832
H	14.632505	13.862609	10.054427
H	14.162929	12.486620	11.071861
H	14.789369	13.953084	11.814480
C	12.782334	15.670448	11.159413
H	13.369961	16.009182	12.019502
H	11.783723	16.103855	11.252605
H	13.251857	16.077077	10.256718
O	10.763884	7.853171	7.338857
C	10.918403	6.648642	6.602103
H	10.330241	5.797052	6.931114
C	11.383676	6.714877	5.307599
H	11.455592	5.776632	4.757356
N	11.719965	7.882994	4.720278
C	12.411669	7.900784	3.493528
C	13.581006	7.135429	3.262401
C	14.264590	7.287238	2.050454
H	15.170060	6.705560	1.889443
C	13.822726	8.163091	1.065763
H	14.374460	8.271298	0.136160
C	12.659208	8.896294	1.287328
H	12.288817	9.574220	0.521754
C	11.946115	8.775244	2.480497
C	14.124111	6.165041	4.277519
H	13.741900	5.150843	4.108192
H	13.858077	6.446504	5.296266
H	15.215032	6.114751	4.227476
C	10.672178	9.543409	2.674670
H	10.747760	10.262521	3.494125
H	9.842549	8.873239	2.912489
H	10.415634	10.121368	1.783273
C	13.510589	3.196386	8.105179
C	13.279200	3.271949	6.606069
H	13.583904	4.271180	6.281982
C	11.798843	3.104120	6.247383
H	11.174640	3.843332	6.751204
H	11.645983	3.212196	5.167581
H	11.424449	2.116935	6.538552
C	14.129555	2.244080	5.847580
H	15.187241	2.316723	6.115585
H	13.802842	1.221978	6.069809
H	14.037698	2.392725	4.765791
C	11.215104	6.306729	9.905077
O	11.404879	5.556354	10.813878
H	12.949493	7.620467	10.978922

TS6.log

SCF (B3PW91) = -4125.22175498
E(SCF)+ZPE(0 K)= -4123.323988
H(298 K)= -4123.211910
G(298 K)= -4123.466899
Lowest Frequency = -815.0703cm-1

Mg	12.551102	6.708460	7.937389
Mg	11.615271	9.300316	5.780031
N	14.134138	5.409787	8.165588
C	15.393631	5.669957	7.788528
C	15.850332	6.907477	7.302025
H	16.893207	6.937355	7.006776
C	15.121003	8.098062	7.078459
N	13.833229	8.173600	7.358166
C	16.422669	4.559246	7.805448
H	16.176488	3.776726	8.525414
H	17.419469	4.948082	8.025746
H	16.461193	4.093609	6.814460
C	15.817849	9.282119	6.463161
H	16.874564	9.078104	6.284367
H	15.730349	10.174111	7.089560
H	15.334310	9.517364	5.508924
N	13.134738	9.383026	7.173406
C	13.116386	10.236482	8.184460
C	12.325985	11.419520	8.171532
H	12.443474	12.058394	9.037995
C	11.648822	11.992855	7.081093
N	11.372104	11.326591	5.952062
C	13.964906	10.001637	9.409102
H	14.245637	8.952645	9.500595
H	13.425258	10.294787	10.312927
H	14.882050	10.598023	9.352985
C	11.216960	13.434740	7.224074
H	11.086497	13.913122	6.251407
H	11.935801	14.012560	7.809587
H	10.254978	13.463052	7.752025
C	13.780193	4.127012	8.678636
C	13.698780	3.949730	10.081301
C	13.267481	2.717469	10.578135
H	13.206361	2.565096	11.651359

C	12.902003	1.681983	9.722803
H	12.564004	0.732406	10.128706
C	12.963235	1.873958	8.347522
H	12.667151	1.068711	7.679712
C	14.096180	5.073766	11.022926
H	13.932547	6.009977	10.473996
C	13.266353	5.115616	12.310025
H	12.192348	5.118908	12.100039
H	13.495987	6.017838	12.886528
H	13.480904	4.260750	12.960074
C	15.593852	5.015681	11.356505
H	15.841823	4.066691	11.845379
H	15.868264	5.830971	12.035121
H	16.207716	5.105135	10.457333
C	11.163756	12.036439	4.734542
C	12.305040	12.347513	3.956484
C	12.117334	12.940106	2.705008
H	12.982394	13.191539	2.098138
C	10.841228	13.201031	2.218708
H	10.712177	13.651577	1.238357
C	9.728333	12.883932	2.991130
H	8.737898	13.092548	2.599327
C	9.865005	12.308303	4.258033
C	13.707819	12.061122	4.469043
H	13.619362	11.341751	5.290596
C	14.332558	13.334660	5.055702
H	14.441336	14.105468	4.283798
H	15.325301	13.123099	5.468501
H	13.711273	13.745830	5.856990
C	14.617137	11.432844	3.407856
H	14.182565	10.515474	2.999028
H	15.589400	11.185850	3.847539
H	14.809622	12.115305	2.572935
C	8.645310	11.931516	5.081458
H	8.931938	11.977027	6.139380
C	8.228305	10.489262	4.764693
H	7.323066	10.200836	5.311142
H	9.005595	9.761942	5.019186
H	8.021667	10.382876	3.694853
C	7.466399	12.885266	4.870688
H	7.068858	12.821935	3.852249
H	7.755116	13.924352	5.057480
H	6.645382	12.628893	5.544483
C	11.902909	8.053988	10.914161
H	12.765570	7.495042	11.280121
N	11.426561	9.115621	11.647738
C	11.454482	8.860978	13.039569
C	10.510374	7.971204	13.601602
C	10.589729	7.659213	14.962333
H	9.865178	6.970436	15.391690
C	11.563477	8.237448	15.771902
H	11.608920	7.995929	16.830471
C	12.470300	9.140449	15.219036
H	13.230124	9.599499	15.847702
C	12.435959	9.455133	13.857995
C	9.422598	7.374448	12.747849
H	9.756212	6.464219	12.230667
H	9.102346	8.073740	11.967659
H	8.551185	7.101464	13.350019
C	13.453650	10.374302	13.247792
H	12.966405	11.171085	12.680879
H	14.102306	9.832249	12.549394
H	14.081021	10.838844	14.012568
Mg	9.120181	8.111906	8.002914
Mg	10.375338	10.382682	10.404078
N	7.690824	6.608467	8.018720
C	6.562989	6.646316	8.745323
C	6.228561	7.668751	9.645064
H	5.341001	7.496464	10.243015
C	6.898139	8.887622	9.897066
N	7.889294	9.325767	9.132853
C	5.579830	5.499933	8.641578
H	5.977881	4.593858	9.109476
H	5.389124	5.248533	7.594340
H	4.633010	5.746824	9.124333
C	6.469796	9.688550	11.102596
H	5.680280	9.176573	11.654473
H	6.124748	10.692867	10.843097
H	7.330445	9.828498	11.764855
N	8.505938	10.536938	9.528330
C	7.956741	11.655849	9.079418
C	8.419173	12.930322	9.464564
H	7.879191	13.767818	9.036793
C	9.348697	13.258968	10.468680
N	10.175932	12.386881	11.056771
C	6.755888	11.593018	8.170386
H	6.924215	10.877042	7.362200
H	6.535632	12.574970	7.751513

H	5.868517	11.245067	8.708511	H	9.166062	10.733030	12.776125
C	9.336073	14.718101	10.876352	C	7.854435	12.350909	13.288940
H	9.699616	14.864570	11.894174	H	7.805593	13.184273	13.998788
H	8.328821	15.132259	10.799056	H	6.948750	11.747394	13.415612
H	9.982626	15.291155	10.203135	H	7.842207	12.756306	12.276130
C	7.932181	5.444286	7.228031	C	8.930390	10.754330	14.886745
C	7.730711	5.495794	5.831903	H	9.827069	10.196839	15.161613
C	8.015250	4.359325	5.067129	H	8.105719	10.039483	14.800617
H	7.850647	4.386662	3.993495	H	8.679574	11.443428	15.702078
C	8.511506	3.201441	5.651278	C	12.632524	14.094876	10.888945
H	8.742275	2.333876	5.039538	H	11.962949	13.781848	10.082156
C	8.729812	3.167137	7.024862	C	14.012291	13.470127	10.638237
H	9.146615	2.271105	7.475577	H	14.373235	13.709044	9.631660
C	8.440617	4.269192	7.832382	H	13.982839	12.384513	10.742407
C	7.212579	6.744416	5.140976	H	14.746359	13.847386	11.358594
H	7.210740	7.554739	5.878899	C	12.745391	15.625447	10.847277
C	8.121793	7.166629	3.981825	H	13.487222	15.984896	11.568647
H	9.133527	7.371212	4.342087	H	11.796820	16.114118	11.081793
H	7.732892	8.070481	3.504295	H	13.067162	15.957194	9.853812
H	8.181508	6.393018	3.209236	O	10.364798	7.925160	6.517371
C	5.764005	6.553728	4.671921	C	10.945231	6.686619	6.182884
H	5.694148	5.742427	3.938419	H	10.335266	5.806663	6.386855
H	5.387226	7.468020	4.199886	C	11.716058	6.677062	5.017905
H	5.103241	6.306635	5.508773	H	12.030796	5.715429	4.614041
C	8.743012	4.205138	9.320282	N	12.057876	7.822404	4.416920
H	8.203726	5.019501	9.814299	C	12.828394	7.841810	3.238830
C	8.305365	2.890437	9.974660	C	14.065516	7.170354	3.107866
H	7.258622	2.656915	9.754436	C	14.796039	7.326530	1.924201
H	8.915640	2.046190	9.636653	H	15.755125	6.820307	1.834403
H	8.420160	2.956466	11.061602	C	14.330649	8.115603	0.878505
C	10.237130	4.449445	9.545342	H	14.917584	8.226944	-0.029039
H	10.508220	5.442916	9.175591	C	13.102304	8.760363	1.007423
H	10.496103	4.414595	10.608574	H	12.721080	9.372276	0.193087
H	10.851667	3.706727	9.026096	C	12.342194	8.635709	2.170682
C	10.873416	12.790946	12.239095	C	14.629982	6.305032	4.202434
C	10.379641	12.325320	13.485014	H	14.379214	5.247871	4.047807
C	11.072011	12.664335	14.649411	H	14.253894	6.589249	5.185418
H	10.716149	12.303324	15.607728	H	15.720771	6.375684	4.235892
C	12.219072	13.450576	14.609418	C	11.019202	9.339571	2.290590
H	12.742038	13.700036	15.528618	H	11.083874	10.207054	2.956890
C	12.690813	13.907870	13.387881	H	10.249222	8.678867	2.698503
H	13.589487	14.518420	13.354847	H	10.684760	9.716894	1.320443
C	12.039863	13.588378	12.191229	C	13.397601	3.087206	7.803240
C	9.103547	11.497899	13.562411	C	13.423092	3.266549	6.296442

H	13.780838	4.281029	6.095148	H	15.381932	2.323441	6.045335
C	12.013595	3.144701	5.708432	H	14.019845	1.247793	5.719376
H	11.320461	3.844208	6.180342	H	14.453392	2.495091	4.543514
H	12.019089	3.340193	4.630136	C	11.542481	7.880511	9.528973
H	11.605066	2.139215	5.857136	O	10.600682	8.745647	9.182150
C	14.377818	2.278197	5.614775	H	11.136865	7.090572	10.680724

7) References

[S1] Intemann J.; Spielmann J.; Sirsch P.; Harder S. *Chem. Eur. J.*, **2013**, *19*, 8478 – 8489.

[S2] Yow S.; Gates S. J.; White A. J. P.; Crimmin M. R. *Angew. Chem. Int. Ed.*, **2012**, *51*, 12559 – 12563.

[S3] Green S. P.; Jones C.; Stasch A. *Angew. Chem. Int. Ed.*, **2008**, *47*, 9079–90.

[S4] Pergosin P. S.; Kumar P. G. A.; Fernandez I. *Chem. Rev.,* **2005**, *105*, 2977–2998.

[S5] Dolomanov O.V.; Bourhis L. J.; Gildea R. J.; Howard J. A. K.; Puschmann H. *J. Appl. Cryst.*, **2009**, *42*, 339-341.

[S6] SHELXTL v5.1, Bruker AXS, Madison, WI, 1998.

[S7] SHELX-2013, Sheldrick G. M. *Acta Cryst.*, **2015**, *C71*, 3-8.

[S8] Spek A. L. (2003, 2009) PLATON, A Multipurpose Crystallographic Tool, Utrecht University, Utrecht, The Netherlands. See also Spek A. L. *Acta. Cryst.*, 2015, **C71**, 9-18.

[S9] Frisch, M. J.; Trucks, G. W.; Schlegel, H. B.; Scuseria, G. E.; Robb, M. A.; Cheeseman, J. R.; Scalmani, G.; Barone, V.; Mennucci, B.; Petersson, G. A.; Nakatsuji, H.; Caricato, M.; Li, X.; Hratchian, H. P.; Izmaylov, A. F.; Bloino, J.; Zheng, G.; Sonnenberg, J. L.; Hada, M.; Ehara, M.; Toyota, K.; Fukuda, R.; Hasegawa, J.; Ishida, M.; Nakajima, T.; Honda, Y.; Kitao, O.; Nakai, H.; Vreven, T.; Montgomery, J. A., Jr.; Peralta, J. E.; Ogliaro, F.; Bearpark, M.; Heyd, J. J.; Brothers, E.; Kudin, K. N.; Staroverov, V. N.; Kobayashi, R.; Normand, J.; Raghavachari, K.; Rendell, A.; Burant, J. C.; Iyengar, S. S.; Tomasi, J.; Cossi, M.; Rega, N.; Millam, J. M.; Klene, M.; Knox, J. E.; Cross, J. B.; Bakken, V.; Adamo, C.; Jaramillo, J.; Gomperts, R.; Stratmann, R. E.; Yazyev, O.; Austin, A. J.; Cammi, R.; Pomelli, C.; Ochterski, J. W.; Martin, R. L.; Morokuma, K.; Zakrzewski, V. G.; Voth, G. A.; Salvador, P.; Dannenberg, J. J.; Dapprich, S.; Daniels, A. D.; Farkas, Ö.; Foresman, J. B.; Ortiz, J. V.; Cioslowski, J.; Fox, D. J. Gaussian 09, Revision D.01; Gaussian, Inc., Wallingford, CT, 2009.

[S10] Glendening E. D.; Landis C. R.; Weinhold. F.; *J. Comput. Chem.*, **2013**, *34*, 1429–1437.

[S11] Keith T. A.; AIMALL (Version 19.10.12). TK Gristmill Software, Overland Park KS, USA., 2019.

[S12] Cortés-Guzmán F.; Bader R. F. W. *Coord. Chem. Rev.*, **2005**, *249*, 633–662.

[S13] Stadler B., Gorgas N., Elliott S. J., Crimmin M.R. *ChemRxiv.*, **2024**. (DOI: 10.26434/chemrxiv-2024-7vlzq)

[S14] Gerbitz K.D. *Hoppe Seylers Z Physiol Chem.* **1977**, Nov; 358(11): 1491-7.
